# ‘An adrenaline-fueled emergency’: a qualitative thematic analysis of reviews of deaths in England where DNACPR (Do Not Attempt Cardiopulmonary Resuscitation) recommendations are not implemented for people with a learning disability

**DOI:** 10.1136/bmjopen-2025-107222

**Published:** 2026-06-25

**Authors:** Christina Roberts, Nicola Ditzel, Adam White, Rory Sheehan, André Strydom, Alex Ruck-Keene, Umesh Chauhan

**Affiliations:** 1 Faculty of Health, Science, Social Care and Education, Kingston University London, Kingston upon Thames, UK; 2 School of Medicine and Dentistry, University of Lancashire, Preston, UK; 3 Institute of Psychiatry, Psychology and Neuroscience, King’s College London, London, UK

**Keywords:** Disabled Persons, Adult palliative care, Cardiopulmonary Resuscitation

## Abstract

**Objectives:**

To investigate the circumstances and contributory factors in cases where cardiopulmonary resuscitation (CPR) is administered to people with a learning disability despite a Do Not Attempt Cardiopulmonary Resuscitation (DNACPR) recommendation being in place. Limited understanding exists around why DNACPR recommendations are sometimes not followed in practice. This study aims to address this gap by examining what occurs when CPR is performed contrary to an established DNACPR recommendation.

**Design:**

Qualitative study using inductive thematic analysis of secondary data derived from comprehensive reviews of deaths of people with a learning disability.

**Setting:**

England, deaths occurring in hospital or out of hospital.

**Participants:**

21 adults (aged ≥18 years) with a learning disability who died between 2020 and 2022 and whose deaths were reviewed through the Learning from Lives and Deaths (LeDeR) programme. Cases were included where a DNACPR recommendation was in place but not followed.

**Results:**

Three themes describe pressures to respond to an emergency, knowledge and awareness of DNACPR and decision-making processes. Two cross-cutting factors shaped how DNACPR recommendations were enacted in practice: breakdowns in information sharing and unclear or inaccessible documentation. The challenges described in each theme, compounded by the cross-cutting factors, created uncertainty and contributed to a default to life-preserving action despite active DNACPR recommendations being in place.

**Conclusions:**

DNACPR recommendations for people with a learning disability are at risk of not being followed as intended in emergency contexts due to challenges in communication and documentation. These issues create uncertainty and may contribute to a default to attempt resuscitation. Improvements are needed in how DNACPR decisions are documented, shared and accessed across care settings, alongside training to support staff in interpreting and applying recommendations in complex situations, including within the legal framework of the Mental Capacity Act (2005).

**STRENGTHS AND LIMITATIONS OF THIS STUDY**

- Three researchers collaboratively undertook the thematic analysis, enhancing the rigour of the findings.
- Reflective qualitative insights highlight key areas of improvement for practice related to Do Not Attempt Cardiopulmonary Resuscitation (DNACPR) recommendations.
- The dataset may not represent certain communities, for example, people with a learning disability from ethnic minority communities.
- The use of secondary retrospective data meant that analysis was limited to secondhand accounts of events, potentially introducing recall and observer bias.
- The data source did not include sufficient detail to assess the appropriateness of DNACPR recommendations, and evaluation of the quality of the original DNACPR decision is beyond the scope of this paper.

## Introduction

Do Not Attempt Cardiopulmonary Resuscitation (DNACPR), also known as DNR (do not resuscitate) or DNAR (do not attempt resuscitation), instructs healthcare professionals whether to perform cardiopulmonary resuscitation (CPR) in the event of a cardiac or respiratory arrest.^1^ DNACPR recommendations are intended to protect the individual from unnecessary suffering or loss of dignity in the dying process caused by receiving CPR, given that CPR has a low success rate of between 11% and 18% in adults^2^ and can lead to adverse clinical outcomes such as brain damage and increased physical disability.^3^

DNACPR decision-making is ethically complex and inconsistently implemented.^4^ Case law has confirmed that patients must be included in discussions unless it would cause them harm. The decision must be clearly recorded in medical records through specific forms including Treatment Escalation Plan (TEPs) or a Recommended Summary Plan for Emergency Care and Treatment (ReSPECT).^1^ TEPs are clinical communication tools which can be used to document a patient’s preferences and medical recommendations for care, and similarly ReSPECT is a personalised, non-legally binding document outlining a person’s preferences for care, including CPR decisions, if they become critically ill or incapacitated. Clinicians must be able to justify DNACPR decisions that conflict with a patient’s wishes.^5^

DNACPR recommendations vary in how they are made, recorded and communicated across settings, with ongoing concerns about how patients are involved and whether staff understand the implications of an active recommendation.^4 6^

### DNACPR decision-making for people with a learning disability

For people with a learning disability, DNACPR decision-making can be more complex due to communication barriers or lack of capacity.^7^ In such cases, the Mental Capacity Act (2005) requires that legal proxies or those close to the person be involved, and decisions must follow a best interest framework.^8 9^ Many of the challenges identified in DNACPR decision-making for people with a learning disability reflect broader systemic issues documented in the general population,^4^ but these may be amplified due to structural inequalities and diagnostic overshadowing (where symptoms are attributed to a person’s learning disability rather than recognised as a separate health condition).

Concerns have been documented relating to end-of-life decision-making for people with a learning disability, including making appropriate DNACPR recommendations. These involve low levels of knowledge about the specific needs of people with a learning disability,^10^ inappropriate assumptions about their quality of life and recognising end-of-life care needs for people with a learning disability.^11^ Previous research indicates barriers to providing good end-of-life care with appropriate DNACPR discussions include difficulties assessing pain or symptom severity in patients with limited communication and a lack of understanding of the Mental Capacity Act.^9^

During the COVID-19 pandemic, discriminatory DNACPR practices disproportionately impacted people with a learning disability, which may have contributed to individuals with learning disability having a COVID-19 death rate >4 times higher than the general population.^12^ National concerns were raised about the use of ‘blanket’ DNACPR decisions, where recommendations were applied broadly, without individual assessment or consultation with individuals or families.^13 14^

In response to these concerns, the National Health Service (NHS) in England issued letters in 2020, 2021 and 2023, reminding healthcare providers that DNACPR orders must not be based solely on the presence of a learning disability or autism.^15–17^

Despite these efforts, problematic clinical reasoning in DNACPR decisions persists in England. A 2024 report by the Parliamentary and Health Service Ombudsman, which examined DNACPR use in the general population, identified ongoing issues including ableism and ageism. The report recommended several improvements including staff training, accessible communication for conversations around end-of-life decisions, updated guidance and improved record-keeping.^18^

### Following DNACPR recommendations

Concerns persist around the implementation of DNACPR recommendations for people with a learning disability. ‘Learning from lives and deaths - people with a learning disability and autistic people’ (LeDeR, an NHS England improvement initiative aimed to reduce health inequalities) annual reports indicate a rise in issues related to DNACPR processes during the pandemic. Almost two-thirds of deaths of people with a learning disability involve DNACPRs, yet the recommendations were assessed to have not been followed correctly in a significant proportion of cases.^19 20^

There is limited research specifically on why DNACPRs are not followed for people with a learning disability, though studies conducted with general population samples suggest factors like clinician override, poor communication and lack of clarity about the requirements of DNACPR may contribute.^21–23^ Misunderstanding of DNACPR meaning among healthcare professionals, such as believing it extends to the withdrawal of other treatments or interventions, further complicates practice.^24^

This paper aims to explore the circumstances in which DNACPR recommendations were not followed, resulting in CPR being administered to people with a learning disability who subsequently died, through thematic analysis of relevant cases in England.

## Methods

### Data source

This work uses data from ‘Learning from lives and deaths – people with a learning disability and autistic people’, a national mortality review in England which aims to address health inequalities.

Deaths are reported to LeDeR via a website and reviewed to confirm the person had a learning disability and registered as a health services user in England. A trained reviewer completes a structured review of the death, including consultation with those involved in care and a review of records. Reviews include multiple-choice and free-text narratives. For this study, particular attention was given to the free-text responses, especially where reviewers commented on issues related to DNACPR. All reviews are anonymised prior to data being transferred to the academic team for analysis. Although reviewers follow guidance, the free-text information written in reviews is based on an individual reviewers’ interpretation of the person’s care, treatment and the circumstances surrounding the death,

### Sample selection

The sample was drawn from a database containing all LeDeR reviews of people with a learning disability who died between 2020 and 2022 and had responses to the question: ‘Was the DNACPR documentation correctly completed and followed’ (n=3600). Online supplemental table 1 presents the potential multiple-choice responses to this question.

10.1136/bmjopen-2025-107222.supp1 Supplementary data

### Sample with DNACPR recommendations not followed correctly

66 reviews of the 3600 reviews were identified from response categories indicating that DNACPR recommendations were in place but some aspect of the DNACPR (either making the decision or carrying out the recommendation) had not been followed correctly. These included cases where DNACPR documentation was recorded as ‘correctly completed but not followed’ (n=42) and ‘neither completed nor followed correctly’ (n=24). Starting from the most recent date of death, reviews were read in their entirety by one of the authors (CR) to ascertain the availability of information in the review about DNACPR.

Following full review, cases were excluded where:

1. DNACPR recommendations themselves were followed despite issues in the decision-making process (n=25);
2. There was no further detail in the free text within the review of a DNACPR-related issue (n=15); or
3. There was insufficient detail in the review to analyse the circumstances surrounding DNACPR (n=5).

21 reviews were included for qualitative analysis

### Descriptive statistics

Descriptive statistics were gathered from LeDeR reviews. Sex, age at death, ethnicity and place of death are reported to give context to the qualitative findings.

### Methodology

This study is underpinned by an interpretivist approach, recognising that meaning is constructed through subjective interpretation of experiences and accounts. Following the approach to inductive thematic analysis^25^ six steps were undertaken to thematically analyse the reviews of deaths by three researchers (CR, AW and ND). Free-text fields (see table 1) were extracted from the master file containing all LeDeR review data, and entered into a separate Microsoft Excel spreadsheet. The authors followed the Standards for Reporting Qualitative Research (SRQR) guidelines.^26^

**Table 1:** Parts of the LeDeR initial review used by researchers to search for information about DNACPR adherence for the qualitative thematic analysis

Review question/section	Information included here
Summary of discussion with GP and/or review of GP notes	Free-text box where reviewers are asked to include key events of care and comment on aspects of care from the GP notes they feel are important.
Conversation with another person	This is a free-text box summary of a discussion with another person and/or clinician involved in the care of the person who died.
Pen portrait	The pen portrait is a free-text box where reviewers summarise the person themselves, their preferences, important people to them and important life events.
DNACPR comments ‘Please add any comments about the DNACPR recommendation’	Free-text box where reviewers can add comments about DNACPR.
Issue, concern or potential problem (initial review) Learning and actions: issues (focused review)	Free-text box where reviewers extract learning from the review. As above.

DNACPR, Do Not Attempt Cardiopulmonary Resuscitation; GP, general practitioner; LeDeR, Learning from Lives and Deaths.

Table 1 shows the parts of the initial review which were read and coded by the researchers for information about issues with DNACPR recommendations being followed. All parts of the review considered by the researchers were free-text boxes.

#### Positionality

The research team have expertise in learning disability and end-of-life care. A reflexive approach was adopted throughout the study, with researchers engaging in ongoing critical reflection on how their backgrounds, assumptions and experiences may have influenced the research process, including interpretation of the data.

#### Thematic analysis

##### Data familiarisation

Both researchers independently read all reviews fully to immerse themselves in the data. This involved actively reading, reflecting and re-reading the reviews several times.

##### Generating codes

A flexible and iterative process was undertaken. Initial codes were generated from the data, primarily completed by one researcher (CR). Reflective discussions were then carried out with the second researcher (AW).

##### Developing themes

Initial themes were reviewed and compared with identified relationships, overlaps and inconsistencies. Iterative coding using mind maps on paper refined codes into themes until saturation was reached.

##### Refining mature themes

Mind maps were used to group, ungroup and regroup early themes into mature themes. The researchers agreed on names and descriptions that best captured each theme.

##### Producing the analysis

The final analysis is presented in the results section below. Any names included in the quotes have been removed to prevent identification.

##### Independent review of analysis

For further validation, an additional author (ND) independently reviewed the coded dataset and thematic structure. Where coding differed, the thematic structure was revised through iterative discussion.

### Patient and public involvement

The LeDeR programme works with people with a learning disability through the Staying Alive and Well co-production group, which reviews findings, contributes lived-experience perspectives and helps disseminate the work, including producing accessible reports.

## Results

### Demographics

The demographics of the 21 adults with a learning disability whose deaths were reviewed by LeDeR and judged by reviewers to have had DNACPR followed incorrectly are shown in table 2. The mean age at death of the sample was 64.6 (SD=9.5) years.

**Table 2:** Descriptive statistics of the demographics of the sample of 21 adults with a learning disability whose deaths were reviewed by LeDeR and judged by reviewers to have had DNACPR followed incorrectly. Numbers below five have been suppressed with an asterisk to preserve anonymity

Demographic	N (%)
Sex	
Male	8 (38)
Female	13 (62)
Ethnicity	
White	20 (95)
Place of death	
Hospital	8 (38)
Usual residence	11 (52)
Other	*
Year of death	
2021	8 (38)
2022	13 (62)

DNACPR, Do Not Attempt Cardiopulmonary Resuscitation; LeDeR, Learning from Lives and Deaths.

### Overview of thematic analysis

Three main themes highlight key challenges in how DNACPR recommendations are followed in practice:

- Responding to an emergency: how people respond in urgent, high-pressure situations.
- Knowledge and awareness of DNACPR: whether staff are aware of and/or understand the scope of DNACPR recommendations.
- Making and enacting DNACPR decisions: how DNACPR recommendations are made and recorded, and the decisions to enact them.

Across all three themes, two cross-cutting factors are identified: breakdowns in information sharing and unclear or inaccessible documentation. Cross-cutting factors shaped how DNACPR recommendations were understood, communicated and enacted in practice. These factors contribute to conditions of uncertainty across the three themes and culminate in a default to life-preserving action: the initiation of CPR despite an active DNACPR recommendation being in place. These factors were identified across multiple cases and are embedded within the themes presented below.

**Figure 1:**
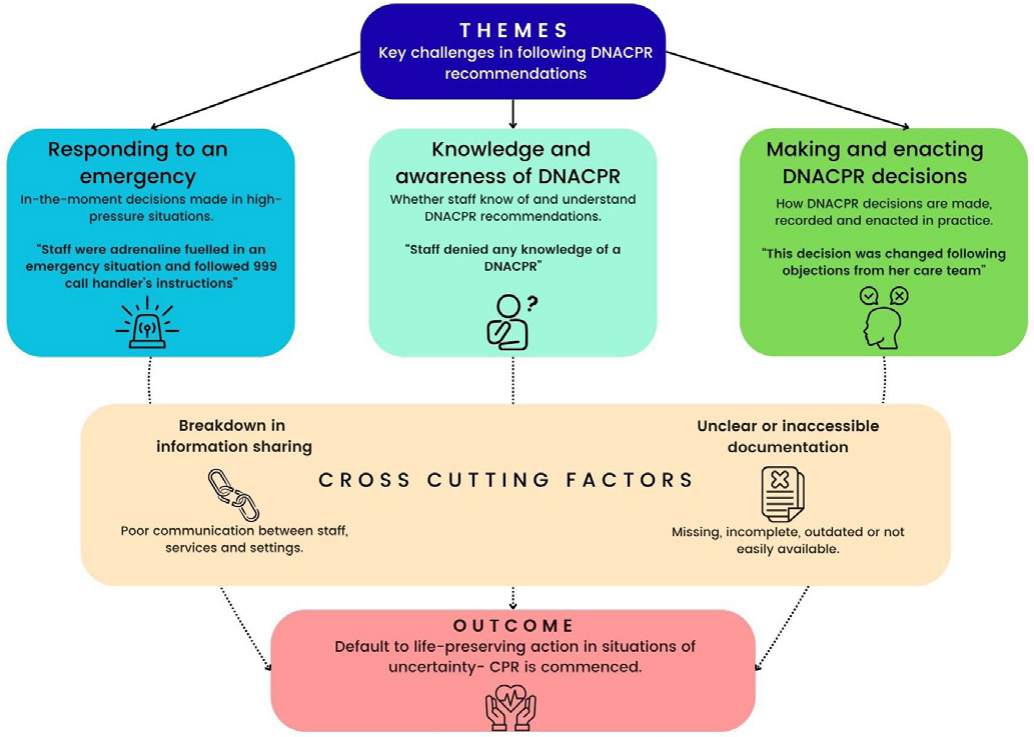
Thematic map. CPR, cardiopulmonary resuscitation; DNACPR, Do Not Attempt Cardiopulmonary Resuscitation.

A thematic map of the data is shown in figure 1.

### Themes

#### Responding to an emergency

This theme represents the challenges faced when responding to an emergency. Often emergencies were unexpected and took paid carers or health professionals by surprise. Several of the reviews commented on the sudden nature of the person’s collapse, and how those around them responded instinctively and called for help from emergency services. It was emergency services involvement that sometimes triggered CPR, as those attending to the collapse would follow instructions from the call handlers.

Staff were adrenaline fuelled in an emergency situation and followed 999 call handler’s instructions. - Reviewer 1

It was a terrible shock to everyone when he was found unresponsive. CPR was carried out by staff until the paramedics came. - Reviewer 21

Here, the cross-cutting factor of breakdowns in information sharing was particularly evident. DNACPR recommendations were not always communicated to emergency services, meaning that responders acted without knowledge of the recommendation.

It is reported by [relative] that she was upset that resuscitation was being commenced as the DNACPR was not known to the ambulance service. - Reviewer 19

In other cases, the cross-cutting factor of unclear or inaccessible documentation shaped how the emergency unfolded. Staff were aware of a DNACPR recommendation but were unable to locate the relevant documentation in time. The challenges of locating documentation were particularly evident in residential home settings, where records may not be as easily accessible as in clinical environments.

Carers called emergency services and carried out CPR for approximately 10 minutes until they located the TEP and then ceased. - Reviewer 9

CPR attempts were sometimes ceased once paramedics arrived and could access medical records and verify a person’s DNACPR status. This information was not always readily accessible in residential or non-medical settings, leading to confusion and distress about how to proceed.

After [the] paramedic obtained medical advice they found [the] records stated [the person] was not for resuscitation and attempts stopped. - Reviewer 5

#### Knowledge and awareness of DNACPR

The second theme is around the knowledge and awareness of DNACPR among those involved in caring for the person with a learning disability. Issues around knowledge and awareness of DNACPR were on two distinct levels.

First, there were concerns about people being unaware of a DNACPR recommendation being in place at all. Being unaware of an active, valid DNACPR meant those attending to the collapsed patient proceeded in the natural and appropriate way by commencing CPR.

Staff denied any knowledge of a DNACPR. - Reviewer 17

In other cases, this lack of awareness was compounded by the cross-cutting factor of unclear or inaccessible documentation. Systems intended to alert staff to DNACPR status were not effective. Documentation may have been present but was not clearly visible or accessible.

CPR was commenced as records viewed by staff did not reflect an alert that she had a DNACPR in place, during the efforts to resuscitate her it was noted that the alert was now evident on her records so CPR stopped. - Reviewer 18

Second, in some cases, individuals caring for the person with a learning disability struggled to understand what a DNACPR recommendation means in practice. When faced with a collapse, staff called emergency services and followed instructions, sometimes without confidence about how to apply a DNACPR recommendation. Reviewers commented on the distress this confusion caused for the people involved in the situation, and some examples highlighted the ‘panic’ and ‘upset’ felt, particularly by care home staff.

CPR was commenced following instruction from 999. There was a ReSPECT form in place however there was a lot of confusion around this. It had not been clearly communicated with [relative] or care team. - Reviewer 20

In this case, the cross-cutting factor of breakdowns in information sharing was evident—the DNACPR recommendation had not been communicated with members of the person’s care team.

#### Making and enacting DNACPR decisions

The final theme relates to issues with making and enacting decisions about DNACPR, including concerns or disagreements about how the recommendation was made and whether they should be followed in practice.

Several cases highlighted disagreements and uncertainty about the appropriateness of a DNACPR recommendation. One reviewer described a situation in which a person’s family had agreed with a DNACPR recommendation, but the recommendation was changed following objections from the care team. This breakdown in information sharing led to some confusion about the validity of the recommendation.

‘The DNACPR completed at that time in discussion with her family advised no treatment due to her poor quality of life. This decision was changed following objections from her care team. - Reviewer 15

In another example, the person collapsed due to an accident—not the progression of their condition—which prompted a reversal of an existing DNACPR recommendation. Uncertainty about how to proceed confused care home staff, who had to escalate the decision about enacting a DNACPR recommendation.

It was recognised by the staff who found [Name] that she had a DNACPR, they phoned the on-call director who advised them to start CPR given the circumstances she had been found in. - Reviewer 15

In other cases, issues centred around considering the wishes and feelings of the person with a learning disability and whether these had been considered in deciding to make a recommendation.

It was also noted that the Care Home paperwork (Covid-19 Hospital Summary) recorded that the patient said ‘Doctors have previously put DNAR in place without asking me. I have made it clear that I want to be resuscitated’. - Reviewer 1.

The cross-cutting factor of unclear or inaccessible documentation was particularly prominent in this theme. In several cases, DNACPR documentation was incomplete, unsigned, or lacked clarity about how the decision was reached or who was consulted.

It does not appear that the GP signed off [Name]’s emergency health care plan or advance care plans, nor did they attend meetings where these were formulated. - Reviewer 12

This review also exemplified the cross-cutting factor of breakdowns in information sharing, as the appropriate professionals were not involved or consulted in the decision-making process of making the recommendation.

In other cases, the validity of DNACPR recommendations was questioned due to documentation being perceived as outdated or no longer applicable.

Ambulance log states DNACPR in place staff stated, ‘It is not valid as it is old’. Recorded that invalid DNACPR had been viewed. - Reviewer 13

Where documentation was incomplete, unclear or contested, professionals were required to make rapid decisions without confidence in the validity of the recommendation. Uncertainty about whether the decision-making process had been followed appropriately (such as whether the right people had been consulted or whether the recommendation was current) contributed directly to non-compliance, with CPR initiated.

## Discussion

This paper examined cases where DNACPR recommendations for people with a learning disability were not followed, resulting in CPR being administered. Through qualitative thematic analysis of reviews of deaths, the study identified three themes: responding to an emergency, knowledge and awareness of DNACPR and decision-making processes. Across these themes, two cross-cutting factors were identified: breakdowns in information sharing and unclear or inaccessible documentation. These factors created uncertainty about the presence, validity or applicability of DNACPR recommendations in practice, contributing to a default to life-preserving action and the initiation of CPR despite an active DNACPR recommendation being in place.

Breakdowns in information sharing and documentation issues were central to these failures, shaping how DNACPR recommendations were interpreted and acted on in practice. In emergencies where DNACPR documentation was unclear, inaccessible or perceived as invalid, professionals often defaulted to life-saving interventions. This reflects the ethical dilemma those responding to a cardiac arrest face, balancing the need to adhere to documented end-of-life decisions and the professional duty to attempt life-saving treatment. While challenges in locating and accessing DNACPR documentation in an emergency may affect any patient, people with a learning disability may be more vulnerable to these situations. People with a learning disability are more likely to live in residential or supported living settings and receive care from a range of professionals across multiple services,^19^ where medical documentation may not be as readily accessible, and those present may not have clinical training.

These findings are not unique to people with a learning disability. National reviews and empirical studies have shown that DNACPR recommendations are particularly vulnerable to non-compliance at the point of implementation, particularly during acute deterioration of health and out-of-hours care in emergency situations.^4 6–27^ However, people with a learning disability are less likely to be directly involved in end-of-life discussions, more likely to rely on proxy decision-makers, and more vulnerable to assumptions about quality of life and prognosis.^7 28^ Our findings suggest that these existing inequities may amplify the risks associated with unclear communication and documentation. To improve practice around DNACPR recommendations for people with a learning disability, facilitating better communication between all those involved in care, alongside accessible and readily available documentation, can reduce uncertainty in emergency situations and support DNACPR recommendations being followed as intended.

Previous research has indicated that misunderstanding of DNACPR recommendations is common among nursing professionals working in elderly adult care.^24^ Our findings relating to the second theme were consistent with this but extend to social care staff such as those working in care homes. Understanding and knowledge issues are potentially key barriers to both following DNACPR recommendations and ensuring recommendations are appropriate. More work is needed to explore how health and social care professionals understand DNACPR recommendations and how this may affect the correct implementation of procedures.

Despite its relevance, the Mental Capacity Act^9^ was rarely mentioned in reviewer comments, perhaps due to the limitations in the structure of the reviews themselves, which do not prompt formal reflection on the Mental Capacity Act. Failure to embed Mental Capacity Act principles may not only lead to inappropriate care but also reinforce existing structural inequalities for people with a learning disability, particularly where assumptions are made about their ability to engage in these sensitive discussions at the end of life. This may further contribute to uncertainty in decision-making processes, particularly where roles and responsibilities of those involved in decision-making are unclear.

### Strengths and limitations

A strength of this paper is that it provides rich qualitative findings which give insight into issues surrounding cases in which DNACPR recommendations are not followed for people with a learning disability, leading to CPR taking place. A rigorous qualitative approach was undertaken which identified key areas of improvement for practice around DNACPR in this population.

As a retrospective analysis of secondary data, findings are limited by the details that reviewers chose to share (or had available) about the events surrounding a death. Researchers did not have access to the DNACPR documentation and did not have firsthand accounts of the events surrounding situations in which CPR was considered. Although reviewers are experienced professionals who receive specialist training, accounts may be subject to bias. The data may not represent all groups, with ethnic minority communities potentially under-represented. Findings should be interpreted as highlighting challenges in implementing DNACPR recommendations, rather than the appropriateness or quality of the decision-making around the original DNACPR recommendation.

## Conclusion

This study identifies factors surrounding cases where DNACPR recommendations are not followed. Unclear or inaccessible documentation and uncertainty about how decisions are made in making and enacting DNACPR recommendations contributed to recommendations not being followed as intended. Addressing these issues is important for people with a learning disability, who often receive care across multiple services and may require additional support to ensure their wishes are heard and understood.

There is a need for targeted training for staff who may encounter situations in which a DNACPR recommendation will be actioned, including care home staff, support workers and emergency responders. Training should focus on understanding what a DNACPR recommendation means, how these recommendations are recorded and reviewed and where to find the relevant documentation alerting professionals to a person’s DNACPR status. Improving knowledge around DNACPR for among health and social care staff will support clearer communication across services involved in the care of people with a learning disability.

Health and social care professionals involved in DNACPR decisions should ensure that the principles of the Mental Capacity Act^9^ are embedded in clinical practice, with meaningful and accessible involvement of the person. Best interest decisions should be well-recorded and involve all relevant parties, including family members, carers and all professionals involved in care.

Future research should assess the extent to which DNACPR recommendations are in line with recent recommendations^18^ and examine how the Mental Capacity Act^9^ is being used in the DNACPR decision-making process for people with a learning disability.

## Supplementary Material

Reviewer comments

Author's manuscript

## Data Availability

No data are available.
