# Supplementary material for: ‘An adrenaline-fueled emergency’: a qualitative thematic analysis of reviews of deaths in England where DNACPR (Do Not Attempt Cardiopulmonary Resuscitation) recommendations are not implemented for people with a learning disability
Source: BMJ Open. 2026 Jun 25;16(6):e107222. doi: 10.1136/bmjopen-2025-107222 (PMC13358332; doi:10.1136/bmjopen-2025-107222)
Supplement: Supplementary data [file bmjopen-16-6-s001.pdf]

Supplementary Table 1: Reviewer judgment of whether DNACPR documentation was correctly completed or followed for reviews of deaths in 2020 to 2022 of people with a learning disability with a DNACPR recommendation in place.

Was the DNACPR documentation correctly completed and followed?	2020	2021	2022	Total
**Correctly Completed and Followed**	52.0% (n = 92)	60.9% (n = 1,144)	63.3% (n = 979)	61.5% (n = 2,215)
**Correctly Completed but Was Not Followed**	*	1.1% (n = 21)	1.2% (n = 19)	*
**Incorrectly Completed and Followed**	11.9% (n = 21)	6.2% (n = 116)	7.1% (n = 109)	6.8% (n = 246)
**Neither Completed nor Followed Correctly**	*	0.6% (n = 11)	0.8% (n = 13)	*
**Don't know**	35.0% (n = 62)	31.2% (n = 585)	27.6% (n = 426)	29.8% (n = 1,073)
**Total**	177	1,877	1,546	3,600
