# Supplementary material for: ‘An adrenaline-fueled emergency’: a qualitative thematic analysis of reviews of deaths in England where DNACPR (Do Not Attempt Cardiopulmonary Resuscitation) recommendations are not implemented for people with a learning disability
Source: BMJ Open. 2026 Jun 25;16(6):e107222. doi: 10.1136/bmjopen-2025-107222 (PMC13358332; doi:10.1136/bmjopen-2025-107222)
Supplement: Reviewer comments [file bmjopen-2025-107222.reviewer_comments.pdf]

PEER REVIEW HISTORY

BMJ Open publishes all reviews undertaken for accepted manuscripts. Reviewers are asked to complete a checklist review form and are provided with free text boxes to elaborate on their assessment. These free text comments are reproduced below.

ARTICLE DETAILS

Title (Provisional)

"An adrenaline-fueled emergency": a qualitative thematic analysis of reviews of deaths in England where DNACPR (Do Not Attempt Cardiopulmonary Resuscitation) recommendations are not implemented for people with a learning disability

Authors

Roberts, Christina; Ditzel, Nicola; White, Adam; Sheehan, Rory; Strydom, André; Ruck-Keene, Alex; Chauhan, Umesh

---

VERSION 1 - REVIEW

**Reviewer**	**1**
**Name**	**Doody, Owen**
**Affiliation**	**University of Limerick Faculty of Education and Health Sciences, Nursing and Midwifery**
**Date**	**19-Sep-2025**
**COI**	**None**

Introduction - While the introduction explains DNACPR, its purpose, and legal context, the central focus of the paper is not clear. The introduction would benefit from identifying the focus of the paper and why this topic is important.

DNACPR recommendation decision-making for people with a learning disability - This section would benefit from a greater distinction between the challenges inherent in DNACPR for people with learning disabilities and the broader systemic issues, such as the "blanket approach" during COVID-19 to guide the reader.

Following DNACPR recommendations - this section highlights the failure to follow DNACPR recommendations for people with LD. However, greater explanation of how these issues affect people with LD would enhance. You start with an emphasize DNACPR failures but could emphasise the unique challenges for people with LD. Also mention LeDeR reports but no explaination of the LeDeR program for readers not familiar with this initiative.

Method - justify why using LeDeR data as the primary source is appropriate and why LeDeR data provides unique insights into DNACPR practices. Greater clarity as to why "Was the DNACPR documentation correctly completed and followed?" was used as a proxy for whether the DNACPR

recommendation was not followed. Sampling starting with the most recent reviews and working backward ? potential biases (could the more recent cases have been reviewed more thoroughly or documented differently compared to earlier cases). Thematic analysis, 2 researchers independently reviewed the data, how was consensus formed or disagreements resolved (how differences in coding or interpretation were handled). No description of how saturation was defined or measured. For other researchers to replicate greater detail of the process of data analysis is needed. Descriptive statistics used and useful if any patterns were observed related to DNACPR adherence and demographics. Although ethical approval is implied due to the use of data from the LeDeR program, there is no explicit mention of ethical considerations given the sensitive nature of the data. There should be an acknowledge of potential biases in using retrospective data.
Findings/Discussion - The themes, "Communication" and "Instinct to protect life," while compelling, do they oversimplify the complexities i.e. mask the emotional, ethical, and procedural difficulties involved in decisions around resuscitation. The themes (Responding to an emergency, Knowledge and awareness of DNACPR, Documenting the recommendation, and The decision-making process) don't appear to fully address deeper structural issues, such as inequalities in healthcare for people with LD or the adequacy of legal frameworks governing end-of-life care. The themes of "Documenting the recommendation" and "Communication" frequently overlap.
While the discussion touches on the need for better communication regarding DNACPR recommendations, it does not explore specific strategies for improving this communication. The mention of the Mental Capacity Act (MCA) is important, but the discussion could elaborate on how its principles could be practically integrated into DNACPR conversations. The discussion could better articulate the implications of these findings for policy and practice. For example, emphasising how improved understanding of DNACPR could influence training requirements, organisational policies, or quality assurance measures in healthcare settings. The instinct to protect life is an important theme, but the discussion could explore the ethical dilemmas faced by caregivers more thoroughly. Incomplete or unavailable documentation could discuss the training gaps that may be contributing to poor communication between health professionals.
No conclusion.

**Reviewer**	**2**
**Name**	**Slowther, Anne-Marie**
**Affiliation**	**University of Warwick, Warwick Medical School**
**Date**	**02-Oct-2025**
**COI**	**None**

This paper seeks to address an important issue around implementation of DNACPR recommendations for people with a learning disability. As the authors describe in their introduction, there is evidence of inequity in how DNACPR and other advance care planning conversations are carried out, disadvantaging people with a learning disability. However, it is not

clear from this paper whether similar inequities exist in the carrying out of DNACPR recommendations at the time of a cardiac arrest. Many of the problems identified in the themes presented in the paper are unlikely to be unique to cases where the patient has a learning disability. The authors have not really reflected on whether having a learning disability makes a substantive difference to whether a DNACPR recommendation is followed, which as I understand it, is the focus of this paper. The paper provides useful information on some of the reasons why DNACPR recommendations may not be followed in practice which may be generalisable to all patients. I have made some specific comments on sections of the paper below.

Title I think the title is slightly misleading. The study is not really a qualitative exploration of the actual instances where DNACPR recommendations were not followed, but a qualitative analysis of reviews of cases where DNCAPR recommendations were not followed. This may be slightly pedantic but I think it is important to flag in the title that this is an analysis of secondary rather than primary data.

Abstract
The abstract is well structured. The conclusions do not specifically refer to people with a learning disability and seem generally applicable to all cases where DNACPR recommendations are in place. This reflects my general comment about the paper mentioned above. In the findings section the authors identify one theme as 'the decision-making process'. This could be interpreted as the decision to commence CPR/not to follow a DNACPR recommendation. I think what the authors mean (looking at the main paper) is the process of making a DNACPR recommendation.

Introduction
Page 4 line 3. The reference to low rates of success from CPR in older adults suggests that DNACPR recommendations are only for older adults. CPR overall has a low success rate. The recommendation not to attempt CPR relates to the chance of success in a particular individual (informed by population data).
Page 4 line 8. DNACPR recommendations are always made by health care professionals, even if part of an advance planning discussion. An individual person may make an advance refusal of treatment specifying CPR (as the authors describe later in the paper).
Page 4 line 35 case law and clinical guidance does state that when a person lacks capacity decisions for them should be made in consultation with a legal proxy or those close to them, but this is reflecting the requirement of the Mental Capacity Act. It Is not clear from the text that this is the case. It reads as if the MCA is additional to case law and guidance.
Page 4 line 44. This paragraph clearly discusses the challenges of treatment (including anticipatory treatment) decision-making with people with a learning disability. However, the focus of this paper is on following recommendations once made rather than the process of making those recommendations. Similarly, the following paragraphs describe concerns about use of DNACPR recommendations during COVID which primarily relate to the recording of DNCPR recommendations without proper consultation. This is very important, but it is not clear how it links

to decisions not to follow a DNACPR recommendation (unless mistrust of the validity of the recommendation given this historical concern leads first responders to question such a recommendation more in a person with a learning disability). I see that this section highlights the existing inequity for people with a learning disability around the process of making these recommendations, but it is important to highlight that this part of the process is not the subject of this particular study.

Page 5 section on not following DNACPR recommendations.

The authors note that there is little evidence on why DNACPR recommendations may not be followed for people with a learning disability but that there is evidence on this issue in the general population. I am surprised that in this section the authors do not refer to the NCEPOD review in 2012 of patients who underwent CPR following an in hospital cardiac arrest. The review found that of 573 patients who received CPR 52 had a DNACPR recommendation in place. https://www.ncepod.org.uk/2012report1/downloads/CAP_summary.pdf. Also there is no reference to the study by Perkins et al in 2016 of the use of DNACPR across health care settings in the UK. The study conclusion was that 'There is evidence of variation and suboptimal practice in relation to DNACPR decisions across health-care settings. There were deficiencies in considering, discussing and implementing the decision, as well as unintended consequences of DNACPR decisions being made on other aspects of patient care.'
https://www.ncbi.nlm.nih.gov/books/NBK355498/ .

Given the lack of evidence around CPR decision making for people with a learning disability I find it interesting that the authors have chosen to focus on situations where recommendations are not followed rather than the process of making recommendations, where it seems more likely that inequity will be found.

Method

Sample selection flow chart: I find this flow chart confusing and therefore may have misunderstood it. I do not see how the final sample of 21 was arrived at.

- 66 reviews with DNACPR documentation either not correctly completed or followed
- 25 excluded because the DNACPR recommendation was followed
- 15 excluded because there was no evidence of any issue with the DNACR process (I assume this means that although a problem was indicated there were no data about what the problem was).
- Therefore 40 were excluded from the original 66, leaving 26 records. Thus, I do not see how the final sample was 21.

Page 8 line 43 The reference for inductive thematic analysis is rather old. Braun and Clarke have revised their thinking on thematic analysis with different interpretations and more nuanced definitions. The authors could consider using a more recent reference eg Braun V, Clarke V. One size fits all? What counts as quality practice in (reflexive) thematic analysis? Qualitative research in psychology. 2021 Jul 3;18(3):328-52.

Results

The description of the process of thematic analysis (section 3.2) is methods and not results.

Diagram describing the themes identified. This is a nice visual representation of themes. It would be worth clarifying whether the theme of decision-making process relates to the decision to make a CPR recommendation, a decision to commence CPR, or both as this is not clear from the codes.
Page 14 line 10. I have a recurring problem with the lack of clarity regarding use of the term decision making process. In this communication theme I think what is being described here is that if the discussion leading to a CPR recommendation has not been transparent or the presence and nature of the recommendation not communicated to the family or care team, then there is doubt about the validity and conflict about the appropriateness of the recommendation. However, communication is also important in the process of making a decision about whether to follow a CPR or DNACPR recommendation.
Page 14 line 38. This paragraph is referring to how an instinct to preserve life may influence discussions about anticipatory decision making such as DNACPR, but it Is not clear how this is relevant to the decision to follow or not follow a clear DNACPR recommendation. Unless the person involved in the resuscitation decision at the time of cardiac arrest was party to the discussion leading to the recommendation, they would not be aware that agreement had not been reached.
It would strengthen the paper if there were some illustrative quotes to support the overarching theme of 'instinct to protect life'
Page 16 line 29 This quote was used earlier in the paper. A different quote would be helpful.
Page 17 line 48 identifying name has been included in the quote. This needs to be removed.
Page 17 line 36 This is a really interesting example and quote illuminating challenges and disagreements over what recommendation should be made. However, I am not clear how a changed recommendation impacted on whether the recommendation was followed. It would be helpful for this chain of reasoning to be expanded on.
Page 17 line 51. Again, this is an interesting quote, and it would be helpful to have some expansion of how the care home paperwork fed into the decision not to follow the DNACR recommendation. Was this because the care home records were being consulted during the arrest? Or that a member of staff recalled that the paten had said this. Or is the reviewer just noting that there had been conflict over the recommendation. There is a distinction to be made between incomplete documentation because of conflict during the process of making a recommendation (so not a valid recommendation) and a valid recommendation from a procedural point of view (properly completed) but additional evidence or input that challenges the recommendation at the time of cardiac arrest. While it may not be possible to differentiate from reviewer comments the authors could perhaps in their interpretation of the data consider these different scenarios.

Discussion
Page 18 line 25 I wholeheartedly agree with the authors that the process of discussion around emergency care treatment planning including CPR decisions needs to improve for people with a learning disability, including those who have capacity and those who lack capacity. However, it Is not clear to me how this relates to improving practice in terms of following documented DNACPR recommendations. It could be that if the process of developing the recommendation is more sound

clinicians implementing them may feel more confident in them. But as it is often the case that the clinician responding in an emergency has no knowledge of the quality of the discussion that led to the recommendation, it is difficult to make this link. There appears to be a step missing in the line of argument in this section of the discussion.
The discussion would be strengthened by placing this study's findings in the wider context of compliance with DNACPR recommendations in the general population. As it stands, I am not clear in what way this element of DNACPR decision making (following a recommendation) is different for people with a learning disability.

**Reviewer**	**3**
**Name**	**Michalowski, Sabine**
**Affiliation**	
**Date**	**09-Oct-2025**
**COI**	**None**

This is a really interesting paper which provides some insight into situations where DNACPR decisions for persons with learning disabilities are not followed and result in CPR being administered. My main issue with the paper is that it does not provide a clear comparison with DNACPR decisions and their disregard in respect of persons without learning disabilities, which makes it really difficult to understand to what extent the learning disability had any impact on the outcome, ie the administration of CPR despite a DNACPR being in place. This means that it is difficult to understand what to learn from the study and to what extent any of the results have a causative link with the learning disability or whether the study rather shows that the same problems that exist with DNACPR decisions in general, around communication and the instinct to preserve life, ambiguities when interpreting the decisions etc., simply equally apply to persons with learning disabilities.

While not suggesting that a comparative study would be necessary here, I think the article would benefit from clarifying these points and providing an explanation of why the particular issues identified as results of the study are specific to DNACPR decisions for persons with learning disabilities.

VERSION 1 - AUTHOR RESPONSE

Reviewer Comment	Author Response	Corresponding changes and location
Introduction - While the introduction explains DNACPR, its purpose, and legal context, the central focus of the paper is not clear. The introduction would benefit from identifying the	The introduction has been tightened and we believe the changes made incorporating other comments address this	

focus of the paper and why this topic is important.	general remark about clarifying the focus of the paper.	
DNACPR recommendation decision-making for people with a learning disability - This section would benefit from a greater distinction between the challenges inherent in DNACPR for people with learning disabilities and the broader systemic issues, such as the "blanket approach" during COVID-19 to guide the reader.	We have amended this section to focus more specifically on learning disabilities. The 'blanket approach' during COVID-19 did disproportionately affect people with learning disabilities living in care home setting so we have retained this.	This section of the introduction has been amended.
Following DNACPR recommendations - this section highlights the failure to follow DNACPR recommendations for people with LD. However, greater explanation of how these issues affect people with LD would enhance. You start with an emphasize DNACPR failures but could emphasise the unique challenges for people with LD.	This has been edited to be more specific of learning disability and their unique challenges.	
Also mention LeDeR reports but no explaination of the LeDeR program for readers not familiar with this initiative.	Added in brackets a small explanation the first time we mention LeDeR (under heading 1.2).	(a National Health Service service improvement program aimed to improve health inequalities)
Method – 1. justify why using LeDeR data as the primary source is appropriate and why LeDeR data provides unique insights into DNACPR practices.<br>2. Greater clarity as to why "Was the DNACPR documentation correctly completed and followed?" was used as a proxy for whether the DNACPR recommendation was not followed.<br>3. Sampling starting with the most recent reviews and working backward ? potential biases (could the more recent cases have been reviewed more thoroughly or documented differently compared to earlier cases).	1. We are not aware of any other dataset with this level of detail available for analysis in relation to the use of DNACPR in people with a learning disability...?<br>2. The question 'Was the DNACPR documentation correctly completed and followed' allows reviewers to provide details as to whether the documentation was 'neither completed nor followed correctly or 'corrected completely but was not followed'.<br>3. It is possible that as reviewers became familiar with responding to this question overtime and may have altered how they completed the form. There is also likely to be variation in different reviewers' responses and interpretation which may also lead to potential biases. (we have	

Commented [CR1]: Address other reviewers' comments about introduction then return to this to see if it has been addressed

Commented [CR2]: Rather than blanket approach – COVID-19 facilitated discriminatory practices approaches to how DNACPRs are put in place, potentially disproportionately affected people with a learning disability

Commented [CR3]: What are the unique challenges - discussion/decision making process which we have emphasized - can rebut and say we are satisfied with our argument and don't feel it needs elaboration

	noted this in the strength and limitations	
Thematic analysis, 2 researchers independently reviewed the data, how was consensus formed or disagreements resolved (how differences in coding or interpretation were handled). No description of how saturation was defined or measured. For other researchers to replicate greater detail of the process of data analysis is needed.	This section has been restructured to emphasis thematic analysis steps taken	Section 2.5 has been restructured and now explains the data analysis steps more thoroughly.
Descriptive statistics used and useful if any patterns were observed related to DNACPR adherence and demographics.<br>1. Although ethical approval is implied due to the use of data from the LeDeR program, there is no explicit mention of ethical considerations given the sensitive nature of the data.<br>2. There should be an acknowledge of potential biases in using retrospective data.	1. discussion of the ethical considerations of the LeDeR mortality data collection is beyond the scope of this paper.<br><br>2. Additional limitations related to biases related to underrepresented groups, recall and observer bias have been included.	We have added to the limitations section of this paper.
Findings/Discussion - The themes, "Communication" and "Instinct to protect life," while compelling, do they oversimplify the complexities i.e. mask the emotional, ethical, and procedural difficulties involved in decisions around resuscitation.	We have changed the write up of these themes to incorporate this comment.	"While the instinct to protect life is compelling, this is perhaps an over simplification of the emotional, ethical, and procedural difficulties involved in resuscitation decisions. It can mask the deep internal conflict people face between doing what feels right in the moment and what aligns with legal or clinical guidance<br>This tension underscores the need for clarity, communication, and support in end-of-life planning, particularly where individuals with a learning disability are concerned."
The themes (Responding to an emergency, Knowledge and awareness of DNACPR, Documenting	Additional section added to reflect on this.	"These cases reveal not only the procedural

Commented [CR4]: add to limitations

the recommendation, and The decision-making process) don't appear to fully address deeper structural issues, such as inequalities in healthcare for people with LD or the adequacy of legal frameworks governing end-of-life care.		challenges of emergency response but also point to deeper structural issues. For people with learning disabilities, inconsistencies in access to healthcare, poor communication across services, and lack of training for frontline responders can compound the risk of inappropriate or delayed care.”
The themes of "Documenting the recommendation" and "Communication" frequently overlap.	We acknowledge that these two themes do overlap, and we believe the diagram produced represents this. Although it is not ideal to have overlapping themes, we were unable to synthesise this analysis in a way that had no overlap.	
While the discussion touches on the need for better communication regarding DNACPR recommendations, it does not explore specific strategies for improving this communication.	We believe the edits for the below comment address this.	
The mention of the Mental Capacity Act (MCA) is important, but the discussion could elaborate on how its principles could be practically integrated into DNACPR conversations.	Added section to the discussion.	Discussion section: Practically, this means that:<br>• **Capacity assessments** must be decision-specific and revisited if circumstances change.<br>• Where individuals **lack capacity**, professionals must still involve them as far as practicable—using accessible

		communication, familiar environments, and input from people who know the individual well.<br>• Best interest decisions must consider the person's past and present wishes, beliefs, and values, and include **all relevant parties** in a meaningful dialogue—not just for legal compliance, but to uphold ethical, person-centred care.<br>• **Advance care planning discussions** should be proactive, not reactive, ensuring that the person's preferences are known and recorded long before a crisis arises.
The discussion could better articulate the implications of these findings for policy and practice. For example, emphasising how improved understanding of DNACPR could influence training requirements, organisational policies, or quality assurance measures in healthcare settings.	We have completely rewritten the discussion and believe this is now addressed.	
The instinct to protect life is an important theme, but the discussion	We acknowledge the ethical dilemmas faced by carers are	We have specifically alluded to the ethical

could explore the ethical dilemmas faced by caregivers more thoroughly.	huge here, and are planning on doing more work focused specifically on this.	dilemmas faced by carers in the first paragraph of the discussion.
Incomplete or unavailable documentation could discuss the training gaps that may be contributing to poor communication between health professionals.	We have referenced a training gap in the conclusion and recommendations.	
Reviewer: 2<br>Dr. Anne-Marie Slowther, University of Warwick<br>Comments to the Author:<br>This paper seeks to address an important issue around implementation of DNACPR recommendations for people with a learning disability. As the authors describe in their introduction, there is evidence of inequity in how DNACPR and other advance care planning conversations are carried out, disadvantaging people with a learning disability. However, it is not clear from this paper whether similar inequities exist in the carrying out of DNACPR recommendations at the time of a cardiac arrest.	There is no / very little evidence in this group before this paper.	
Many of the problems identified in the themes presented in the paper are unlikely to be unique to cases where the patient has a learning disability. The authors have not really reflected on whether having a learning disability makes a substantive difference to whether a DNACPR recommendation is followed, which as I understand it, is the focus of this paper. The paper provides useful information on some of the reasons why DNACPR recommendations may not be followed in practice which may be generalisable to all patients. I have made some specific comments on sections of the paper below.	Thank you for your comment. We hope thtat some of the changes we have made address your concerns.	
Title I think the title is slightly misleading. The study is not really a qualitative exploration of the actual instances where DNACPR recommendations were not followed, but a qualitative analysis of reviews of cases where DNCAPR recommendations were not followed. This may be slightly pedantic but I	We have amended the title to address this comment.	Title now references 'reviews of cases'.

think it is important to flag in the title that this is an analysis of secondary rather than primary data.		
The abstract is well structured. The conclusions do not specifically refer to people with a learning disability and seem generally applicable to all cases where DNACPR recommendations are in place. This reflects my general comment about the paper mentioned above.	We have made the conclusions specifically about people with a learning disability.	
In the findings section the authors identify one theme as 'the decision-making process'. This could be interpreted as the decision to commence CPR/not to follow a DNACPR recommendation. I think what the authors mean (looking at the main paper) is the process of making a DNACPR recommendation.	We actually mean both so have retained the name of this theme.	
Introduction Page 4 line 3. The reference to low rates of success from CPR in older adults suggests that DNACPR recommendations are only for older adults. CPR overall has a low success rate. The recommendation not to attempt CPR relates to the chance of success in a particular individual (informed by population data).	We have amended this.	The sentence no longer reads 'older adults'.
Page 4 line 8. DNACPR recommendations are always made by health care professionals, even if part of an advance planning discussion. An individual person may make an advance refusal of treatment specifying CPR (as the authors describe later in the paper).	We believe the wording of this section is fine.	
Page 4 line 35 case law and clinical guidance does state that when a person lacks capacity decisions for them should be made in consultation with a legal proxy or those close to them, but this is reflecting the requirement of the Mental Capacity Act. It Is not clear from the text that this is the case. It reads as if the MCA is additional to case law and guidance.	An author who is a professor of law has reviewed this section and is satisfied with its accuracy.	
Page 4 line 44. This paragraph clearly discusses the challenges of treatment (including anticipatory treatment)	We still believe this provides important context, as difficulties	

decision-making with people with a learning disability. However, the focus of this paper is on following recommendations once made rather than the process of making those recommendations.	making decisions about putting a DNACPR in place could influence how likely it is to be implemented (for e.g. if carers do not agree with the decision/have not been consulted correctly).	
Similarly, the following paragraphs describe concerns about use of DNACPR recommendations during COVID which primarily relate to the recording of DNCPR recommendations without proper consultation. This is very important, but it is not clear how it links to decisions not to follow a DNACPR recommendation (unless mistrust of the validity of the recommendation given this historical concern leads first responders to question such a recommendation more in a person with a learning disability).	We have restructured this section and hope it now reads better.	
I see that this section highlights the existing inequity for people with a learning disability around the process of making these recommendations, but it is important to highlight that this part of the process is not the subject of this particular study.	We hope the restructured introduction highlights this.	
Page 5 section on not following DNACPR recommendations.<br>The authors note that there is little evidence on why DNACPR recommendations may not be followed for people with a learning disability but that there is evidence on this issue in the general population. I am surprised that in this section the authors do not refer to the NCEPOD review in 2012 of patients who underwent CPR following an in hospital cardiac arrest. The review found that of 573 patients who received CPR 52 had a DNACPR recommendation in place. https://www.ncepod.org.uk/2012report1/downloads/CAP_summary.pdf.	Thank you for notifying us of this reference, we have now incorporated three references to factors that influence why DNACPRs are not followed for a learning disability.	
Also there is no reference to the study by Perkins et al in 2016 of the use of DNACPR across health care settings in the UK. The study conclusion was that 'There is evidence of variation and suboptimal	We thank the reviewers for this reference, though we not been able to access this resource, and have focused more	

practice in relation to DNACPR decisions across health-care settings. There were deficiencies in considering, discussing and implementing the decision, as well as unintended consequences of DNACPR decisions being made on other aspects of patient care.' https://www.ncbi.nlm.nih.gov/books/NBK355498/ .	specifically on evidence around people with learning disabilities.	
Given the lack of evidence around CPR decision making for people with a learning disability I find it interesting that the authors have chosen to focus on situations where recommendations are not followed rather than the process of making recommendations, where it seems more likely that inequity will be found.	We agree that this would be an interesting direction for research, however our data did not provide enough detail about the process of making recommendations, rather it focuses on the circumstances and events leading up to death.	
Sample selection flow chart: I find this flow chart confusing and therefore may have misunderstood it. I do not see how the final sample of 21 was arrived at.<br>• 66 reviews with DNACPR documentation either not correctly completed or followed<br>• 25 excluded because the DNACPR recommendation was followed<br>• 15 excluded because there was no evidence of any issue with the DNACR process (I assume this means that although a problem was indicated there were no data about what the problem was).<br>• Therefore 40 were excluded from the original 66, leaving 26 records. Thus, I do not see how the final sample was 21.	An additional 5 records were excluded due to insufficient data about DNACPR.	Amended flowchart to reflect this.
Page 8 line 43 The reference for inductive thematic analysis is rather old. Braun and Clarke have revised their thinking on thematic analysis with different interpretations and more nuanced definitions. The authors could consider using a more recent reference eg Braun V, Clarke V. One size fits all? What counts as quality practice in (reflexive) thematic analysis? Qualitative research in psychology. 2021 Jul 3;18(3):328-52.	We have updated the reference.	
The description of the process of thematic analysis (section 3.2) is methods and not results.	We have moved this section to methods.	

Commented [CR5]: add detail to methods to explain the 21 were reached because they had adequate descriptive data to be qualitatively analysed

Diagram describing the themes identified. This is a nice visual representation of themes. It would be worth clarifying whether the theme of decision-making process relates to the decision to make a CPR recommendation, a decision to commence CPR, or both as this is not clear from the		
Page 14 line 10. I have a recurring problem with the lack of clarity regarding use of the term decision making process. In this communication theme I think what is being described here is that if the discussion leading to a CPR recommendation has not been transparent or the presence and nature of the recommendation not communicated to the family or care team, then there is doubt about the validity and conflict about the appropriateness of the recommendation. However, communication is also important in the process of making a decision about whether to follow a CPR or DNACPR recommendation.	We take on board your comments about this on the original draft. We have comprehensively reviewed this paper and have tried to add clarity throughout on this.	
Page 14 line 38. This paragraph is referring to how an instinct to preserve life may influence discussions about anticipatory decision making such as DNACPR, but it Is not clear how this is relevant to the decision to follow or not follow a clear DNACPR recommendation. Unless the person involved in the resuscitation decision at the time of cardiac arrest was party to the discussion leading to the recommendation, they would not be aware that agreement had not been reached.<br>It would strengthen the paper if there were some illustrative quotes to support the overarching theme of 'instinct to protect life'	This theme was difficult to evidence using quotes from the reviewer, it was gathered as a general impression from reading the full reviews.	
Page 16 line 29 This quote was used earlier in the paper. A different quote would be helpful.	The quote has been changed.	
Page 17 line 48 identifying name has been included in the quote. This needs to be removed.	This has been removed.	
Page 17 line 36 This is a really interesting example and quote illuminating challenges and	We have amended this section to expand upon the reasoning.	

Commented [CR6]: decision making process about whether DNACPR is appropriate

decision making process about whether a DNACPR should be actioned

Commented [CR7R6]: make clear throughout and change name of theme to the first one

disagreements over what recommendation should be made. However, I am not clear how a changed recommendation impacted on whether the recommendation was followed. It would be helpful for this chain of reasoning to be expanded on.		
Page 17 line 51. Again, this is an interesting quote, and it would be helpful to have some expansion of how the care home paperwork fed into the decision not to follow the DNACR recommendation. Was this because the care home records were being consulted during the arrest? Or that a member of staff recalled that the paten had said this. Or is the reviewer just noting that there had been conflict over the recommendation. There is a distinction to be made between incomplete documentation because of conflict during the process of making a recommendation (so not a valid recommendation) and a valid recommendation from a procedural point of view (properly completed) but additional evidence or input that challenges the recommendation at the time of cardiac arrest. While it may not be possible to differentiate from reviewer comments the authors could perhaps in their interpretation of the data consider these different scenarios.	We acknowledge that this is interesting, sadly this is beyond the scope of data available to us. We hope to complete more work on this though in the future.	
Page 18 line 25 I wholeheartedly agree with the authors that the process of discussion around emergency care treatment planning including CPR decisions needs to improve for people with a learning disability, including those who have capacity and those who lack capacity. However, it Is not clear to me how this relates to improving practice in terms of following documented DNACPR recommendations. It could be that if the process of developing the recommendation is more sound clinicians implementing them may feel more confident in them. But as it is often the case that the clinician responding in an emergency has no knowledge of the quality of the discussion that led to the	The discussion has been streamlined – we agree that this is difficult for clinicians who have not been party to discussions.	

recommendation, it is difficult to make this link. There appears to be a step missing in the line of argument in this section of the discussion.		
The discussion would be strengthened by placing this study's findings in the wider context of compliance with DNACPR recommendations in the general population. As it stands, I am not clear in what way this element of DNACPR decision making (following a recommendation)is different for people with a learning disability.	There is not evidence about this available to us.	
Reviewer: 3<br>Dr. Sabine Michalowski<br>Comments to the Author:<br>This is a really interesting paper which provides some insight into situations where DNACPR decisions for persons with learning disabilities are not followed and result in CPR being administered. My main issue with the paper is that it does not provide a clear comparison with DNACPR decisions and their disregard in respect of persons without learning disabilities, which makes it really difficult to understand to what extent the learning disability had any impact on the outcome, ie the administration of CPR despite a DNACPR being in place. This means that it is difficult to understand what to learn from the study and to what extent any of the results have a causative link with the learning disability or whether the study rather shows that the same problems that exist with DNACPR decisions in general, around communication and the instinct to preserve life, ambiguities when interpreting the decisions etc., simply equally apply to persons with learning disabilities.	The focus here is specifically on DNACPR use in people with a learning disability; we do not agree that a comparison group is required. However, in response to other reviewer's comments we have added additional references and discussion to put our work in a broader context.	
While not suggesting that a comparative study would be necessary here, I think the article would benefit from clarifying these points and providing an explanation of why the particular issues identified as results of the study are specific to DNACPR decisions for persons with learning disabilities.	We have edited the manuscript extensively in response to the other reviewers' comments and believe this is now improved. Our dataset is specifically about people with learning disabilities.	

VERSION 2 - REVIEW

Reviewer 1

Name Doody, Owen

Affiliation University of Limerick Faculty of Education and Health Sciences, Nursing and Midwifery

Date 19-Sep-2025

COI

The authors have tightened the introduction and clarified the focus on learning disabilities in DNACPR decisions. They distinguished learning disability specific challenges from broader systemic issues. Justified use of LeDeR data and updated the thematic analysis reference (Braun & Clarke, 2021) and provide more detail on thematic analysis steps, consensus-building, and limitations.

Remaining weaknesses

Comparison with general population – clarity throughout paper whether issues identified are unique to learning disability or reflect broader DNACPR challenges and discussion still feels underdeveloped in clarifying how learning disability adds distinct challenges. Currently, conclusions could appear generalisable to all DNACPR cases, risking dilution of the paper's unique contribution.

Review process queried whether "decision-making process" referred to making a DNACPR recommendation vs. following it, you claim it means both, which risks conceptual vagueness. A sharper distinction or renaming of themes would strengthen analytic clarity.

Some themes (e.g., "instinct to protect life") remain weakly evidenced, without stronger data excerpts, these themes risk being perceived as speculative.

Policy and practice implications, while rewritten, the discussion could further highlight specific actionable recommendations:

Staff training (emergency responders, care home staff, ambulance services).

Better documentation systems and cross-organisational communication.

Embedding the Mental Capacity Act more firmly in clinical practice.

To reach a publishable standard, the paper would benefit from:

-Sharper framing of contribution

-Explicitly state what is unique about DNACPR for people with learning disabilities (e.g., communication barriers, assumptions about capacity, structural discrimination).

-Contrast with general DNACPR issues so the reader can see the added value.

-Clarify themes

-Separate the "decision-making process" into distinct categories (recommendation-making vs. action at point of arrest).

-Strengthen theme definitions and ensure quotes align clearly.

-Strengthen evidence base

-Use more direct quotes where possible to support themes (even if brief).

-Acknowledge explicitly where themes are interpretive impressions due to data limitations.

-Expand discussion of implications

-Provide clear, actionable recommendations:

Mandatory DNACPR training for staff working with people with learning disabilities.

Standardised protocols for ensuring DNACPR visibility across care settings.

Policy guidance on integrating learning disability specific considerations into DNACPR practice.

Literature integration

Incorporate missing key references on DNACPR in the general population. This would help position findings and address reviewer concerns about generalisability.

Conceptual clarity (uniqueness to learning disability, thematic precision) and stronger policy implications still need work before the paper is likely to be fully publishable.

**Reviewer**	**2**
**Name**	**Slowther, Anne-Marie**
**Affiliation**	**University of Warwick, Warwick Medical School**
**Date**	**02-Oct-2025**
**COI**	

Thank you for the opportunity to review this resubmission. I have read the revised manuscript and responses to reviewers. There are some discrepancies between the responses provided and the

content of the final manuscript, so I am not sure if the response document provided was the final version.

The paper has been substantially re written with the introduction and methods being much clearer than in the first submission. The description of the themes and the additional illustrative quotes also provide a clearer narrative.

However, I still have some major concerns which I do not feel the authors have fully addressed.

My key concern with the first submission, also expressed by reviewer 3 and indirectly by reviewer 1, was that it was unclear how the findings of this study illuminate particular challenges with implementation of DNACPR recommendations in people with a learning disability compared to their implementation in the general population. Clearly the authors cannot directly compare reviews of decisions made in the general population as this was not their data set. However, some reflection on whether it is likely that the issues they have identified as creating difficulties in CPR decision making within their study are common to everyone, or in what way they may be more pertinent for people with a learning disability is required. I do not think that the authors have addressed this adequately in the resubmission.

All of the issues identified in the themes could apply to implementation of DNACPR recommendations for all patients. What I feel is lacking from the analysis and discussion is a reflection on why these issues might be particularly problematic for people with a learning disability, drawing on what is known about challenges and inequities in in end-of-life decision for people with a learning disability, which the authors refer to in their introduction. What is the link between what is known about inequity in end of life decision making for people with a learning disability and what the authors have found in their study?

While acknowledging that this study focusses on lack of compliance with DNACPR recommendations for people with a learning disability I reiterate my previous comment in my original review that the discussion would be strengthened by placing the findings in the wider context of compliance with DNACPR recommendations in the general population. I note the authors' response that they have no evidence for this. I previously suggested referencing the 2012 NCEPOD report and the study by Perkins et al on DNACPR decision making. Both of these would be useful in this context. I am surprised the authors could not identify the Perkins study. It is available in pubmed https://pubmed.ncbi.nlm.nih.gov/27077163/. Also, the authors might want to look at more recent publications on the implementation of ReSPECT in primary care which does not solely focus on CPR recommendations, but the findings are relevant to DNACPR recommendations and reflect some of the challenges to implementation noted in this study.
https://pubmed.ncbi.nlm.nih.gov/39304311/, https://pubmed.ncbi.nlm.nih.gov/39487818/. While

these studies are not directly comparable to this one, they do provide wider context to enrich the discussion and reflection on findings.

Further specific points

Abstract. The conclusion still does not refer to people with a learning disability which is the focus of this study

Introduction

Final sentence second paragraph does not make sense. Clinicians must justify their DNACPR decision if the patient wishes for them?

5th paragraph The reference for the UK survey of residential homes is missing in the text (reference 12) This study did not focus on people with a learning disability but was a more general survey of experiences of DNACPR in care homes during covid. This needs to be clear as it is currently cited in the section on DNACPR for people with a learning disability

Similarly, the Ombudsman report did not refer solely to people with a learning disability

Findings

Overarching theme: instinct to preserve life. I find it difficult to assess a theme that is not supported by any data/quotes.

Theme on emergencies There is no evidence from the data presented that inconsistency in access to health care for the person with a learning disability impacts on challenges related to an emergency scenario. Not having access to the records in this situation is likely to apply to anyone who has a cardiac arrest

Theme on decision making. This section now clarifies that the decision-making process in question is that of making the DNACPR recommendation and that confusion, conflict or concern about this process leads to incomplete or ambiguous documentation. Concern about how the decision was made and whether it therefore is a valid recommendation would lead to noncompliance. The theme heading needs to make clear which decision-making process is being referred to in the theme.

Discussion

The authors refer to the complex ethical dilemma health care professionals face when considering how to respond in an emergency situation but do not state what the ethical dilemma is.

See my overarching comments above which relate to the discussion.

Conclusion

The conclusion is very short and does not specifically refer to people with a learning disability which seems odd given the paper is focused on people with a learning disability. It also only mentions training on procedures for locating end of life planning documents as the key message, whereas the issue of concerns about their validity due to poor documentation, and potentially poor decision-making conversations, seems equally important from the data and discussion.

Recommendations

The recommendations are very generic and do not specifically address potential challenges for CPR decision making for people with a learning disability. For example it would be good to know the authors' suggestions for how communication and sharing of DNACPR recommendations and other emergency care treatment plans can be shared more effectively for people with a learning disability. Are there likely to be specific challenges that need to be addressed other than a general improvement in how information about emergency care treatment planning is communicated across health and social care organisations. The authors could also suggest how the process of decision making around CPR and other emergency treatment recommendations can be improved for people with a learning disability, other than using accessible methods and support from familiar people. For example, raising awareness of emergency care treatment planning with people with a learning disability and their carers to empower them to be actively involved in the decision-making process. Resources are already available to support people with a learning disability and their families/carers to have these conversations, for example https://www.victoriaandstuart.com/end-of-life-care-planning-toolkit and https://www.resus.org.uk/respect/respect-resources

The Standards for Reporting Qualitative Research (SRQR) document is not completed (supplementary material).

**Reviewer**	**3**
**Name**	**Michalowski, Sabine**
**Affiliation**	
**Date**	**09-Oct-2025**
**COI**	

It's the second time that I am reviewing this paper and it is difficult to carry out this second review, as the authors did not agree with my comments and therefore only marginally, responding to another reviewer's comments, addressed them.

In my previous review, I did not mean to suggest a detailed comparative paper that compares DNACPR decisions and their implementation of persons with or without learning disabilities. My

point was rather that it is difficult to really make sense of the discussions and the interview data and any insights that can follow from this without also being given some indication as to what, precisely, is specific to the group of persons with learning disabilities and what is down to problems with DNACPR decisions in general. I appreciate the limitations of one paper and I think that the focus on persons with learning disabilities is important and welcome and makes a crucial contribution to knowledge, but it is seriously limited by the little explanation of how the authors see the general issues with DNACPRs and what is special about the situation of persons with learning disabilities.

For example, it seems to me that the discussion on p.16, up to line 422, is general and could apply to all DNACPRs, whether or not the person concerned has a learning disability. Some specific discussion of issues specific to persons with learning disabilities continue, but then the discussion becomes general again and I am left wondering what, precisely, how, precisely, the communication, documentation, instinct to preserve life etc. issues identified as problems around DNACPRs are different, and therefore require different solutions, in cases of persons with learning disabilities.

The one sentence conclusion that: "This work highlights the need for greater awareness and training among health and social care staff around procedures for locating end-of-life planning documentation, which alerts to a person's DNACPR status" don't do the paper justice but also do not make any reference at all to the particular issues of persons with learning disabilities.

VERSION 2 - AUTHOR RESPONSE

**Reviewer 1**	**General Overview**	The authors have tightened the introduction and clarified the focus on learning disabilities in DNACPR decisions. They distinguished learning-disability-specific challenges from broader systemic issues. Justified use of LeDeR data and updated the thematic analysis reference (Braun & Clarke, 2021) and provide more detail on thematic analysis steps, consensus-building, and limitations.	Reviewer notes clearer focus and stronger methodological detail.
Response: Thank you for your feedback and the time you have spent reviewing our manuscript.			

<table>
<tr><td></td><td>Remaining Weaknesses – General</td><td>Comparison with general population – clarity throughout paper whether issues identified are unique to learning disability or reflect broader DNACPR challenges and discussion still feels underdeveloped in clarifying how learning disability adds distinct challenges.
Currently, conclusions could appear generalisable to all DNACPR cases, risking dilution of the paper's unique contribution.</td><td>Clarify what is unique to learning disability versus general DNACPR issues.</td></tr>
<tr><td colspan="4">Response: Thank you for this important observation. We agree that many of the challenges identified in this study have also been reported in DNACPR implementation in the general population.

We have revised the manuscript to clarify this distinction more explicitly.

In the Introduction, we now situate the study within the broader DNACPR literature, citing evidence from the general population to highlight that such implementation challenges occur across healthcare settings. In the Discussion, we have added a new section that reflects on why these challenges may be particularly pronounced for people with a learning disability. In particular, we highlight how uncertainty arising from breakdowns in information sharing and documentation issues interact with known inequities in end-of-life care, including reduced involvement in decision-making, reliance on proxy decision-makers, and exposure to assumptions about quality of life. These revisions clarify both the general relevance of the findings and the specific contribution of the study to understanding DNACPR implementation in the context of learning disability.</td></tr>
<tr><td></td><td>Conceptual Clarity</td><td>Review process queried whether "decision-making process" referred to making a DNACPR recommendation vs. following it, you claim it means both, which risks conceptual vagueness. A sharper distinction or renaming of themes would strengthen analytic clarity.</td><td>Define or separate the two decision-making stages to avoid vagueness.</td></tr>
<tr><td colspan="4">Response: We have renamed this theme to 'Making and enacting DNACPR decisions' and clarified within the analysis the distinction between decision-making relating to formulating DNACPR recommendations and decision-making at the point of implementation. These changes aim to improve conceptual clarity.</td></tr>
</table>

<table>
<tr><td></td><td>Theme Evidence</td><td>Some themes (e.g., “instinct to protect life”) remain weakly evidenced; without stronger data excerpts, these themes risk being perceived as speculative.</td><td>Strengthen data support for weaker themes or reconsider them.</td></tr>
<tr><td colspan="4">Response: Thank you for your feedback. We agree with your comment. A fourth author was involved to help us revise the analysis and address the concerns raised by reviewers about the analysis. We have looked back through our dataset and updated the analysis by removing this theme as we appreciate it relates more to the outcome.</td></tr>
<tr><td></td><td>Policy and Practice Implications</td><td>Policy and practice implications, while rewritten, the discussion could further highlight specific actionable recommendations: Staff training (emergency responders, care home staff, ambulance services). Better documentation systems and cross-organisational communication. Embedding the Mental Capacity Act more firmly in clinical practice.</td><td>Expand policy implications with specific, actionable recommendations.</td></tr>
<tr><td colspan="4">Response: Thank you for this suggestion. The recommendations section has now been clarified with specific actionable recommendations, naming the professionals involved and discussing in more detail.</td></tr>
<tr><td></td><td>Recommendations for Improvement</td><td>To reach a publishable standard, the paper would benefit from:
-Sharper framing of contribution
-Explicitly state what is unique about DNACPR for people with learning disabilities (e.g., communication barriers, assumptions about capacity, structural discrimination).
-Contrast with general DNACPR issues so the reader can see the added value.
-Clarify themes
-Separate the “decision-making process” into distinct categories (recommendation-making vs. action at point of arrest).
-Strengthen theme definitions and ensure quotes align clearly.
-Strengthen evidence base
-Use more direct quotes where possible to support themes (even if brief).
-Acknowledge explicitly where themes are interpretive impressions due to data limitations.</td><td>Improve framing and thematic clarity; add direct quotes and LD-specific recommendations.</td></tr>
</table>

		-Expand discussion of implications<br>-Provide clear, actionable recommendations:<br>Mandatory DNACPR training for staff working with people with learning disabilities. Standardised protocols for ensuring DNACPR visibility across care settings.<br>Policy guidance on integrating learning disability specific considerations into DNACPR practice.	
Response: Thank you for your valued feedback. We hope that the changes we have made (summarised in responses to previous comments) address these issues. We have refined the thematic analysis, including removing a weakly support theme, and strengthened the alignment between themes and the supporting data. We have expanded the discussion to more clearly articulate the specific contribution of the study in relation to people with a learning disability, and strengthened the Recommendations section ton included more specific, actionable suggestions for practice.			
	**Literature Integration**	Incorporate missing key references on DNACPR in the general population. This would help position findings and address reviewer concerns about generalisability.	Add general-population DNACPR references to contextualise findings.
Response: We have included several new references in the introduction citing key literature from the general population for context.			
	**Summary**	Conceptual clarity (uniqueness to learning disability, thematic precision) and stronger policy implications still need work before the paper is likely to be fully publishable.	Further refine conceptual and policy contributions to reach publishable standard.
Response: We hope that our revisions detailed in other comments address this. We have reviewed the entire analysis and involved an additional author to resolve the issues raised through the peer-review process with the analysis.			

**Reviewer 2**	**General Overview**	Thank you for the opportunity to review this resubmission. I have read the revised manuscript and responses to reviewers. There are some discrepancies between the responses provided and the content of the final manuscript, so I am not sure if the response document provided was the final version.	Ensure the final manuscript text fully reflects the rebuttal document.
Response: Apologies for this.			
	**Improvements**	The paper has been substantially re-written with the introduction and methods being much clearer than in the first submission. The description of the themes and the additional illustrative quotes also provide a clearer narrative.	Reviewer acknowledges major structural and narrative improvements.
Response: Thank you for your comment, we really appreciate the feedback we've been given to improve this manuscript.			
	**Key Concern**	However, I still have some major concerns which I do not feel the authors have fully addressed. My key concern with the first submission, also expressed by reviewer 3 and indirectly by reviewer 1, was that it was unclear how the findings of this study illuminate particular challenges with implementation of DNACPR recommendations in people with a learning disability compared to their implementation in the general population. Clearly the authors cannot directly compare reviews of decisions made in the general population as this was not their data set. However, some reflection on whether it is likely that the issues they have identified as creating difficulties in CPR decision making within their study are common to everyone, or in what way they may be more pertinent for people with a learning disability is required. I do not think that the authors have addressed this adequately in the resubmission.	Clarify how findings highlight DNACPR challenges specific to people with a learning disability rather than general issues.

<table>
<tr><td colspan="4">Response: Thank you for this important reflection. We acknowledge our data do not allow comparison with DNACPR recommendations in the general population and have been careful not to imply this. We have strengthened the manuscript to make our contribution more explicit, situating our findings within the general DNACPR literature and reflecting on how the challenges identified may be more pronounced in people with a learning disability, even if they are not unique to this population. We have included additional references in the introduction and added reflections in the discussion about learning disability specific challenges.

In particularly the revised Discussion now demonstrates how challenges such as poor information sharing and unclear documentation may have be particularly impactful for people with a learning disability by interacting with known inequities in end-of-life-care. These revisions clarify how the findings extend beyond general DNACPR issues to provide insight into the issues faced by this population.</td></tr>
<tr><td></td><td>Discussion – Context</td><td>All of the issues identified in the themes could apply to implementation of DNACPR recommendations for all patients. What I feel is lacking from the analysis and discussion is a reflection on why these issues might be particularly problematic for people with a learning disability, drawing on what is known about challenges and inequities in end-of-life decision for people with a learning disability, which the authors refer to in their introduction. What is the link between what is known about inequity in end-of-life decision making for people with a learning disability and what the authors have found in their study?</td><td>Link study findings explicitly to existing evidence on end-of-life inequities for people with a learning disability.</td></tr>
<tr><td colspan="4">Response: Thank you for this helpful observation. We agree that several of the challenges identified in the themes may occur in DNACPR decision-making more broadly. In response to this comment, we have strengthened the Discussion to more clearly reflect on how these issues may be particularly challenging in the context of care for people with a learning disability, drawing on the literature on inequities in end-of-life decision-making for this group referenced in the Introduction.

The revised Discussion now highlights how factors such as communication challenges, the need for accessible information, the involvement of family members and carers in decision-making, and the organisation of care across multiple services may contribute to additional complexity when DNACPR recommendations are considered and implemented for people with a learning disability. These revisions explicitly link the study</td></tr>
</table>

<table>
<tr><td colspan="4">findings to existing literature on issues in end-of-life care, demonstrating how the challenges we identified may be amplified in the context of learning disability and contribute to inequitable outcomes in practice.</td></tr>
<tr><td></td><td>Wider Context</td><td>While acknowledging that this study focuses on lack of compliance with DNACPR recommendations for people with a learning disability I reiterate my previous comment in my original review that the discussion would be strengthened by placing the findings in the wider context of compliance with DNACPR recommendations in the general population. I note the authors' response that they have no evidence for this. I previously suggested referencing the 2012 NCEPOD report and the study by Perkins et al on DNACPR decision making. Both of these would be useful in this context. I am surprised the authors could not identify the Perkins study (https://pubmed.ncbi.nlm.nih.gov/27077163/). Also consider more recent publications on the implementation of ReSPECT in primary care (https://pubmed.ncbi.nlm.nih.gov/39304311/, https://pubmed.ncbi.nlm.nih.gov/39487818/). While these are not directly comparable, they would enrich the discussion.</td><td>Add general-population DNACPR literature (e.g., NCEPOD 2012, Perkins et al., ReSPECT) to provide wider context.</td></tr>
<tr><td colspan="4">Response: Thank you for these helpful suggestions. In response to this comment, we have revised the Introduction to better situate the study within the wider literature on DNACPR decision-making and implementation in the general population. We have now incorporated references to the NCEPOD report and the study by Perkins et al., which highlight broader challenges in DNACPR decision-making and documentation. We have also included recent literature on the implementation of ReSPECT processes in primary care to provide additional context for how DNACPR and related end-of-life planning recommendations are implemented in practice. While these studies focus on the general population rather than specifically on people with a learning disability, they help contextualise our findings and illustrate that some of the challenges identified in this study may reflect wider issues in DNACPR implementation. These additions aim to strengthen the discussion by situating the study within the broader DNACPR literature while maintaining the focus on the context of care for people with a learning disability.</td></tr>
</table>

	**Abstract**	Abstract. The conclusion still does not refer to people with a learning disability which is the focus of this study.	Mention people with a learning disability explicitly in the abstract conclusion.
Response: Thank you for highlighting this. We have revised the conclusion of the Abstract to explicitly refer to people with a learning disability, ensuring that the focus of the study is clearly reflected.			
	**Introduction**	Final sentence second paragraph does not make sense. Clinicians must justify their DNACPR decision if the patient wishes for them? Fifth paragraph: the reference for the UK survey of residential homes is missing in the text (reference 12). This study did not focus on people with a learning disability but was a general survey of DNACPR experiences in care homes during COVID and should be presented as such. Similarly, the Ombudsman report did not refer solely to people with a learning disability.	Correct unclear sentence and ensure references are complete and accurately contextualised.
Response: Thank you for highlighting these issues. We have revised the final sentence of the second paragraph in the Introduction to clarify its meaning. We have also added the missing in-text citation for the UK survey of residential homes. In addition, we have amended the text describing both the residential care home survey and the Ombudsman report to clarify that these sources refer to DNACPR decision-making more broadly and do not specifically focus on people with a learning disability. These references are included to provide context for wider challenges in DNACPR decision-making and implementation.			
	**Findings – Overarching Theme**	Overarching theme: instinct to preserve life. I find it difficult to assess a theme that is not supported by any data/quotes.	Provide evidence or remove the unsupported “instinct to preserve life” theme.

Response: Thank you for highlighting this issue. We acknowledge that the previous version of the manuscript did not include quotes to support it, as doing so often required including large sections of the reviews. In response to this feedback, we revisited the dataset and involved a fourth author in reviewing the analysis to address the concerns raised by reviewers. As part of this process, the thematic structure was revised and the theme has now been removed. We have revised the structure of the themes, focusing on three main themes and two cross cutting factors.			
	**Findings – Emergencies**	Theme on emergencies: There is no evidence from the data presented that inconsistency in access to health care for the person with a learning disability impacts on challenges related to an emergency scenario. Not having access to the records in this situation is likely to apply to anyone who has a cardiac arrest.	Clarify or justify how the emergency-care findings are specific to people with a learning disability.
Response:<br><br>Thank you for this helpful observation. We agree that difficulties accessing DNACPR documentation during emergency situations are not unique to people with a learning disability and may occur in anyone who has a cardiac arrest. We have clarified this distinction in the revised manuscript.			
We have added a reflection in the discussion about this on page 15, line 404, to make this point. We now acknowledge that whilst these challenges might affect any patient, people with a learning disability are more likely to live in settings where medical documentation may not be as readily accessible as in clinical environments. This addition clarifies that while such challenges may affect any patient they may contribute to greater uncertainty in decision making for people with a learning disability.			
	**Findings – Decision Making**	Theme on decision making. This section now clarifies that the decision-making process in question is that of making the DNACPR recommendation and that confusion, conflict or concern about this process leads to incomplete or ambiguous documentation. Concern about how the decision was made and whether it therefore is a valid recommendation would lead to non-compliance. The theme heading needs to make clear which decision-making process is being referred to in the theme.	Revise the theme heading to specify which decision-making stage it covers.

Response: thank you for this helpful feedback. We accept that the theme heading was vague and have revised it from "Decision-making processes" to "Making and enacting DNACPR decisions." This change clarifies that the theme concerns the process of making and enacting DNACPR recommendations specifically.<br><br>We have also strengthened the closing paragraph of this theme to make the link between concerns about the decision-making process and non-compliance more explicit (page 14, line 384).			
	**Discussion**	The authors refer to the complex ethical dilemma health-care professionals face when considering how to respond in an emergency situation but do not state what the ethical dilemma is. See my overarching comments above which relate to the discussion.	Define clearly what the stated ethical dilemma entails.
Response: Thank you for highlighting this point. We have clarified this section of the Discussion to explain that the ethical dilemma arises when healthcare professionals encounter uncertainty about how to proceed and whether or not to adhere to a DNACPR recommendation during an emergency. This is about balancing the need to adhere to documented end-of-life decisions and the professional duty to attempt life-saving treatment.			
	**Conclusion**	The conclusion is very short and does not specifically refer to people with a learning disability which seems odd given the paper is focused on people with a learning disability. It also only mentions training on procedures for locating end-of-life planning documents as the key message, whereas the issue of concerns about their validity due to poor documentation and potentially poor decision-making conversations seems equally important from the data and discussion.	Extend the conclusion to emphasise learning-disability focus and documentation/decision-quality issues.
Response:<br><br>Thank you for this helpful observation. We have revised the conclusion to refer to people with a learning disability and to better reflect the key findings of the study. The revised conclusion now highlights the importance of both locating DNACPR documentation in emergency situations,and the importance of clear documentation and transparent decision-making processes, as concerns about the validity of DNACPR recommendations were an important theme in the data.			

	**Recommendations**	The recommendations are very generic and do not specifically address potential challenges for CPR decision making for people with a learning disability… (Full multi-paragraph comment including resource links).	Develop detailed, LD-specific recommendations for communication, empowerment, and shared-decision processes.
Response: Thank you for this helpful suggestion. We have revised the recommendations section to ensure they more clearly reflect the context of DNACPR decision-making for people with a learning disability. The revised text now explicitly refers to the application of Mental Capacity Act principles, the use of accessible communication, and the involvement of family members and carers in best-interest decision-making. We have also clarified the need for targeted training for staff working in services supporting people with a learning disability, including care home staff, support workers and emergency responders, and emphasised the importance of communication across health and social care services involved in the care of people with a learning disability. These revisions aim to ensure that the recommendations more directly address the challenges identified in the study.			
	**SRQR Checklist**	The Standards for Reporting Qualitative Research (SRQR) document is not completed (supplementary material).	Submit a completed SRQR checklist with the revised paper.
Response: Thank you for noting this, and apologies for this oversight, this has now been completed and added to the supplementary material.			
**Reviewer 3**	**General Comment**	It's the second time that I am reviewing this paper and it is difficult to carry out this second review, as the authors did not agree with my comments and therefore only marginally, responding to another reviewer's comments, addressed them.	Reviewer feels earlier feedback was not sufficiently addressed and only partially incorporated.

Response: Thank you for taking the time to review our manuscript again. We greatly appreciate the reviewer's engagement with the paper and apologise if our previous responses gave the impression that your comments had not been fully considered. In revising the manuscript for this round, we have carefully revisited the issues raised in your earlier review, particularly regarding the need to clarify how the challenges identified relate specifically to the context of care for people with a learning disability. We hope these changes better address the concerns raised.

	**Clarification of Focus**	In my previous review, I did not mean to suggest a detailed comparative paper that compares DNACPR decisions and their implementation of persons with or without learning disabilities. My point was rather that it is difficult to really make sense of the discussions and the interview data and any insights that can follow from this without also being given some indication as to what, precisely, is specific to the group of persons with learning disabilities and what is down to problems with DNACPR decisions in general. I appreciate the limitations of one paper and I think that the focus on persons with learning disabilities is important and welcome and makes a crucial contribution to knowledge, but it is seriously limited by the little explanation of how the authors see the general issues with DNACPRs and what is special about the situation of persons with learning disabilities.	Clarify explicitly which findings are unique to people with learning disabilities and which reflect general DNACPR issues.

Response: Thank you for this helpful clarification. We appreciate the reviewer's point that the contribution of the paper depends on clearly distinguishing between challenges in DNACPR implementation that occur in the general population and those that may be particularly relevant in the context of care for people with a learning disability. In response to this comment and others calling for a distinction betwen general issues with DNACPR and learning disability specific issues, we have revised the manuscript to clarify this distinction more explicitly.

In the Introduction, we now situate the study within the wider literature on DNACPR in the general population. In the Discussion, we have added a section that reflects on how the challenges identified in this study may occur more prominently in learning disability care contexts due to factors including communication barriers, involvement of family members and support staff, and the complexities of decision-making under the Mental Capacity Act.

We have also revised the Recommendations section to ensure that the implications more clearly reflect the context of care for people with learning disabilities. We hope these revisions clarify the specific contribution of the study.

	**Discussion**	For example, it seems to me that the discussion on p.16, up to line 422, is general and could apply to all DNACPRs, whether or not the person concerned has a learning disability. Some specific discussion of issues specific to persons with learning disabilities continue, but then the discussion becomes general again and I am left wondering what, precisely, how, precisely, the communication, documentation, instinct to preserve life etc. issues identified as problems around DNACPRs are different, and therefore require different solutions, in cases of persons with learning disabilities.	Strengthen the discussion by distinguishing LD-specific challenges and explaining how they differ from general DNACPR issues.

Response: Thank you for this helpful observation. We agree that several of the challenges identified in this study are not unique to DNACPR recommendations involving people with a learning disability and may occur more broadly. In response to this comment, we have revised the Discussion to more clearly acknowledge this and to clarify how these challenges may manifest differently in the context of care for people with a learning disability.

In particular, we now emphasise how communication challenges may be compounded by the need for accessible communication and the involvement of family members, carers and support staff in decision-making processes. We also highlight how people with a learning disability often receive support across multiple services and settings, which may increase the likelihood of DNACPR documentation not being consistently available at the point of emergency decision-making. These revisions aim to clarify that while some of the challenges identified may occur more generally, they may be amplified or take different forms in the context of care for people with a learning disability.

VERSION 3 - REVIEW

Reviewer 1

Name Doody, Owen

Affiliation University of Limerick Faculty of Education and Health Sciences, Nursing and Midwifery

Date 26-Apr-2026

COI

Thank you for revising your paper and addressing the comments made. I think this is a more balanced paper as a result.

**Reviewer**	**3**
**Name**	**Michalowski, Sabine**
**Affiliation**	
**Date**	**27-May-2026**
**COI**	

This is my third review of this paper and I am not sure I can read it with a fresh perspective at this stage. The authors revised the manuscript in light of the comments of various reviewers and, in particular, attempted to address the fundamental concern that it is not clear how the findings regarding the implementation of DNACPRs are specific to persons with learning disabilities. However, the revisions sometimes remain rather on the surface. Eg lines 367/368 simply state that the findings support this, but there is no in-depth explanation as to how that is the case. So my previous reservations still stand, but I would not suggest any more revisions but rather think that the editors need to decide whether, with these limitations, the paper is nevertheless worthy of being published. I am on the fence with regard to this question, but think that despite its flaws, the paper makes a contribution to knowledge and important discussions, highlights the importance of further research and has strengthened the recommendations and therefore think that it is now publishable.

A large chunk of text is crossed out on pp.4-5 which I assume is a mistake, given that the remaining text stops mid-sentence.

There is a typo in line 279 (of instead of or).
