# Supplementary material for: ‘An adrenaline-fueled emergency’: a qualitative thematic analysis of reviews of deaths in England where DNACPR (Do Not Attempt Cardiopulmonary Resuscitation) recommendations are not implemented for people with a learning disability
Source: BMJ Open. 2026 Jun 25;16(6):e107222. doi: 10.1136/bmjopen-2025-107222 (PMC13358332; doi:10.1136/bmjopen-2025-107222)
Supplement: Author's manuscript [file bmjopen-2025-107222.draft_revisions.pdf]

BMJ Open is committed to open peer review. As part of this commitment we make the peer review history of every article we publish publicly available.

When an article is published we post the peer reviewers' comments and the authors' responses online. We also post the versions of the paper that were used during peer review. These are the versions that the peer review comments apply to.

The versions of the paper that follow are the versions that were submitted during the peer review process. They are not the versions of record or the final published versions. They should not be cited or distributed as the published version of this manuscript.

BMJ Open is an open access journal and the full, final, typeset and author-corrected version of record of the manuscript is available on our site with no access controls, subscription charges or pay-per-view fees (http://bmjopen.bmj.com).

If you have any questions on BMJ Open's open peer review process please email info.bmjopen@bmj.com

BMJ Open

"An adrenaline-fueled emergency": A qualitative exploration of instances where DNACPR (Do Not Attempt Cardiopulmonary Resuscitation) recommendations are not followed for people with a learning disability

Journal:	*BMJ Open*
Manuscript ID	bmjopen-2024-089412
Article Type:	Original research
Date Submitted by the Author:	31-May-2024

Complete List of Authors:	Roberts, Christina; University of Central Lancashire, Research Facilitation and Delivery Unit<br>White, Adam; King's College London Institute of Psychiatry Psychology & Neuroscience, Forensic and Neurodevelopmental Sciences<br>Ruck-Keene, Alex; King's College London Institute of Psychiatry Psychology & Neuroscience; King's College London The Dickson Poon School of Law<br>Sheehan, Rory; King's College London Institute of Psychiatry Psychology & Neuroscience, Forensic and Neurodevelopmental Sciences<br>Strydom, André; Institute of Psychiatry Psychology and Neuroscience<br>Chauhan, Umesh; University of Central Lancashire, School of Medicine

Keywords:	Disabled Persons, Health policy < HEALTH SERVICES ADMINISTRATION & MANAGEMENT, Cardiopulmonary Resuscitation

SCHOLARONE™
Manuscripts

BMJ

I, the Submitting Author has the right to grant and does grant on behalf of all authors of the Work (as defined in the below author licence), an exclusive licence and/or a non-exclusive licence for contributions from authors who are: i) UK Crown employees; ii) where BMJ has agreed a CC-BY licence shall apply, and/or iii) in accordance with the terms applicable for US Federal Government officers or employees acting as part of their official duties; on a worldwide, perpetual, irrevocable, royalty-free basis to BMJ Publishing Group Ltd ("BMJ") its licensees and where the relevant Journal is co-owned by BMJ to the co-owners of the Journal, to publish the Work in this journal and any other BMJ products and to exploit all rights, as set out in our licence.

The Submitting Author accepts and understands that any supply made under these terms is made by BMJ to the Submitting Author unless you are acting as an employee on behalf of your employer or a postgraduate student of an affiliated institution which is paying any applicable article publishing charge ("APC") for Open Access articles. Where the Submitting Author wishes to make the Work available on an Open Access basis (and intends to pay the relevant APC), the terms of reuse of such Open Access shall be governed by a Creative Commons licence – details of these licences and which Creative Commons licence will apply to this Work are set out in our licence referred to above.

Other than as permitted in any relevant BMJ Author's Self Archiving Policies, I confirm this Work has not been accepted for publication elsewhere, is not being considered for publication elsewhere and does not duplicate material already published. I confirm all authors consent to publication of this Work and authorise the granting of this licence.

For peer review only

"An adrenaline-fueled emergency": A qualitative exploration of instances where DNACPR (Do Not Attempt Cardiopulmonary Resuscitation) recommendations are not followed for people with a learning disability

Funding

LeDeR (Learning from lives and deaths – people with a learning disability and autistic people), which commissioned and approved this report, is funded by NHS England. For more details see: https://leder.nhs.uk/about. The funders had no role in the analysis of data, preparation of this paper or decision to publish.

Ethical approval

LeDeR has approval from the UK Secretary of State for Health and Social Care to collect and analyse data compliant with Section 251 for the use of confidential patient information without consent for a specific purpose.

Competing Interests

The authors report no conflicts of interests.

Patient and Public Involvement

This research did not involve patient or public involvement.

For peer review only

Abstract

Objectives: Evidence suggests there are numerous issues with DNACPR recommendations for people with a learning disability. Recommendations are not always in place appropriately and sometimes DNACPR recommendations are not followed, though little is known about why or what happens in these cases. This paper aimed to explore what happens in cases where DNACPR is not followed correctly for people with a learning disability leading to undesired CPR.

Methods: Twenty-one reviews of deaths of people with a learning disability from an English national mortality program in which DNACPR recommendations were not followed and a person received CPR were qualitatively synthesized using thematic analysis.

Findings: Through thematic analysis, several themes were identified which describe issues surrounding cases in which DNACPR recommendations are not followed. These related to communication, the instinct to protect life, responding to an emergency, knowledge and awareness of DNACPR, documenting the recommendation and the decision-making process.

Conclusions: This highlights the need for better training for care staff when responding to emergencies in which CPR would be considered, and better awareness of health and social care staff around procedures for locating documentation and alerting to a person's DNACPR status.

Strengths and limitations

- Thematic analysis was completed with rigor with two researchers synthesizing findings.
- Qualitative insights highlight key areas of improvement for practice around DNACPR recommendations.
- Secondary data meant that analysis was limited to second-hand accounts of events, potentially introducing bias.
- The data source did not include information about the appropriateness of DNACPR recommendations, which is an important consideration.

Introduction

Do Not Attempt Cardiopulmonary Resuscitation (DNACPR)*, commonly referred to internationally as DNR (do not resuscitate) or DNAR (do not attempt resuscitation) is a recommendation which provides guidance to healthcare professionals on whether CPR (cardiopulmonary resuscitation) should be performed on an individual in the event of a cardiac or respiratory arrest (NHS, 2023). DNACPR recommendations are intended to protect the individual from unnecessary suffering or loss of dignity in the dying process caused by receiving

For peer review only

CPR, given CPR has a low success rate of between 11 to 18% in older adults (van Gijn et al., 2014) and can lead to adverse clinical outcomes with survival, such as brain damage and increased physical disability (GMC, n.d.).

DNACPR recommendations can be made as part of end-of-life or advance planning discussions or by healthcare professionals and should normally involve consultation with the person involved and/or their family members. These recommendations can be recorded in medical records in several ways, a DNACPR form, a treatment escalation plan (TEP) or a recommended summary plan for emergency care and treatment (ReSPECT). Although a decision whether to make a DNACPR recommendation is a clinical decision, case-law states patients with capacity must be involved in discussions unless it would cause them active harm (R (Tracey) v Cambridge University Hospitals NHS Foundation Trust & Anor, 2014). The patient's views will carry considerable weight but are not determinative unless they can make an advance decision to refuse treatment (which would be recorded separately). A patient cannot demand that CPR be carried out if it is not clinically appropriate, but clinicians would need a clear rationale to make a DNACPR recommendation in the face of a patient's clearly expressed desire that CPR be attempted.

1.1 DNACPR recommendation decision-making for people with a learning disability

Making DNACPR recommendations for people with a learning disability can be particularly challenging as communication and understanding barriers can mean people with a learning disability cannot always participate in the process of making recommendations (Wagemans et al., 2017). This can be because the person lacks capacity to participate due to their level of intellectual disability or an illness. In which case, case-law and guidance for clinicians is for consultation to involve any legal proxy (such as power of attorney), or in their absence, discuss the decision with those interested in the person's welfare, using a best interests recommendation making process (Winspear v City Hospitals Sunderland NHS Foundation Trust, 2015). In relation to adults, the focus of this work, the principles of the Mental Capacity Act (Department of Health, 2005) must always be followed (GMC, n.d.).

Challenges have been documented in end-of-life decision making, including making appropriate DNACPR recommendations, for people with a learning disability. These involve low levels of knowledge about people with a learning disability (Stein, 2008) and inappropriate assumptions about quality of life for people with a learning disability (Tuffrey-Wijne et al., 2014). Tuffrey-Wijne et al. (2014) conducted a 21-month mixed-method study which assessed the implementation of current recommendations designed to improve the care of those with a learning disability in NHS hospitals in England. The findings suggested carers felt inappropriate assumptions and bias influenced staff members' decisions about whether to provide active treatment. These assumptions were based on "erroneous" beliefs that those with a learning

For peer review only

disability have a poorer quality of life and may be heightened by staff fear around behaviour that challenges. Several other potential barriers included difficulties assessing pain or symptom severity in patients with limited communication and a lack of understanding of the Mental Capacity Act (Department of Health, 2005).

High death rates during the COVID-19 pandemic (Public Health England, 2020) prompted scrutiny of end-of-life practices for people from vulnerable groups, and reports emerged about the use of a 'blanket approach' to DNACPR in which DNACPR recommendations were being applied to many people in a setting (e.g. nursing home) at once (Tapper, 2021). A UK study by Fitton et al. (2023) recruited 262 capacity professionals working in care homes (such as Independent Mental Capacity Advocates) in a survey about their experiences during the COVID-19 pandemic. Fifty-five percent of survey respondents reported witnessing DNACPR forms being added without consultation with individuals or families, and a further 28% reported that this was because of a blanket recommendation from clinicians to add DNACPR forms to the files of all residents based on frailty or disability (including leaning disability).

NHS England management issued several letters in response to these concerns. Firstly, a letter was distributed to primary care distribution lists, acute and community trust chief executives in April 2020 reminded colleagues of the importance of not basing treatment decisions on learning disability/autism alone (Murdoch, Banks & Kanani, 2020). A second letter in 2021 reminded clinicians and managers within the health service of existing published guidance (Powis et al., 2021). In 2023, a third letter reiterated statements from previous letters to emphasize that the NHS position is that it is "unacceptable that people have a DNACPR decision on their record simply because they have a learning disability, autism or both" (Powis et al., 2023).

Nevertheless, concerns around unacceptable clinical rationale being used on DNACPR forms persist. In March 2024, a Parliamentary and Health Service Ombudsman report was published which investigates the issues with DNACPR highlighted during the pandemic. Ableism and ageism are called out within the report, and recommendations for improvements include staff training, availability of accessible communication for conversations around end-of-life decisions, updated guidance and improved record-keeping (Parliamentary and Health Service Ombudsman, 2024).

1.2 Following DNACPR recommendations

In addition to concerns about the appropriateness of DNACPR recommendations for people with a learning disability, findings from the 2021 and 2022 LeDeR reports suggest sometimes DNACPR recommendations are not correctly followed. In 2021, DNACPR recommendations were in place in 64% of deaths, though the proportion of deaths which were considered by

For peer review only

reviewers to have evidence of problems with DNACPR processes doubled (White et al., 2022). These concerns persisted in the 2022 LeDeR report (White et al., 2023), where qualitative findings highlighted concerns around DNACPR, with examples of instances where people received CPR despite having an active DNACPR recommendation (White et al., 2023).

To our knowledge, there is little information available in the published literature about the factors or circumstances surrounding cases where DNACPR recommendations are not followed correctly leading to CPR taking place for people with a learning disability. However, there is evidence from the general population. Wong, Duane and Ingraham (2020) conducted a case series of patients without a learning disability who had a cardiac arrest between 2012 and 2018 in the US. Several factors were identified as contributing to the 'undesired CPR', including physicians overriding the recommendation due to the reasons for the arrest (procedural complication, physician error), failure to share and adhere to patients' advanced wishes, and a lack of knowledge of patients' resuscitation status.

Research suggests that sometimes DNACPR recommendations may not be followed due to professionals misunderstanding what a DNACPR recommendation means. O'Brien et al. (2016) found that up to 30% of community nurses and primary physicians believed that patients with a DNACPR order could not receive simple treatments (such as antibiotics or suctioning) or be transferred to hospital, which were higher percentages compared to hospital doctors and nurse groups. Similarly, qualitative evidence suggests in hospital settings there is confusion translating policy into practice for DNACPR recommendations (Robinson, Cupples & Corrigan, 2007). Conflicting understanding about what DNACPR is for and who can apply it in practice was found in a study looking at older adult nurses working in elderly care (Nolan, 2022).

Overall, several factors make DNACPR recommendations for people with a learning disability particularly complex. The COVID-19 pandemic exposed significant health inequalities for people with a learning disability and evidence of unsafe and unjust practices around DNACPR recommendations and further scrutiny of practices around DNACPR decision-making is needed. Little is known about the circumstances surrounding cases in which DNACPR recommendations are not followed and a person receives CPR for people with a learning disability in England. This paper aimed to explore the circumstances in which DNACPR is not followed by thematically analysing cases of deaths of adults with a learning disability in England in which DNACPR recommendations were not followed resulting in CPR taking place.

For peer review only

Method

2.1 Data source

This work uses data from the Learning from lives and deaths – people with a learning disability and autistic people (LeDeR) program. LeDeR is a national mortality review program in England which aims to address health inequalities for people with intellectual disability and autistic people.

Deaths of people with intellectual disability are reported to the program via the LeDeR website. If a notification of a death is found to be within scope (the person has a confirmed intellectual disability and died whilst being a registered health service user in England), the death will be reviewed by a trained reviewer.

Reviews of deaths contain a number of multiple-choice response questions which give details about the person who died, including their health, living situation, social care involvement, etc. However, most of the review is written by the reviewer, and detailed information about the life and death of the person is provided. The current study used data from the free text fields available in the reviews with a particular focus on a question in which reviewers are invited to expand upon any issues they have identified with DNACPR.

2.2 Sample selection:

The sample of reviews for which a DNACPR recommendation was not followed correctly was selected from a database containing all LeDeR reviews. The LeDeR reviews for adults with a learning disability who died with a DNACPR recommendation in place that was not correctly *followed.* One question on the review was used as a proxy for this: "Was the DNACPR documentation correctly completed and followed". Reviews from 2020 to 2022 were considered as the review question about DNACPR recommendations being followed was not introduced to the review until 2020.

2.3 Sample with DNACPR recommendations not followed correctly

Sixty-six reviews were indicated by the reviewer to have not had DNACPR recommendations followed correctly. Starting from the most recent date of death of the available reviews (December 2022), reviews were read in their entirety by one of the authors to ascertain the availability of information in the review about DNACPR. Cases in which DNACPR recommendations had not been followed resulting in CPR, with sufficient information in the review were retained for the final sample. The sample selection process is outlined in Figure 1.

For peer review only

Figure 1: A flowchart of the selection process for the sample of reviews in which DNACPR recommendations were not followed, resulting in CPR.

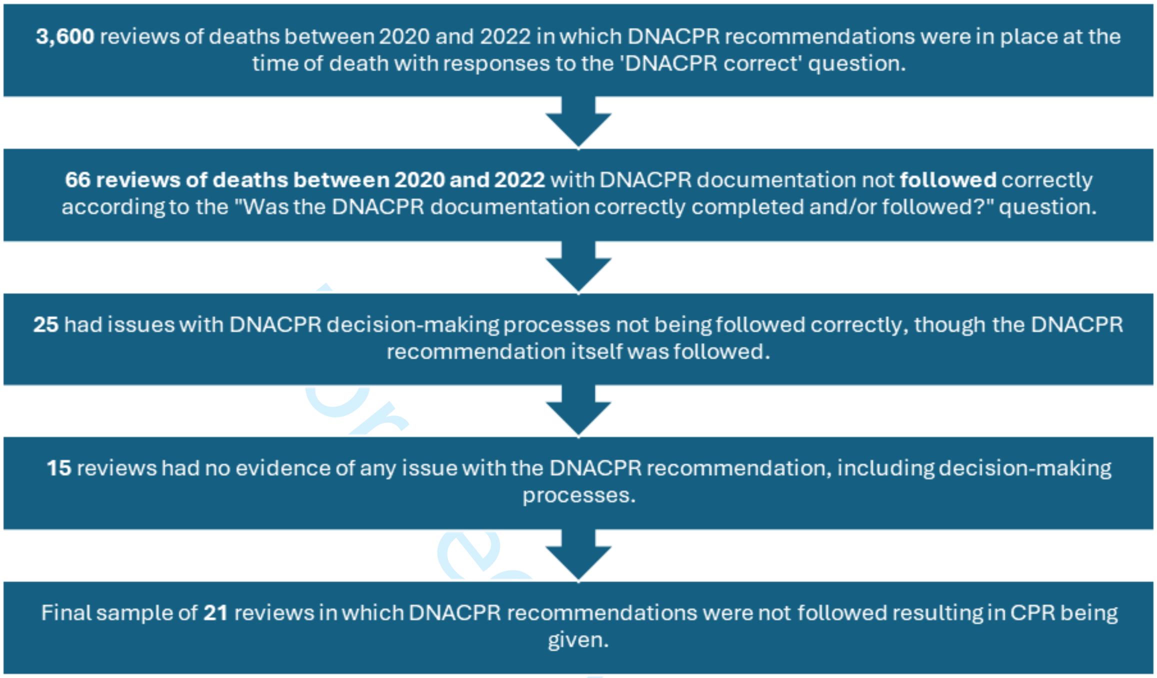

Twenty-one reviews in which reviewers had commented or reflected on issues with DNACPR recommendations not being followed, resulting in CPR, were selected and included in the main sample for qualitative analysis. A second reviewer (AW) independently reviewed this final sample.

2.4 Descriptive statistics

To give context to the qualitative findings, descriptive statistics are reported. Sex, age at death, ethnicity and place of death are reported which are gathered from LeDeR initial reviews.

2.5 Methodology

Inductive thematic analysis (Braun & Clark, 2006) was used to describe themes in the data. In this approach, two researchers independently read the free text fields from the reviews (here reviewers summarise the care and treatment of the person with a learning disability and the events leading to death) and noted down significant themes or issues relating to DNACPR adherence that emerged. MS Excel was used to compile the dataset, and this was used in combination with researcher reflective notes in MS Word to analyse the data.

The researchers frequently met to discuss their findings and refine their notes into thematic categories. Once saturation was reached (no new themes were emerging), the researchers

For peer review only

concluded the thematic analysis. The next step involved consolidation of the themes into detailed descriptions, and the extraction of quotes from reviews to support each theme.

For peer review only

Results

3.1 Demographics

Of the 21 adults with a learning disability whose deaths were reviewed by LeDeR and judged by reviewers to have had DNACPR followed incorrectly resulting in CPR taking place, the average age at death (mean) was 64.7 years. The sample was 38% male, and almost all were denoted as White ethnicity (95%). Fifty-two percent of the sample died in their usual place of residence and 38% died in hospital. Sixty-two percent of the sample died in 2022 and 38% died in 2021.

3.2 Thematic analysis

Following the approach to inductive thematic analysis outlined by Braun and Clark (2006), six steps were taken to thematically analysing the reviews of deaths in which reviewers indicated DNACPR recommendations had not been followed and CPR had taken place.

Familiarization

During this step, two researchers independently read a proportion of the 21 reviews to immerse and familiarise themselves with the data. This involved actively reading and re-reading the reviews. A reflective journal was used to document researchers' thoughts, feelings and ideas about the data at this stage.

Notes at this stage suggested researchers felt several components were already emerging from the data, and information sharing and documentation problems were identified as emerging themes.

"It seems like there are a lot of communication issues and lack of understanding   that could be improved with better training and awareness around DNACPR"

Coding

The next stage involved generating initial codes from the data. This was done mainly by one researcher with frequent discussion and reflections with the second coder. The codes were intended to represent underlying themes within the data and quotes or notes from the reviews were used to support their fit with the data.

Searching for themes

This process involved looking through the initial themes generated during the coding stage and considering how themes relate to one another, overlap and repeat. Iterative coding was done using mind-maps on paper to refine the themes into groups of similar codes. These then became candidates for major themes in the data.

For peer review only

Reviewing themes

During this stage, the initial themes generated were reviewed and their underlying meaning was explored and discussed. Mind-maps were used to group, ungroup and regroup the codes to visualise the best fit of the data.

Defining and naming themes

After reviewing the themes, we discussed how best to name each theme in a way that captures the essence of the theme. Here we reflected on how best to describe each theme and the issues they represent. For example, the initial theme “emergency response” shown in stage 3 became “Responding to an emergency” as we felt this better captured the meaning behind the theme.

The remainder of the results section represents the final stage of thematic analysis: ‘writing up’, where the interpretation of the analysis is described in detail.

Figure 2 shows a map of the data with the codes from the data which contributed to the four main themes.

Figure 2: A map of the data from reviews in which reviewers indicated DNACPR documentation was not followed leading to a person receiving CPR. The four circles represent main themes, and the rectangles surrounding them represent codes synthesized into themes.

For peer review only

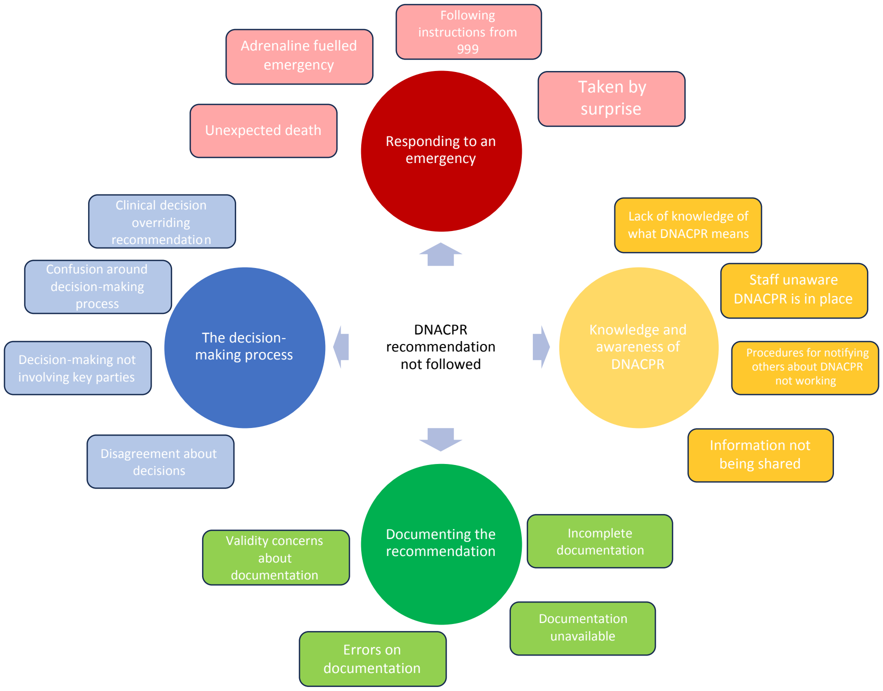

Two overarching themes and four main themes were consolidated from the reviews that represent key issues when following DNACPR recommendations. The overarching themes were Communication and Instinct to protect life. The main themes were: Responding to an emergency, Knowledge and awareness of DNACPR, Documenting the recommendation and The decision-making process. A thematic map of the data is shown in Figure 3.

Figure 3: Final thematic map of the data from reviews in which reviewers indicated DNACPR documentation was not followed leading to a person receiving CPR.

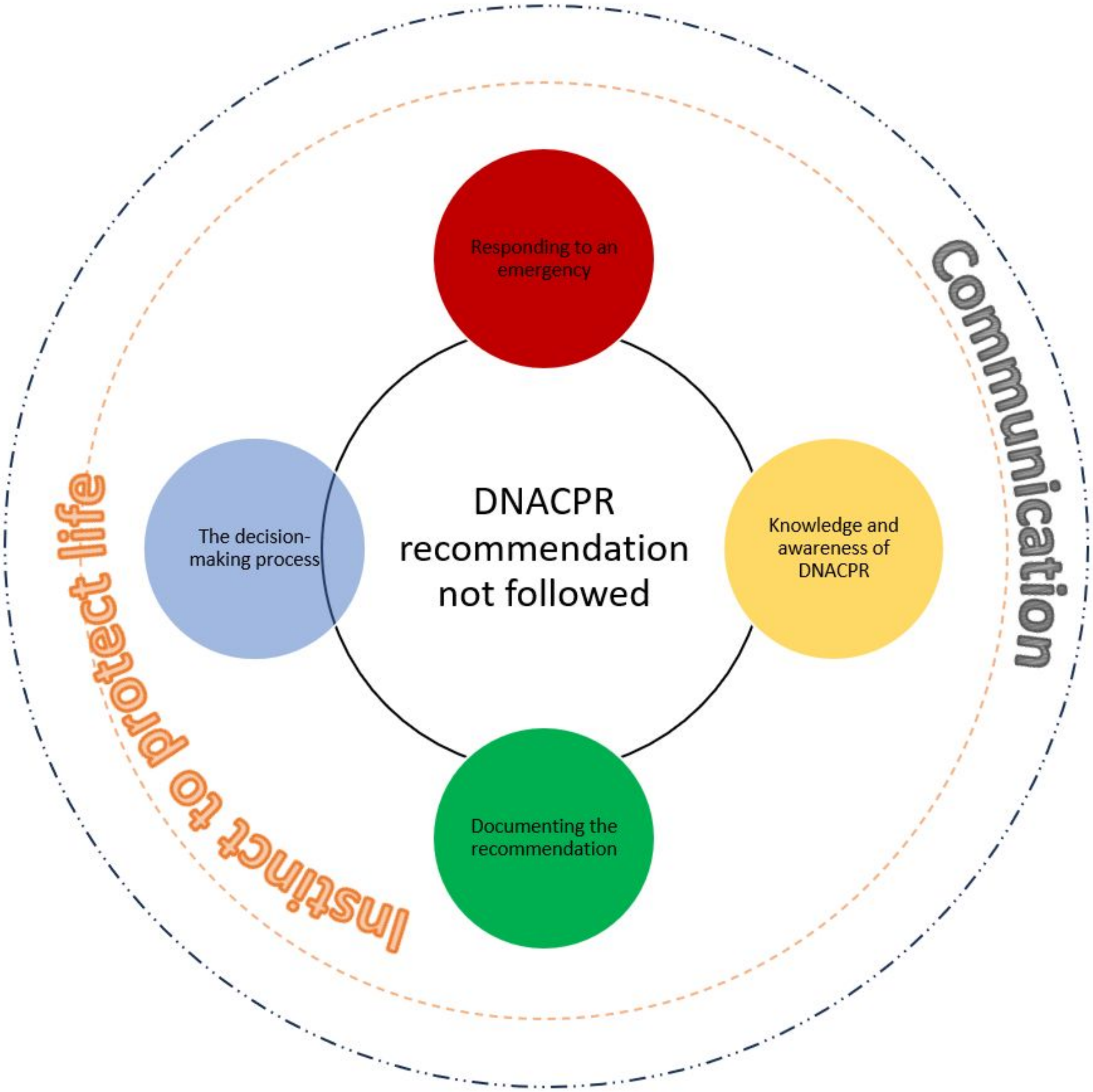

Communication

The first of the two overarching themes is Communication. Communication was felt to be a common thread which weaved through all four main themes.

One key aspect of communication was information sharing, which involved professionals involved in care communicating about DNACPR recommendations to one another and between different health and social care systems. Breakdowns in these channels of communication often led to lack of awareness or knowledge of recommendations, and when faced with emergency situations, CPR would be commenced as default. Communication is also important to support understanding of DNACPR and plans around emergency care.

"It is reported by Sister that she was upset that resuscitation was being commenced as the DNA CPR was not known to the ambulance service" Reviewer 19

Documentation helps to communicate DNACPR recommendations between care pathways, and problems with documentation can present difficulties for clinicians when considering resuscitation options. Communication extends to the decision-making process, and difficulties with communicating the process could lead to confusion, and ultimately doubt over the validity or appropriateness of DNACPR recommendations.

"There was a ReSPECT form in place however there was a lot of confusion around this. It had not been clearly communicated with mother or care team." - Reviewer 20

Open channels of communication can help avoid issues with following DNACPR recommendations and ensuring DNACPR recommendations are appropriate. Ensuring all parties involved in the health and care of the person are aware of the recommendation, and have easy access to the correctly completed documentation, is key to ensuring wishes around DNACPR can be followed.

Instinct to protect life

The second overarching theme was Instinct to protect life. This theme represents the care and dedication of family, carers, health and social care staff to ultimately protect the people they care for. Some reviewers reflected compassionately on how the people involved were doing their best. In situations where DNACPR status was uncertain, erring on the side of life was the appropriate course of action. This course of action was particularly evident in cases where 999 were called, and the natural response is to attempt to save a life, especially where DNACPR documentation was unavailable, disputed, or incomplete.

The instinct to protect, and act appropriately on behalf of a person with a learning disability who is unwell, is evident in decision-making processes, where individuals with different levels of caring responsibility face challenges coming to decisions about end-of-life plans. These challenges when making complex, life-changing decisions are important, though sometimes when agreement cannot be reached, confusion and distress can be caused to those faced with the decision about whether to resuscitate.

Following through with a recommendation not to attempt to resuscitate a person can be emotionally challenging, and sometimes confusion around what to do was "traumatic" for the people responding to the situation. It is felt that underlying some of the events described by reviewers was the instinct by those involved to protect, or do the right thing for, the person with a learning disability. The challenge of overcoming the instinct to intervene when a person

For peer review only

is unresponsive can be difficult to navigate, particularly when there is a close relationship between the person responding to the situation and the person who is unresponsive.

Responding to an emergency

The first theme represents the challenges faced when responding to an emergency. Often emergencies were sudden, unexpected and took paid carers or health professionals by surprise. Several of the reviews commented on the sudden nature of the person's collapse, and how those around them responded instinctively and called for help from emergency services. It was emergency services involvement that sometimes triggered CPR to be commenced, as those attending to the collapse would "follow instructions" from the call handlers.

"Staff were adrenaline fuelled in an emergency situation and followed 999 call handler's instructions" - Reviewer 1

In some cases, CPR attempts would be ceased once paramedics attended and checked patient records or questioned their resuscitation status. However, this information was not always readily available, which would lead to confusion around how to proceed. Particularly relevant to this theme were situations in which individuals collapsed out of hospital, in residential or non-medical settings, where the natural course of action was to call for help from emergency services and follow instructions.

"After paramedic obtained medical advice they found records stated person was not for resuscitation and attempts stopped" - Reviewer 5

One example was given of a person who collapsed suddenly in an accident. The circumstances surrounding the collapse meant the correct course of action was to attempt to save the person's life, given the events were not related to the natural progression of their condition.

"It was recognised by the staff who found [Name] that she had a DNACPR, they phoned the on-call director who advised them to start CPR given the circumstances she had been found in." - Reviewer 15

Knowledge and awareness of DNACPR

The second theme is around the knowledge and awareness of DNACPR of people involved in the care of the person with a learning disability. Issues around knowledge and awareness of DNACPR were on different levels. Firstly, there is the issue of people being unaware of a DNACPR recommendation being in place at all. Being unaware of an active, valid DNACPR meant those attending to the collapsed patient proceeded in the natural and appropriate way by considering resuscitation.

"Staff denied any knowledge of a DNACPR" - Reviewer 17

The reasons for a lack of knowledge and awareness of a DNACPR recommendation often relate to communication issues between the clinicians involved in the decision-making and others involved in their care. However, often communication around DNACPR status was hindered by alerting systems not working as they should. In hospital, an example was given of a patient with a blank bed board and no wristband, meaning staff were not aware of DNACPR status. Other reasons for a lack of knowledge and awareness of a DNACPR recommendation related to documentation, which is described in the next theme.

"CPR was commenced as records viewed by staff did not reflect an alert that she had a DNACPR in place, during the efforts to resuscitate her it was noted that the alert was now evident on her records so CPR stopped" - Reviewer 18

Secondly, sometimes individuals caring for the person with a learning disability struggled to understand what a DNACPR recommendation means in practice, so that when the situation of a collapse presented itself, they called emergency services and followed instructions for CPR. Some reviewers commented on the distress this confusion caused for the people involved in the situation, and some examples highlighted the "panic" and "upset" felt by particularly care home staff who did not feel confident in knowing what to do when someone collapses with a DNACPR recommendation in place.

"CPR was commenced following instruction from 999. There was a ReSPECT form in place however there was a lot of confusion around this. It had not been clearly communicated with mother or care team." - Reviewer 20

Documenting the recommendation

The third theme is Documenting the recommendation, which captures the manifold issues described by reviewers with relevant documentation, including DNACPR forms, ReSPECT forms and TEP forms. Two main issues emerged with documentation. The first was the availability of the documentation, with some reviewers describing situations in which documentation could not be found. Unavailable documentation often led to relevant professionals being unaware of the recommendation, or unable to follow the recommendation without documented evidence, both situations leading to CPR being commenced. This was a frequently described issue by reviewers.

"Staff were unable to locate DNACPR/ReSPECT form although they knew Stuart was not to be resuscitated. Arrest trolley called for." - Reviewer 5

"Carers called emergency services and carried out CPR for approximately 10 minutes until they located the TEP and then ceased." - Reviewer 9

For peer review only - http://bmjopen.bmj.com/site/about/guidelines.xhtml

The second issue was the quality of the documentation. Some reviewers described situations in which sections of the DNACPR form or other documentation were incomplete or incorrect. Sometimes documentation was not signed off correctly, or sections were missing on the form. Rightfully, sometimes professionals questioned the validity of the recommendation when presented with incomplete or incorrect documentation, leading to CPR going ahead.

"It does not appear that the GP signed off [Name]'s emergency health care plan or advance care plans, nor did they attend meetings where these were formulated." - Reviewer 12

In other cases, the validity of the DNACPR documentation was questioned due to the available documented decision being "old". This led to confusion about how to proceed and distress for those involved in the situation.

"Ambulance log states DNACPR in place staff stated, " It is not valid as it is old". Recorded that valid DNACPR had been viewed." - Reviewer 13

The decision-making process

The final theme relates to issues with the decision-making process around DNACPR and concerns or disagreements about how the recommendation was made. Given the gravity of DNACPR recommendations, different opinions about how to proceed in the best interests of the person with a learning disability are to be expected. One reviewer described a situation in which a person's family had agreed with a DNACPR recommendation, but the recommendation was changed following objections from the care team. This example also demonstrates the issue of DNACPR being used as a proxy for no treatment.

"The DNACPR completed at that time in discussion with her family advised no treatment due to her poor quality of life. This decision was changed following objections from her care team."- Reviewer 15

Sometimes issues with the decision-making process centered around considering the wishes and feelings of the person with a learning disability. One example was given of a person who did not want a DNACPR to be put in place, but clinicians disagreed and recommended DNACPR. This led to DNACPR documentation being incomplete at the time of the person's collapse and a distressing situation for carers. Prolonged and difficult decision-making processes around DNACPR led to incomplete or confusing documentation and distress for those involved in the process.

"It was also noted that the Care Home paperwork ("Covid-19 Hospital Summary) recorded that the patient said "Doctors have previously put DNAR in place without asking me. I have made it clear that I want to be resuscitated" - Reviewer 1

For peer review only

Discussion

This paper explored DNACPR recommendations for people with a learning disability aimed to look at what happens in cases where DNACPR recommendations were not followed, and a person received CPR. A qualitative thematic analysis found that overall communication and the instinct to protect life were the underlying themes behind the reviews. Often recommendations were not communicated effectively and when in doubt, those responsible for the care of people with a learning disability rightly respond by attempting CPR, acting upon the instinct to protect life. Four themes described different issues with following DNACPR recommendations. These were responding to an emergency, knowledge and awareness of DNACPR recommendations, documenting the recommendation and the decision-making process.

Communication issues underpinned many of the cases in which DNACPR recommendations were not followed. These issues ranged from communicating the DNACPR recommendation itself, to communication issues between individuals involved in the decision-making process. To improve practice around DNACPR recommendations for people with a learning disability, facilitating better communication is key. This involves communication between professionals, family members and the person with a learning disability around DNACPR status, but also improving communication during the decision-making process for people with a learning disability both with and without capacity. Reviewer's comments rarely mentioned the use of the Mental Capacity Act (Department of Health, 2005), possibly because there is currently no invitation for reflection on this within the review. However, it is important to ensure the principles of the Act are understood and applied in conversations around DNACPR. People lacking capacity should not be excluded from conversations, instead practicable steps should be taken to facilitate communication and involvement (unless this involvement would cause harm).

Previous research has indicated misunderstanding of DNACPR recommendations is common even amongst nursing professionals working in elderly adult care (Nolan, 2022), and although this came through in the theme 'Knowledge and awareness of DNACPR', the data was not suitable for looking in detail at levels of understanding of DNACPR by professionals caring for people with a learning disability. Understanding and knowledge issues are potentially a key barrier to both following DNACPR recommendations and ensuring recommendations are appropriate. More work is needed to explore how health and social care professionals of DNACPR recommendations and how this may impact upon procedures being followed correctly.

A strength of this paper is that it provides rich qualitative findings which give insight into the issues surrounding cases in which DNACPR recommendations are not followed for people with

For peer review only

a learning disability, leading to CPR taking place. A rigorous qualitative approach was undertaken which identified key areas of improvement for practice around DNACPR recommendations.

As we used secondary data (reviewers' comments about DNACPR), analysis was limited by the detail reviewers chose to go into about the events surrounding a death. The data source meant the researchers did not see the DNACPR documentation and did not have first-hand accounts of the events surrounding situations in which CPR was considered. Although reviewers are experienced professionals who receive extensive training, it is likely the analysis does not capture a full evaluation of the circumstances surrounding DNACPR and may be subject to bias. This meant that the data source was not suitable for looking in detail at important aspects of the DNACPR decision-making process itself.

This work highlights the need for greater awareness among health and social care staff around procedures for locating end-of-life planning documentation which alerts to a person's DNACPR status. Future research should monitor the extent to which DNACPR recommendations are in place appropriately for people with a learning disability, to assess whether practices around DNACPR are improving in line with recent recommendations (Parliamentary and Health Ombudsman, 2024). An interesting line for future work would be to investigate how the Mental Capacity Act (Department of Health, 2005) is being used in the DNACPR decision-making process.

For peer review only

References

Braun, V., & Clarke, V. (2006). Using thematic analysis in psychology. *Qualitative research in psychology*, *3*(2), 77-101.

Department of Health. (2005). *Mental Capacity Act.* London: HMSO.

Evans, N. (2023). DNACPR for people with learning disabilities: How nurses can help avoid inappropriate DNACPR recommendations for people with learning disabilities. *Learning Disability Practice*, *26*(4), 15–16. https://doi.org/10.7748/ldp.26.4.15.s6

Fitton, E., Kuylen, M., Wyllie, A., Michalowski, S., Bhatt, V., & Martin, W. (2023). Managing DNACPR Recommendations in Residential Care: Towards Improved Training for Social Care and Capacity Professionals. *The British Journal of Social Work*.

GMC (n.d.). *Cardiopulmonary resuscitation (CPR)*. Professional Standards - GMC. https://www.gmc-uk.org/professional-standards/professional-standards-for-doctors/treatment-and-care-towards-the-end-of-life/cardiopulmonary-resuscitation-cpr https://www.bailii.org/ew/cases/EWHC/QB/2015/3250.html

Murdoch, C., Banks, R., Kanani, N. (2020) Do Not Attempt Cardiopulmonary Resuscitation DNACPR letter 3 April 2020 Coronavirus » Do Not Attempt Cardiopulmonary Resuscitation DNACPR letter 3 April 2020 (england.nhs.uk)

NHS. (2023, December 14). Do not attempt cardiopulmonary resuscitation (DNACPR) recommendations. NHS choices. https://www.nhs.uk/conditions/do-not-attempt-cardiopulmonary-resuscitation-dnacpr-recommendations/

Nolan, J. P. (2022). A mixed-methods study of DNACPR in nursing practice.

O'Brien, H., Scarlett, S., Brady, A. M., Harkin, K., Kenny, R. A., & Moriarty, J. (2017). Do-not-attempt-resuscitation (DNAR) orders: understanding and interpretation of their use in the hospitalised patient in Ireland. A brief report. *Journal of Medical Ethics*, *44*(3), 201–203. https://doi.org/10.1136/medethics-2016-103986

Parliamentary and Health Service Ombudsman (2024) End of life care: improving DNACPR conversations for everyone | Parliamentary and Health Service Ombudsman (PHSO). https://www.ombudsman.org.uk/publications/end-life-care-improving-dnacpr-conversations-everyone

Powis, S., Doyle, A., Wee, B., Cahill, T., Banks, R., May, R. (2023) Do not attempt cardiopulmonary resuscitation (DNACPR) and people with a learning disability and or autism

For peer review only

NHS England » Do not attempt cardiopulmonary resuscitation (DNACPR) and people with a learning disability and or autism

Powis, S., May, R., Kanani, N., Wee, B., Murdoch, C., Banks, R. (2021) Coronavirus *» Do Not Attempt Cardiopulmonary Resuscitation (DNACPR) and people with a learning disability and or autism*. https://www.england.nhs.uk/coronavirus/documents/c1146-do-not-attempt-cardiopulmonary-resuscitation-dnacpr-and-people-with-a-learning-disability-and-or-autism/

Public Health England. (2020). *COVID-19: deaths of people with learning disabilities*. GOV.UK. https://www.gov.uk/government/publications/covid-19-deaths-of-people-with-learning-disabilities

R (Tracey) v Cambridge University Hospitals NHS Foundation Trust & Anor [2014] EWCA Civ 33 https://www.judiciary.uk/wp-content/uploads/JCO/Documents/Judgments/tracey-v-cambridge-nhs-trust.pdf

Robinson, F., Cupples, M., & Corrigan, M. (2007). Implementing a resuscitation policy for patients at the end of life in an acute hospital setting: qualitative study. *Palliative Medicine*, *21*(4), 305–312. https://doi.org/10.1177/0269216307077817

Stein, G. S. (2008). Providing Palliative Care to People with Intellectual Disabilities: Services, Staff Knowledge, and Challenges. *Journal of Palliative Medicine*, *11*(9), 1241–1248. https://doi.org/10.1089/jpm.2008.0130

Tapper, J. (2021, February 15). Fury at 'do not resuscitate' notices given to Covid patients with learning disabilities. *The Guardian*. https://www.theguardian.com/world/2021/feb/13/new-do-not-resuscitate-orders-imposed-on-covid-19-patients-with-learning-difficulties

Tuffrey-Wijne, I., Goulding, L., Gordon, V., Abraham, E., Giatras, N., Edwards, C., ... & Hollins, S. (2014). The challenges in monitoring and preventing patient safety incidents for people with intellectual disabilities in NHS acute hospitals: evidence from a mixed-methods study. *BMC health services research*, *14*, 1-13.

van Gijn, M. S., Frijns, D., van de Glind, E. M., C. van Munster, B., & Hamaker, M. E. (2014). The chance of survival and the functional outcome after in-hospital cardiopulmonary resuscitation in older people: a systematic review. *Age and Ageing*, *43*(4), 456-463.

Wagemans, A. M. A., Schrojenstein Lantman, de V. H. M. J., Proot, I. M., Bressers, A. M., Metsemakers, J., Tuffrey, W. I., Groot, M., & Curfs, L. M. G. (2017). Do-Not-Attempt-Resuscitation orders for people with intellectual disabilities: dilemmas and uncertainties for ID physicians and trainees. The importance of the deliberation process. *Journal of Intellectual Disability Research*, *61*(3), 245–254. https://doi.org/10.1111/jir.12333

For peer review only

White, A., Sheehan, R., Ding, J., Roberts, C., Magill, N., Keagan-Bull, R., Carter, B., Ruane, M., Xiang, X., Tuffrey-Wjine, I., Chauhan, U., Strydom, A. (2022) Learning from Lives and Deaths - People with a learning disability and autistic people (LeDeR) report for 2021. Other. UNSPECIFIED, London, UK.

White, A., Sheehan, R., Ding, J., Roberts, C., Magill, N., Keagan-Bull, R., Tuffrey-Wjine, I., Chauhan, U., Strydom, A. (2023). Learning from lives and deaths-people with a learning disability and autistic people (Leder) report for 2022. *King's College London*.

Winspear vs City Hospitals Sunderland NHS Foundation Trust (2015). EWHC 3250 (QB)

Wong, J., Duane, P. G., & Ingraham, N. E. (2020). A case series of patients who were do not resuscitate but underwent cardiopulmonary resuscitation. *Resuscitation*.

For peer review only

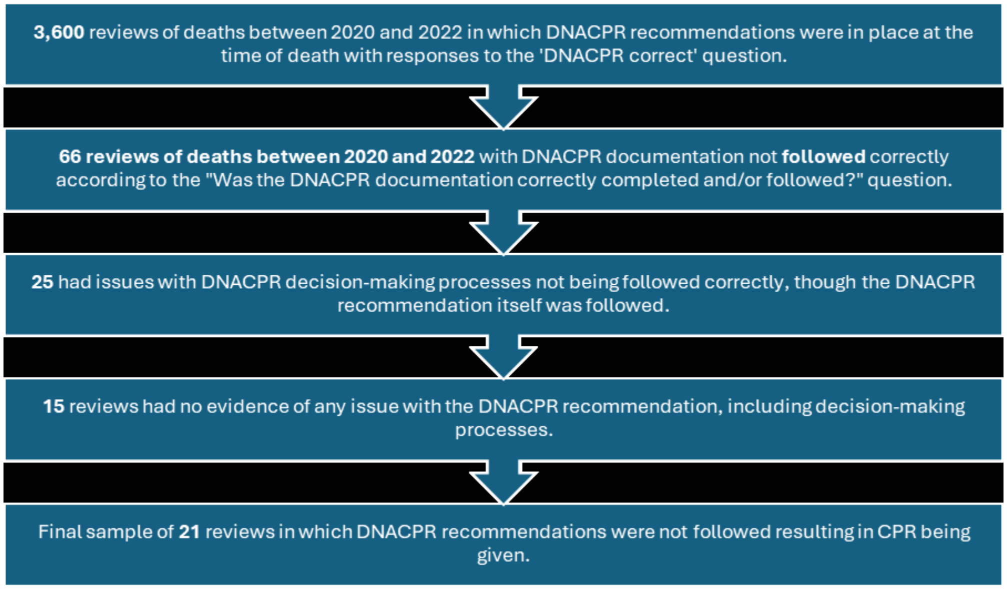

Figure 1: A flowchart of the selection process for the sample of reviews in which DNACPR recommendations were not followed, resulting in CPR.

388x226mm (59 x 59 DPI)

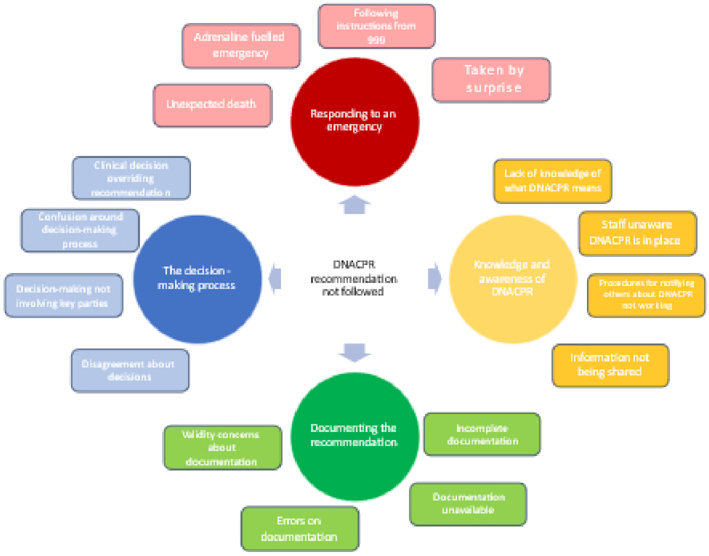

Figure 2: A map of the data from reviews in which reviewers indicated DNACPR documentation was not followed leading to a person receiving CPR. The four circles represent main themes, and the rectangles surrounding them represent codes synthesized into themes.

417x234mm (38 x 38 DPI)

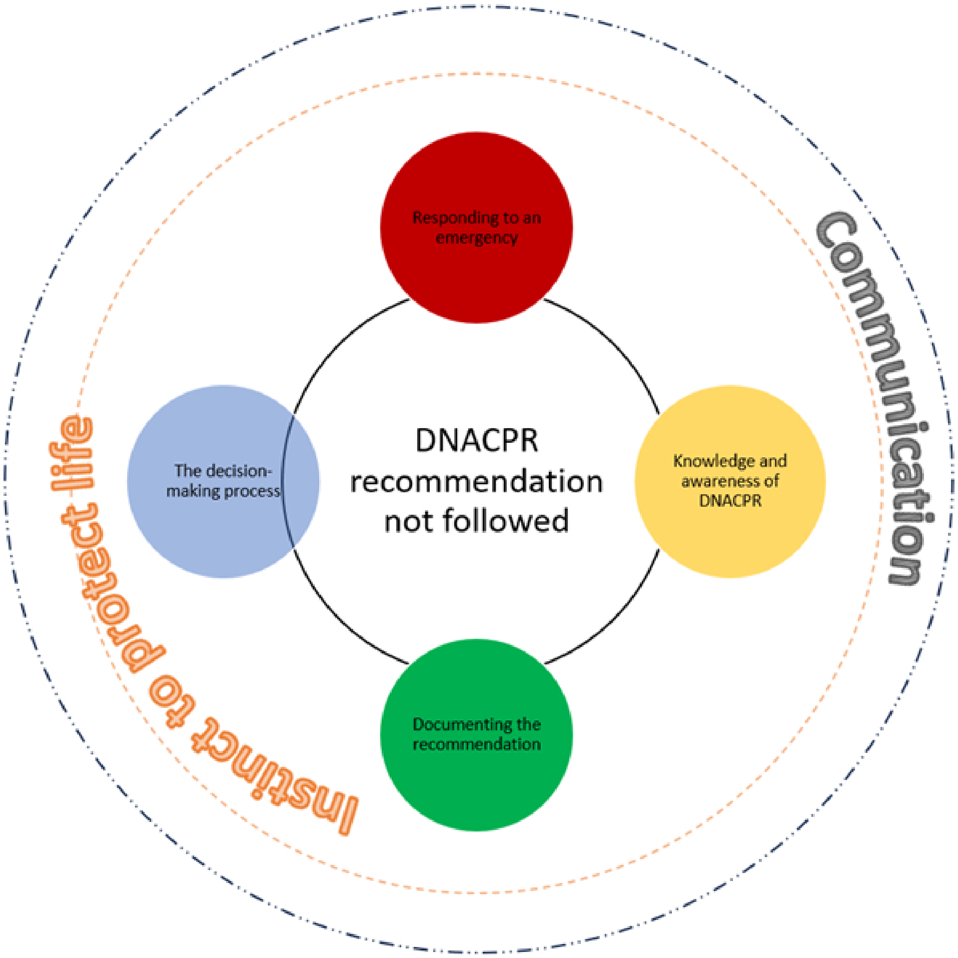

Figure 3: Final thematic map of the data from reviews in which reviewers indicated DNACPR documentation was not followed leading to a person receiving CPR.

417x405mm (38 x 38 DPI)

BMJ Open

"An adrenaline-fueled emergency": A qualitative analysis of reviews of deaths in England where DNACPR (Do Not Attempt Cardiopulmonary Resuscitation) recommendations are not implemented for people with a learning disability

Journal:	*BMJ Open*
Manuscript ID	bmjopen-2025-107222
Article Type:	Original research
Date Submitted by the Author:	29-Jun-2025
Complete List of Authors:	Roberts, Christina; Kingston University, Faculty of Health, Science, Social Care and Education<br>White, Adam; King's College London Institute of Psychiatry Psychology & Neuroscience, Forensic and Neurodevelopmental Sciences<br>Ditzel, Nicola; UCLan, ; King's College London,<br>Ruck-Keene, Alex; King's College London, Institute of Psychiatry, Psychology and Neuroscience<br>Sheehan, Rory; King's College London Institute of Psychiatry Psychology & Neuroscience, Forensic and Neurodevelopmental Sciences<br>Strydom, André; Institute of Psychiatry Psychology and Neuroscience, ; King's College London<br>Chauhan, Umesh; University of Central Lancashire, School of Health
Keywords:	Disabled Persons, Adult palliative care < PALLIATIVE CARE, Cardiopulmonary Resuscitation

SCHOLARONE
MANUSCRIPTS

I, the Submitting Author has the right to grant and does grant on behalf of all authors of the Work (as defined in the below author licence), an exclusive licence and/or a non-exclusive licence for contributions from authors who are: i) UK Crown employees; ii) where BMJ has agreed a CC-BY licence shall apply, and/or iii) in accordance with the terms applicable for US Federal Government officers or employees acting as part of their official duties; on a worldwide, perpetual, irrevocable, royalty-free basis to BMJ Publishing Group Ltd ("BMJ") its licensees and where the relevant Journal is co-owned by BMJ to the co-owners of the Journal, to publish the Work in this journal and any other BMJ products and to exploit all rights, as set out in our licence.

The Submitting Author accepts and understands that any supply made under these terms is made by BMJ to the Submitting Author unless you are acting as an employee on behalf of your employer or a postgraduate student of an affiliated institution which is paying any applicable article publishing charge ("APC") for Open Access articles. Where the Submitting Author wishes to make the Work available on an Open Access basis (and intends to pay the relevant APC), the terms of reuse of such Open Access shall be governed by a Creative Commons licence – details of these licences and which Creative Commons licence will apply to this Work are set out in our licence referred to above.

Other than as permitted in any relevant BMJ Author's Self Archiving Policies, I confirm this Work has not been accepted for publication elsewhere, is not being considered for publication elsewhere and does not duplicate material already published. I confirm all authors consent to publication of this Work and authorise the granting of this licence.

For peer review only

"An adrenaline-fueled emergency": A qualitative analysis of reviews of deaths in England where DNACPR (Do Not Attempt Cardiopulmonary Resuscitation) recommendations are not implemented for people with a learning disability

For peer review only

Authors: **Christina Roberts**, Adam White, Nicola Ditzel, Alex Ruck-Keene, Rory Sheehan, Andre Strydom, Umesh Chauhan

ORCiD: Christina: 0000-0001-6226-0519, Adam: 0009-0002-5456-7523, Nicola: 0009-0005-2090-0537, Alex: 0000-0001-8856-8132, Andre: 0000-0002-2502-6704, Rory: 0000-0002-4164-9661, Umesh: 0000-0002-0747-591X

Author information:

Christina Roberts, MPsych, MSc, Project Manager/Research Fellow

Faculty of Health, Science, Social Care & Education, Kingston University London, UK. Email: christina.roberts@kingston.ac.uk

Adam White, MSc, Research Coordinator

Institute of Psychiatry, Psychology and Neuroscience, King's College London, UK. Email: adam.1.white@kcl.ac.uk

Nicola Ditzel, MA, Research Assistant

School of Medicine and Dentistry, University of Lancashire, Preston, UK. Email: nditzel4@uclan.ac.uk

Alex Ruck- Keene, KC (Hon), Barrister, Visiting Professor

Institute of Psychiatry, Psychology and Neuroscience; The Dickson Poon School of Law, King's College London, UK. Email: alexander.ruck_keene@kcl.ac.uk

Rory Sheehan, MBChB, MSc, MRCPsych, PhD, Senior Clinical Lecturer in Forensic Neurodevelopmental Psychiatry

Institute of Psychiatry, Psychology and Neuroscience, King's College London, UK. Email: rory.sheehan@kcl.ac.uk

Andre Strydom, MRCPsych, MSc, PhD, Professor in Intellectual Disabilities

Institute of Psychiatry, Psychology and Neuroscience, King's College London, UK. Email: andre.strydom@kcl.ac.uk

Umesh Chauhan, MBChB, MRCGP, MA, PhD, Professor of Primary Care

School of Medicine, University of Lancashire, Preston, UK. Email: uchauhan@uclan.ac.uk

Corresponding author: Umesh Chauhan, MBChB, MRCGP, MA, PhD, Professor of Primary Care

School of Medicine, University of Lancashire, Preston, UK. Email: uchauhan@uclan.ac.uk

Funding

LeDeR (Learning from Lives and Deaths: People with a learning disability and autistic people), which commissioned and approved this report and is funded by NHS England. For more details, see: https://leder.nhs.uk/about. The funders had no role in the analysis of data, preparation of this paper or decision to publish.

Ethical approval

LeDeR has approval from the UK Secretary of State for Health and Social Care to collect and analyse data compliant with Section 251 for the use of confidential patient information without consent for a specific purpose.

Competing Interests

The authors report no conflicts of interest.

Contributors

Umesh Chauhan contributed to **conceptualization**, **methodology**, and **supervision** of the study

Andre Strydom contributed to **conceptualization**, **methodology**, and **supervision** of the study

Rory Shehan contributed to **conceptualization**, **methodology**, and **supervision** of the study

For peer review only

For peer review only - http://bmjopen.bmj.com/site/about/guidelines.xhtml

Christina Roberts was responsible for **data curation**, **formal analysis** and drafted the **original manuscript**

Adam White supported formal analysis and editing the manuscript.

Alex Ruck- Keene and Nicola Ditzel were involved in **reviewing and editing** the manuscript.

Data Accessibility Statement

No additional data available.

Keywords

Resuscitation orders, DNR orders, do not resuscitate order, DNACPR, adult learning disabilities, end-of-life care.

For peer review only

Abstract

Objectives: Evidence suggests there are numerous issues with do not attempt cardiopulmonary resuscitation (DNACPR) recommendations for people with a learning disability. Recommendations are not always in place appropriately and sometimes DNACPR recommendations are not followed, though little is known about why or what happens in these cases. This paper aimed to explore what happens in cases where DNACPR is not followed correctly for people with a learning disability leading to undesired CPR.

Methods: Twenty-one reviews of deaths of people with a learning disability from an English national mortality programme in which DNACPR recommendations were not followed and a person received CPR were qualitatively synthesised using thematic analysis.

Findings: Through thematic analysis, several themes were identified which describe issues surrounding cases in which DNACPR recommendations are not followed. These are related to communication and documentation, the instinct to protect life, responding to an emergency, knowledge and awareness of DNACPR, and the decision-making process.

Conclusions: This study highlights the need for better training for care staff when responding to emergencies in which CPR would be considered, and better awareness of health and social care staff around procedures for locating documentation and alerting to a person's DNACPR status.

Strengths and limitations of this study

- Thematic analysis was completed with rigor with two researchers synthesising findings.
- Qualitative insights highlight key areas of improvement for practice around DNACPR recommendations.
- The data available may not represent all the deaths of people with a learning disability. Certain communities, for example, ethnic minorities, may be underrepresented.
- Secondary retrospective data meant that analysis was limited to second-hand accounts of events, potentially introducing recall and observer bias.

The data source did not include information about the appropriateness of DNACPR recommendations, which is an important consideration.

For peer review only

1. Introduction

Do Not Attempt Cardiopulmonary Resuscitation (DNACPR), also known as DNR (do not resuscitate) or DNAR (do not attempt resuscitation) guides healthcare professionals on whether to perform cardiopulmonary resuscitation (CPR) in the event of a cardiac or respiratory arrest.[1] DNACPR recommendations are intended to protect the individual from unnecessary suffering or loss of dignity in the dying process caused by receiving CPR, given CPR has a low success rate of between 11 to 18% in adults,[2] and can lead to adverse clinical outcomes such as brain damage and increased physical disability.[3]

These decisions should involve the person (if they have capacity) and or their family, and be recorded in medical records through forms like DNACPR Treatment Escalation Plan (TEPs) or a recommended summary plan for emergency care and treatment (ReSPECT).[1] Legally, patients must be included in discussions unless it would cause them harm, and clinicians must justify DNACPR decisions if patients wish for them.[4]

For people with a learning disability, DNACPR decision-making can be more complex due to communication barriers or lack of capacity.[5] In such cases, The Mental Capacity Act requires that legal proxies or those close to the person be involved, and decisions must follow a best interest approach.[6,7]

1.1. DNACPR recommendation decision-making for people with a learning disability

Challenges have been documented in end-of-life decision making, including making appropriate DNACPR recommendations, for people with a learning disability. These involve low levels of knowledge about people with a learning disability,[8] inappropriate assumptions about the quality of life, or a poorer prognosis for people with a learning disability.[9] Several other potential barriers to providing person-centered end-of-life care with appropriate DNACPR decision making were documented in the study, such as difficulties assessing pain or symptom severity in patients with limited communication, and a lack of understanding of the Mental Capacity Act.[7]

During COVID-19 pandemic, discriminatory practices emerged in how DNACPRs decisions were made, disproportionately impacting people with a learning disability. This group faced a COVID-19 death rate 4.1 times higher than the general population, highlighting existing health inequalities.[10] Concerns were raised over the use of 'blanket' DNACPR decisions, especially in care home settings where recommendations were applied broadly without individual assessment.[11] A United Kingdom (UK) study found that 55% of surveyed care home

For peer review only - http://bmjopen.bmj.com/site/about/guidelines.xhtml

professionals witnessed DNACPR forms added without consulting individuals or families, with 28% attributing this to blanket decisions based on frailty or disability.

In response to concerns about discriminatory DNACPR decisions, NHS England issued letters in 2020, 2021, and 2023, reminding health care providers that DNACPR orders must not be based solely on learning disability or autism. [13,14 ,15]

Despite these efforts, problematic clinical reasoning in DNACPR decisions persists. A 2024 report by the Parliamentary and Health Service Ombudsman identified ongoing issues, including ableism and ageism and recommended for improvements such as staff training, accessible communication for conversations for end-of-life decisions, updated guidance and improved record-keeping.[16]

1.2. Following DNACPR recommendations

Concerns persist around the appropriateness and implementation of DNACPR recommendations for people with a learning disability. Learning from Lives and Deaths of People with a learning disability and autistic people (LeDeR) reports (a National Health Service service improvement program aimed to improve health inequalities) indicate a rise in issues related to DNACPR processes with 64% of deaths involving such recommendations and increase in cases where these were not followed correctly – sometimes resulting in CPR being administered despite active DNACPR orders.[17, 18]

There is limited research specifically on why DNACPRs are not followed for people with a learning disability, though general population studies suggest factors like clinician override, poor communication, and lack of clarity about resuscitation contribute. [19, 20, 21] Misunderstanding of DNACPR meaning among healthcare professionals-such as believing it restricts other treatments-further complicates practice.[22]

Overall, several factors make DNACPR recommendations for people with a learning disability particularly complex and often mishandled. COVID-19 amplified existing health inequalities and unsafe practices, underscoring the urgent need for clearer guidance, training, and accountability. This paper explores the circumstances in which DNACPR recommendations were not followed, resulting in CPR by thematically analysing relevant death cases in England.

For peer review only

2. Method

2.1 Data source

This work uses data from LeDeR programme, a national mortality review in England which aims to address health inequalities for people with a learning disability and autistic people.

Deaths are reported to the programme via the LeDeR website and reviewed by trained professionals if the person had a confirmed learning disability and was a confirmed registered health services user in England. The reviews include both structured multiple-choice questions and detailed free-text narratives about the individual's life and death. For this study, particular attention was given to the free-text responses, especially where reviewers commented on issues related to DNACPR.

2.2. Sample selection

The sample of reviews within this was selected from a database containing all LeDeR reviews from 2020 to 2022. Specifically, it includes reviews for adults with a learning disability who died with a DNACPR recommendation in place that was not correctly followed. One question on the review was used as a proxy for this: "Was the DNACPR documentation correctly completed and followed?". Appendix 1 presents the potential multiple-choice responses to this question. This analysis only includes reviews from 2020 to 2022, as the question was not included prior to this.

2.3. Sample with DNACPR recommendations not followed correctly

Sixty-six reviews were initially identified by the researcher as not having had the DNACPR recommendations followed correctly. Starting from the most recent date of death, reviews were read in their entirety by one of the authors (CR) to ascertain the availability of information in the review about DNACPR. Cases in which DNACPR recommendations had not been followed resulting in CPR, with sufficient information in the review were retained for the final sample.

Twenty-one reviews had reviewer comments or reflections on issues with DNACPR recommendations not being followed, resulting in CPR, and were selected and included in the main sample for qualitative analysis. A second reviewer (AW) reviewed this final sample for rigour. The sample selection process is outlined in Figure 1.

For peer review only

2.4. Descriptive statistics

Descriptive statistics are gathered from LeDeR initial reviews. Sex, age at death, ethnicity and place of death are reported to give context to the qualitative findings.

2.5. Methodology

Following the approach to inductive thematic analysis,[23] six steps were undertaken to thematically analyse the reviews of deaths by two researchers (CR and AW). Free text, whereby reviewers indicated that DNACPR recommendations had not been followed and CPR had taken place were independently analysed. The authors followed the Standards for Reporting Qualitative Research (SRQR) guidelines.[24] The completed SRQR checklist is available as a supplementary file.

Table 1 shows the parts of the initial review which were read and coded by the researchers for information about issues with DNACPR recommendations being followed. All parts of the review considered by the researchers were free text boxes.

Table 1: Parts of the LeDeR initial review used by researchers to search for information about DNACPR adherence for the qualitative thematic analysis.

Review question/section	Information included here
**Summary of discussion with GP and/or review of GP notes**	Free text box where reviewers are asked to include key events of care and comment on aspects of care from the GP notes they feel are important.
**Conversation with another person**	This is a free text box summary of a discussion with another person and/or clinician involved in the care of the person who died.
**Pen Portrait**	The pen portrait is a free text box where reviewers summarize the person themselves, their preferences, important people to them and important life events.
**DNACPR comments "Please add any comments about the DNACPR recommendation"**	Free text box where reviewers can add comments about DNACPR.

For peer review only

**Issue, concern or potential problem (initial review)**	Free text box where reviewers extract learning from the review.
**Learning and actions: issues (focused review)**	As above.

Researcher reflection

Before data familiarisation and initial coding, both researchers kept a journal to document their initial thoughts, feelings, and assumptions towards the research subject. Reflective journaling was carried out throughout each stage of analysis, and they discussed their findings frequently. Through reflective discussion, the use of handwritten notes, and reflective journal entries, any differences were discussed and treated as a productive part of the research. Consensus was reached through these reflective discussions, leading to the generation of richer, more meaningful themes. Disagreements were resolved through the involvement of a third author (UC).

Data familiarisation

Data were compiled using Microsoft Excel. Both researchers independently read the reviews to immerse themselves in the data. This involved actively reading, reflecting, and re-reading the reviews several times.

Generating codes

A flexible and iterative process was undertaken. Initial codes were generated from the data, primarily completed by one researcher (CR). Reflective discussions were then carried out with the second researcher (AW). The codes represent underlying themes within the data, not just descriptively, but also in terms of their meaning. An example of an initial code “Information sharing” is presented below.

For peer review only

Table 2: Example of the coding process from the six stages of thematic analysis.

Extract	Initial code
Reviewer 9: "Treatment Escalation Plan was not able to be found by night staff"<br><br>Reviewer 17: "When she discovered that patient had been resuscitated because staff where not aware of the DNA she felt this was extremely sad and that the manager should have ensured all staff were aware of decisions."<br><br>Reviewer 18: "CPR was commenced as records viewed by staff did not reflect an alert that she had a DNACPR in place"<br><br>Reviewer 19: "It is reported by Sister that she was upset that resuscitation was being commence as the DNA CPR was not known to the ambulance service"<br><br>Reviewer 20: "There was a respect form in place however there was a lot of confusion around this. It had not been clearly communicated with mother or care team."	Lack of staff awareness of DNACPR in place

For peer review only

Developing themes

This process involved reviewing the initial themes generated during the coding stage and considering how they relate to one another, overlap and repeat. Iterative coding was conducted using mind maps on paper to refine the codes into groups of themes. These then became candidates for mature themes in the data. Analysis continued until no new themes emerged which indicated saturation point.

Refining mature themes

Mind maps were used to group, ungroup and regroup early themes into mature themes. This enabled the researchers to visualise the best fit for the data. After reviewing the mature themes, the researchers discussed how to best name each theme in a way that captures the essence of the theme, while also considering how to effectively describe each theme and the issues they represent.

Producing the analysis

The final analysis and results section below aims to be clear, concise and useful. The researchers have selected several quotes or notes from the free text. These were taken at various stages of the coding and theme generation process. Each individual quote provides valuable context to the complexity of the issues the theme represents. Any names included in the quotes have been removed to prevent identification.

2.6. Patient and public involvement

None

3. Results

3.1. Demographics

The demographics of the 21 adults with a learning disability whose deaths were reviewed by LeDeR and judged by reviewers to have had DNACPR followed incorrectly are shown in Table 3. The average age at death (mean) of the sample was 64.7 years.

Table 3: Descriptive statistics of the demographics of the sample of 21 adults with a learning disability whose deaths were reviewed by LeDeR and judged by reviewers to have had DNACPR followed incorrectly.

Demographic	N (%)
**Sex**	
Male	8 (38%)
Female	13 (62%)
**Ethnicity**	
White	20 (95%)

For peer review only

**Place of death**	
Hospital	8 (38%)
Usual residence	11 (52%)
**Year of death**	
2021	8 (38%)
2022	13 (62%)

3.2. Overarching themes

Two overarching themes and three mature themes were consolidated from the reviews that represent key issues when following DNACPR recommendations. The overarching themes were Communication and documentation and Instinct to protect life. The three main themes were: Responding to an emergency, Knowledge and awareness of DNACPR, and the decision-making process. A thematic map of the data is shown in Figure 2.

3.2.1. Communication and documentation

The first of the two overarching themes is Communication and documentation. Communication in general was found to be a common thread which weaved through all four main themes.

Information sharing for care professionals involved in the communication around DNACPR recommendations between different health and social care systems was a key aspect of communication. Communication is especially important when supporting the understanding of DNACPR and plans around emergency care. Breakdowns in communication often led to a lack of awareness or knowledge of the recommendations, and when faced with emergency situations, CPR would commence as a default.

“It is reported by Sister that she was upset that resuscitation was being commenced as the DNA CPR was not known to the ambulance service” - *Reviewer 19*

Documentation helps to communicate DNACPR recommendations between care pathways, and problems with documentation can present difficulties for clinicians when considering resuscitation options. Communication extends to the decision-making process. Difficulties with communicating the process could lead to confusion and ultimately doubt over the validity or appropriateness of DNACPR recommendations.

“There was a ReSPECT form in place however there was a lot of confusion around this. It had not been clearly communicated with mother or care team.” - *Reviewer 20*

Open channels of communication can help to prevent issues when following DNACPR recommendations, ensuring that DNACPR recommendations are appropriate. Ensuring all parties involved in the health and care of the person are aware of the recommendation, and

For peer review only

have easy access to the correctly completed documentation, is key to ensuring wishes around DNACPR can be followed.

Documenting the recommendation, which captures the manifold issues described by reviewers with relevant documentation, including DNACPR forms, ReSPECT forms and TEP forms. Two main issues emerged with documentation. The first was the availability of the documentation, with some reviewers describing situations in which documentation could not be found. Unavailable documentation often led to relevant professionals being unaware of the recommendation or unable to follow the recommendation without documented evidence, leading to CPR being commenced.

“Staff were unable to locate DNACPR/ReSPECT form although they knew [Name]was not to be resuscitated. Arrest trolley called for.” - Reviewer 5

“Carers called emergency services and carried out CPR for approximately 10 minutes until they located the TEP and then ceased.” - Reviewer 9

Some reviewers described situations in which sections of the DNACPR form or other documentation were incomplete or incorrect. Sometimes documentation was not signed off correctly, or sections were missing on the form. Rightfully, sometimes professionals questioned the validity of the recommendation when presented with incomplete or incorrect documentation, leading to CPR being commenced.

“It does not appear that the GP signed off [Name]’s emergency health care plan or advance care plans, nor did they attend meetings where these were formulated.” - Reviewer 12

In other cases, the validity of the DNACPR documentation was questioned due to the available documented decision being “old”. This led to confusion about how to proceed and distress for those involved in the situation.

“Ambulance log states DNACPR in place staff stated, " It is not valid as it is old". Recorded that valid DNACPR had been viewed.” - Reviewer 13

3.2.2. Instinct to protect life

The second major theme identified was Instinct to protect life, reflecting the compassion and commitment of family, carers, health and social care staff to safeguard those they care for. In situations where a person's DNACPR status was unclear, missing, or disputed, responders often defaulted to performing CPR—especially during emergencies like 999 calls—believing it was the safest and most responsible action. The instinct to protect and act appropriately on behalf of a person with a learning disability who is unwell is evident in decision-making processes.

For peer review only

This instinct was particularly strong among those with close personal or caregiving relationships, making it emotionally difficult to withhold resuscitation. The complexity of end-of-life decisions and lack of consensus could lead to confusion, distress, and even trauma for those involved. Ultimately, this theme highlights the emotional burden and ethical tension in following DNACPR recommendations and the critical need for clear communication, planning, and support—especially when caring for people with learning disabilities.

3.3. Mature themes

3.3.1. Responding to an emergency

The first theme represents the challenges faced when responding to an emergency. Often emergencies were sudden, unexpected and took paid carers or health professionals by surprise. Several of the reviews commented on the sudden nature of the person's collapse, and how those around them responded instinctively and called for help from emergency services. It was emergency services involvement that sometimes triggered CPR to be commenced, as those attending to the collapse would "follow instructions" from the call handlers.

"Staff were adrenaline fuelled in an emergency situation and followed 999 call handler's instructions" - Reviewer 1

CPR attempts were sometimes ceased once paramedics arrived and could access medical records and verify a person's DNACPR status. However, this information was not always readily accessible in residential or non-medical settings, leading to confusion and distress about how to proceed.

"After paramedic obtained medical advice they found records stated person was not for resuscitation and attempts stopped" - Reviewer 5

A particularly striking example involved a person who collapsed suddenly in an accident. Despite having a DNACPR in place, the unusual circumstances prompted a reversal of the decision. The correct course of action was to attempt to save the person's life, given that the events were not related to the natural progression of their condition.

"It was recognised by the staff who found [Name] that she had a DNACPR, they phoned the on-call director who advised them to start CPR given the circumstances she had been found in." - Reviewer 15

These cases reveal not only the procedural challenges of emergency response but also point to deeper structural issues. For people with a learning disability, inconsistencies in access to healthcare, poor communication across services, and a lack of training for frontline responders can compound the risk of inappropriate or delayed care.

For peer review only

3.3.2. Knowledge and awareness of DNACPR

The second theme is around the knowledge and awareness of DNACPR among those involved in caring for the person with a learning disability. Issues around knowledge and awareness of DNACPR were on different levels.

Firstly, there is the issue of people being unaware of a DNACPR recommendation being in place at all. Being unaware of an active, valid DNACPR meant those attending to the collapsed patient proceeded in the natural and appropriate way by considering resuscitation.

"Staff denied any knowledge of a DNACPR" - Reviewer 17

The reasons for a lack of knowledge and awareness of a DNACPR recommendation often relate to communication issues between the clinicians involved in the decision-making and others involved in their care. However, often communication around DNACPR status was hindered by alerting systems not working as they should. For example, in a hospital, a patient with a blank bed board and no wristband meant staff were not aware of DNACPR status. Other reasons for a lack of knowledge and awareness of a DNACPR recommendation include issues with documentation.

"CPR was commenced as records viewed by staff did not reflect an alert that she had a DNACPR in place, during the efforts to resuscitate her it was noted that the alert was now evident on her records so CPR stopped" - Reviewer 18

Secondly, in some cases, individuals caring for the person with a learning disability struggled to understand what a DNACPR recommendation means in practice, so that when the situation of a collapse presented itself, they called emergency services and followed instructions for CPR. Some reviewers commented on the distress this confusion caused for the people involved in the situation, and some examples highlighted the "panic" and "upset" felt, particularly by care home staff who did not feel confident in knowing what to do when someone collapses with a DNACPR recommendation in place.

"CPR was commenced following instruction from 999. There was a ReSPECT form in place however there was a lot of confusion around this. It had not been clearly communicated with mother or care team." - Reviewer 20

3.3.3. The decision-making process

The final theme relates to issues with the decision-making process around DNACPR and concerns or disagreements about how the recommendation was made. Given the gravity of DNACPR recommendations, different opinions about how to proceed in the best interests of

For peer review only

the person with a learning disability are to be expected. One reviewer described a situation in which a person's family had agreed with a DNACPR recommendation, but the recommendation was changed following objections from the care team. This example also demonstrates the issue of DNACPR being used as a proxy for no treatment.

"The DNACPR completed at that time in discussion with her family advised no treatment due to her poor quality of life. This decision was changed following objections from her care team."- Reviewer 15

In some cases, issues with the decision-making process centered around considering the wishes and feelings of the person with a learning disability. One example was given of a person who did not want a DNACPR to be put in place, but clinicians disagreed and recommended DNACPR. This led to DNACPR documentation being incomplete at the time of the person's collapse and a distressing situation for carers. Prolonged and difficult decision-making processes around DNACPR led to incomplete or confusing documentation and distress for those involved in the process. It was also noted that the Care Home paperwork (Covid-19 Hospital Summary) recorded that the patient said *"Doctors have previously put DNAR in place without asking me. I have made it clear that I want to be resuscitated" - Reviewer 1.*

4. Discussion

This paper examined cases were DNACPR recommendations for people with a learning disability were not followed, resulting in CPR being administered. Through qualitative thematic analysis of LeDeR review data, the study identified two central themes: communication failures and the instinct to protect life, which often led to CPR being attempted despite active DNACPR orders. Three specific themes emerged: emergency response actions, awareness and understanding of DNACPR, and the decision-making process. Poor communication - both in conveying DNACPR status and during decision-making - was a key factor in these failures. Many professionals defaulted to life-saving interventions due to uncertainty or lack of clear guidance, and perhaps the complex ethical dilemma they are faced with when considering how to respond to an emergency situation. Communication issues underpinned many of the cases in which DNACPR recommendations were not followed. These issues ranged from communicating the DNACPR recommendation itself, to communication issues between individuals involved in the decision-making process. To improve practice around DNACPR recommendations for people with a learning disability, facilitating better communication is key. This involves communication between professionals, family members and the person with a learning disability around DNACPR status, as well as improving communication during the decision-making process for people with a learning disability both with and without capacity.

Despite its relevance, the Mental Capacity Act,[7] was rarely mentioned in reviewer comments, perhaps due to the limitations in the structure of the reviews themselves, which do not prompt formal reflection on the Mental Capacity Act. Failure to embed Mental Capacity Act principles may not only lead to inappropriate care but also reinforce structural inequalities for people with a learning disability, particularly where assumptions are made about their ability to engage in these sensitive discussions.

Previous research has indicated that misunderstanding of DNACPR recommendations is common even amongst nursing professionals working in elderly adult care,[22] and although this came through in the theme 'Knowledge and awareness of DNACPR', the data was not suitable for looking in detail at levels of understanding of DNACPR by professionals caring for people with a learning disability. Understanding and knowledge issues are potentially key barriers to both following DNACPR recommendations and ensuring recommendations are appropriate. More work is needed to explore how health and social care professionals implement DNACPR recommendations and how this may impact upon procedures being followed correctly.

A strength of this paper is that it provides rich qualitative findings which give insight into the issues surrounding cases in which DNACPR recommendations are not followed for people with a learning disability, leading to CPR taking place. A rigorous qualitative approach was undertaken which identified key areas of improvement for practice around DNACPR recommendations.

As we used retrospective secondary data (reviewers' comments about DNACPR), analysis was limited by the details that reviewers chose to share about the events surrounding a death. The data source meant that the researchers did not have access to the DNACPR documentation and did not have firsthand accounts of the events surrounding situations in which CPR was considered. Although reviewers are experienced professionals who receive extensive training, it is likely the analysis does not capture a full evaluation of the circumstances surrounding DNACPR and may be subject to bias. This meant that the data source was not suitable for looking in detail at important aspects of the DNACPR decision-making process itself.

5. Conclusion

This work highlights the need for greater awareness and training among health and social care staff around procedures for locating end-of-life planning documentation, which alerts to a person's DNACPR status.

For peer review only

6. Recommendations

To improve DNACPR practice, the paper recommends: Ensuring capacity assessments are decision-specific and revisited as needed. Involving individuals lacking capacity using accessible methods and support from familiar people. Making best interest decisions that reflect the person's values and involve all relevant parties. Training staff supporting people with a learning disability in how to use and locate relevant documentation relating to DNACPR.

Future research should monitor the extent to which DNACPR recommendations are in place appropriately for people with a learning disability, to assess whether practices around DNACPR are improving in line with recent recommendations (Parliamentary and Health Ombudsman, 2024). An interesting line for future work would be to investigate how the Mental Capacity Act (Department of Health, 2005) is being used in the DNACPR decision-making process.

For peer review only

References

472
1. National Health Service. Do Not Attempt Cardiopulmonary Resuscitation (DNACPR) Decisions [Internet]. NHS. 2021. Available from: https://www.nhs.uk/conditions/do-not-attempt-cardiopulmonary-resuscitation-dnacpr-decisions/

2. van Gijn MS, Frijns D, van de Glind EMM, C. van Munster B, Hamaker ME. The chance of survival and the functional outcome after in-hospital cardiopulmonary resuscitation in older people: a systematic review. Age and Ageing. 2014 Apr 22;43(4):456–63.

3. General Medical Council. Cardiopulmonary resuscitation (CPR) [Internet]. Gmc-uk.org. 2024. Available from: https://www.gmc-uk.org/professional-standards/the-professional-standards/treatment-and-care-towards-the-end-of-life/cardiopulmonary-resuscitation-cpr

4. Tracy Vs Cambridge University Hospitals. NHS Foundation Trust [2014]. ECWA Civ 33 and Anor [Internet]. Available from: https://www.judiciary.uk/wp-content/uploads/2014/06/tracey-approved.pdf

5. Wagemans AMA, van Schrojenstein Lantman-de Valk HMJ, Proot IM, Bressers AM, Metsemakers J, Tuffrey-Wijne I, et al. Do-Not-Attempt-Resuscitation orders for people with intellectual disabilities: dilemmas and uncertainties for ID physicians and trainees. The importance of the deliberation process. Journal of Intellectual Disability Research. 2016 Aug 26;61(3):245–54.

6. Winspear v City Hospitals Sunderland NHS Foundation Trust [2015] EWHC 3250 (QB) (13 November 2015) [Internet]. Bailii.org. 2015. Available from: https://www.bailii.org/ew/cases/EWHC/QB/2015/3250.html

7. Mental Capacity Act. Mental Capacity Act 2005 [Internet]. Legislation.gov.uk. Gov.uk; 2005. Available from: https://www.legislation.gov.uk/ukpga/2005/9/contents

8. Stein GL. Providing Palliative Care to People with Intellectual Disabilities: Services, Staff Knowledge, and Challenges. Journal of Palliative Medicine. 2008 Nov;11(9):1241–8.

9. Tuffrey-Wijne I, Goulding L, Gordon V, Abraham E, Giatras N, Edwards C, et al. The challenges in monitoring and preventing patient safety incidents for people with intellectual disabilities in NHS acute hospitals: evidence from a mixed-methods study. BMC Health Services Research. 2014 Sep 24;14(1).

10. COVID-19: deaths of people with learning disabilities [Internet]. GOV.UK. Available from: https://www.gov.uk/government/publications/covid-19-deaths-of-people-with-learning-disabilities

For peer review only

11. Tapper J. Fury at "do not resuscitate" notices given to Covid patients with learning disabilities [Internet]. The Guardian. 2021. Available from: https://www.theguardian.com/world/2021/feb/13/new-do-not-resuscitate-orders-imposed-on-covid-19-patients-with-learning-difficulties

12. Fitton E, Kuylen M, Wyllie A, Michalowski S, Bhatt V, Martin W. Managing DNACPR Recommendations in Residential Care: Towards Improved Training for Social Care and Capacity Professionals. British Journal of Social Work. 2023 May 16;53(5).

13. Murdoch C, Banks R, Kanani N. Coronavirus» Do Not Attempt Cardiopulmonary Resuscitation DNACPR letter 3 April 2020 [Internet]. England.nhs.uk. 2020 [cited 2025 May 6]. Available from: https://www.england.nhs.uk/coronavirus/documents/do-not-attempt-cardiopulmonary-resuscitation-dnacpr-letter-3-april-2020/

14. Powis S, May R, Kanani N. Coronavirus» Do Not Attempt Cardiopulmonary Resuscitation (DNACPR) and people with a learning disability and or autism [Internet]. www.england.nhs.uk. 2021. Available from: https://www.england.nhs.uk/coronavirus/documents/c1146-do-not-attempt-cardiopulmonary-resuscitation-dnacpr-and-people-with-a-learning-disability-and-or-autism/

15. Powis S, Doyle A, Wee B, Banks R, May R. NHS England» Do not attempt cardiopulmonary resuscitation (DNACPR) and people with a learning disability and or autism [Internet]. England.nhs.uk. 2023 [cited 2025 May 6]. Available from: https://www.england.nhs.uk/publication/dnacpr-and-people-with-a-learning-disability-and-or-autism/

16. End of life care: improving DNACPR conversations for everyone | Parliamentary and Health Service Ombudsman (PHSO) [Internet]. www.ombudsman.org.uk. 2024. Available from: https://www.ombudsman.org.uk/publications/end-life-care-improving-dnacpr-conversations-everyone

17. White A, Sheehan R, Ding J, Roberts C, Magill N, Keagan-Bull R, et al. Learning from Lives and Deaths - People with a learning disability and autistic people (LeDeR) report for 2021 [Internet]. UCLan - University of Central Lancashire. 2022. Available from: https://clok.uclan.ac.uk/43444/?template=default_internal

18. White A, Sheehan R, Ding J, Roberts C, Magill N, Keagan-Bull R, et al. LeDeR Annual Report Learning from Lives and Deaths: People with a Learning Disability and Autistic People [Internet]. 2022. Available from: https://www.kcl.ac.uk/ioppn/assets/fans-dept/leder-2022-v2.0.pdf

19. Wong J, Duane PG, Ingraham NE. A case series of patients who were do not resuscitate but underwent cardiopulmonary resuscitation. Resuscitation. 2020 Jan;146:145–6.

For peer review only

20. O'Brien H, Scarlett S, Brady A, Harkin K, Kenny RA, Moriarty J. Do-not-attempt-resuscitation (DNAR) orders: understanding and interpretation of their use in the hospitalised patient in Ireland. A brief report. Journal of Medical Ethics. 2017 Nov 3;44(3):201–3.

21. Robinson F, Cupples M, Corrigan M. Implementing a resuscitation policy for patients at the end of life in an acute hospital setting: qualitative study. Palliative Medicine. 2007 Jun;21(4):305–12.

22. Nolan J, Robertson T, Donaldson J. A Mixed-Methods Study of DNACPR in Nursing Practice In partial fulfilment of the degree of Clinical Doctorate in Nursing [Internet]. 2022. Available from: https://dspace.stir.ac.uk/bitstream/1893/35023/1/A%20Mixed-Methods%20Study%20of%20DNACPR%20in%20Nursing%20Practice.pdf

23. Braun V, Clarke V. One Size Fits all? What Counts as Quality Practice in (reflexive) Thematic analysis? Qualitative Research in Psychology. 2020 Aug 12;18(3):328–52.

24. O'Brien BC, Harris IB, Beckman TJ, Reed DA, Cook DA. Standards for Reporting Qualitative Research. Academic Medicine [Internet]. 2014 Sep;89(9):1245–51. Available from: https://journals.lww.com/academicmedicine/fulltext/2014/09000/standards_for_reporting_qualitative_research__a.21.aspx

For peer review only

Appendix

Appendix 1: Reviewer judgment of whether DNACPR documentation was correctly completed or followed for reviews of deaths in 2020 to 2022 of people with a learning disability with a DNACPR recommendation in place.

Was the DNACPR documentation correctly completed and followed?	2020	2021	2022	Total
**Correctly Completed and Followed**	52.0% (n = 92)	60.9% (n = 1,144)	63.3% (n = 979)	61.5% (n = 2,215)
**Correctly Completed but Was Not Followed**	*	1.1% (n = 21)	1.2% (n = 19)	*
**Incorrectly Completed and Followed**	11.9% (n = 21)	6.2% (n = 116)	7.1% (n = 109)	6.8% (n = 246)
**Neither Completed nor Followed Correctly**	*	0.6% (n = 11)	0.8% (n = 13)	*
**Don't know**	35.0% (n = 62)	31.2% (n = 585)	27.6% (n = 426)	29.8% (n = 1,073)
**Total**	177	1,877	1,546	3,600

For peer review only

Figure 1: A flowchart of the selection process for the sample of reviews in which DNACPR recommendations were not followed, resulting in CPR.

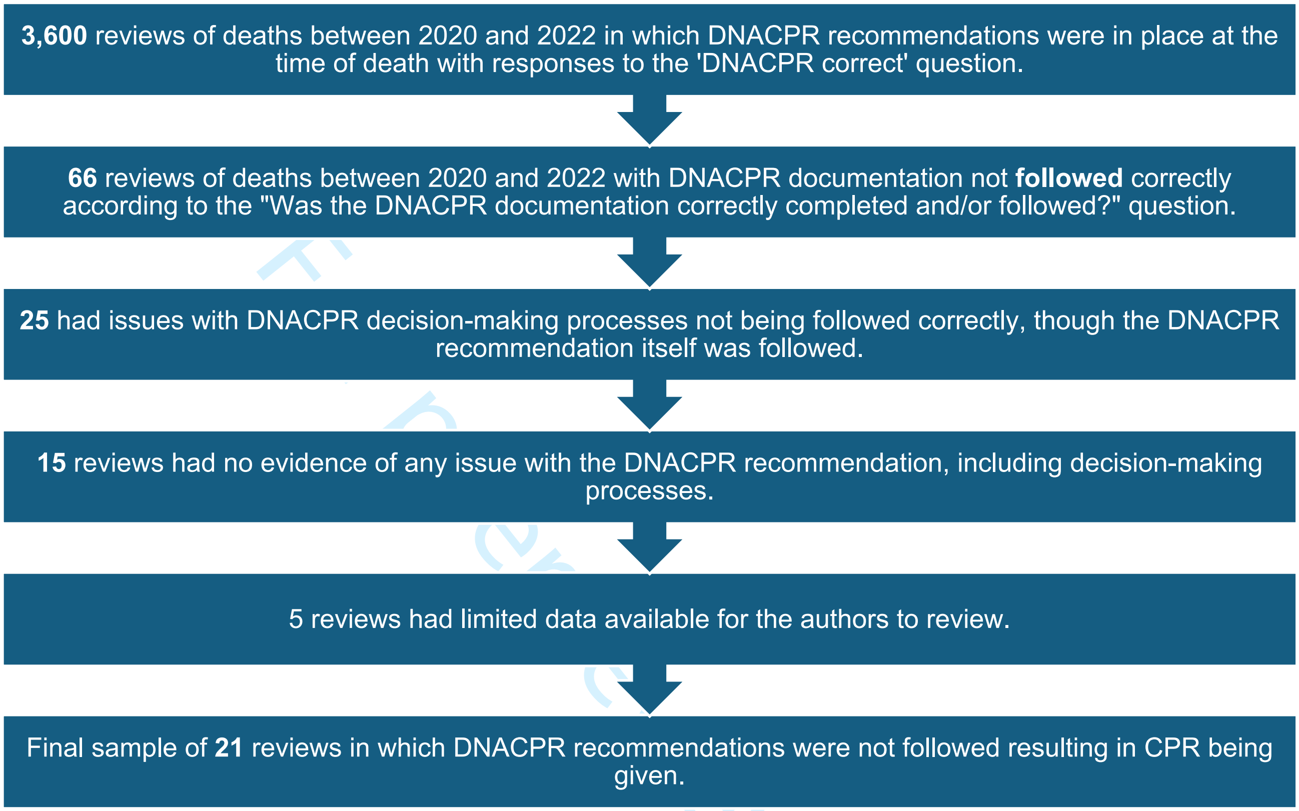

For peer review only

563 *Figure 2: Final thematic map of the data from reviews in which reviewers indicated DNACPR*
564 *documentation was not followed leading to a person receiving CPR.*

565

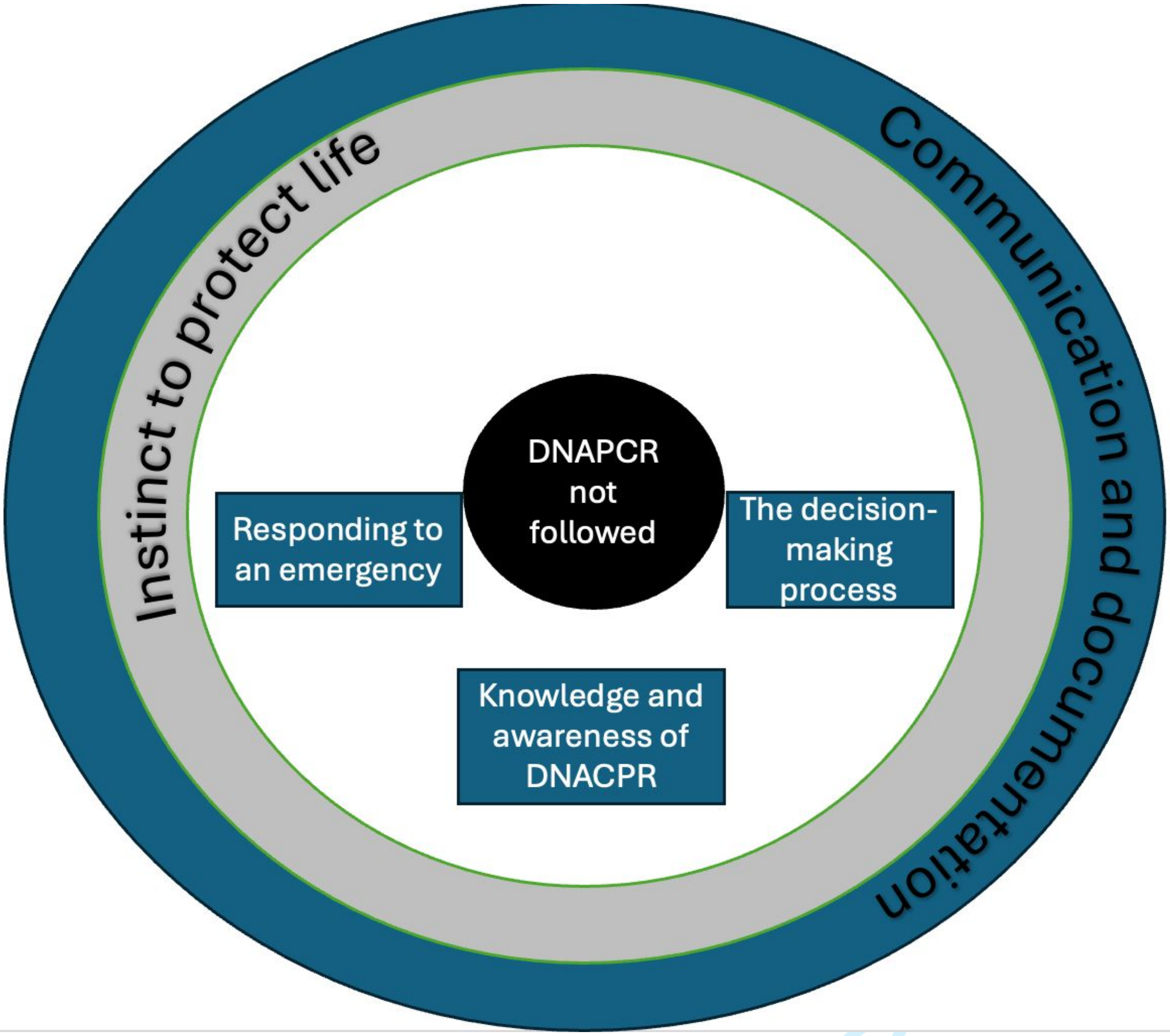

566

BMJ Open

“An adrenaline-fueled emergency”: a qualitative thematic analysis of reviews of deaths in England where DNACPR (Do Not Attempt Cardiopulmonary Resuscitation) recommendations are not implemented for people with a learning disability

Journal:	*BMJ Open*
Manuscript ID	bmjopen-2025-107222.R1
Article Type:	Original research
Date Submitted by the Author:	21-Apr-2026
Complete List of Authors:	Roberts, Christina; Kingston University, Faculty of Health, Science, Social Care and Education<br>Ditzel, Nicola; UCLan, ; King's College London,<br>White, Adam; King's College London Institute of Psychiatry Psychology & Neuroscience, Forensic and Neurodevelopmental Sciences<br>Sheehan, Rory; King's College London Institute of Psychiatry Psychology & Neuroscience, Forensic and Neurodevelopmental Sciences<br>Strydom, André; Institute of Psychiatry Psychology and Neuroscience, ; King's College London<br>Ruck-Keene, Alex; King's College London, Institute of Psychiatry, Psychology and Neuroscience<br>Chauhan, Umesh; University of Central Lancashire, School of Health
**Primary Subject Heading**:	Emergency medicine
Secondary Subject Heading:	Qualitative research
Keywords:	Disabled Persons, Adult palliative care < PALLIATIVE CARE, Cardiopulmonary Resuscitation

SCHOLARONE MANUSCRIPTS

I, the Submitting Author has the right to grant and does grant on behalf of all authors of the Work (as defined in the below author licence), an exclusive licence and/or a non-exclusive licence for contributions from authors who are: i) UK Crown employees; ii) where BMJ has agreed a CC-BY licence shall apply, and/or iii) in accordance with the terms applicable for US Federal Government officers or employees acting as part of their official duties; on a worldwide, perpetual, irrevocable, royalty-free basis to BMJ Publishing Group Ltd ("BMJ") its licensees and where the relevant Journal is co-owned by BMJ to the co-owners of the Journal, to publish the Work in this journal and any other BMJ products and to exploit all rights, as set out in our licence.

The Submitting Author accepts and understands that any supply made under these terms is made by BMJ to the Submitting Author unless you are acting as an employee on behalf of your employer or a postgraduate student of an affiliated institution which is paying any applicable article publishing charge ("APC") for Open Access articles. Where the Submitting Author wishes to make the Work available on an Open Access basis (and intends to pay the relevant APC), the terms of reuse of such Open Access shall be governed by a Creative Commons licence – details of these licences and which Creative Commons licence will apply to this Work are set out in our licence referred to above.

Other than as permitted in any relevant BMJ Author's Self Archiving Policies, I confirm this Work has not been accepted for publication elsewhere, is not being considered for publication elsewhere and does not duplicate material already published. I confirm all authors consent to publication of this Work and authorise the granting of this licence.

For peer review only

"An adrenaline-fueled emergency": a qualitative thematic analysis of reviews of deaths in England where DNACPR (Do Not Attempt Cardiopulmonary Resuscitation) recommendations are not implemented for people with a learning disability

Abstract

Objectives: To investigate the circumstances and contributory factors in cases where cardiopulmonary resuscitation (CPR) is administered to people with a learning disability despite a do-not-attempt cardiopulmonary resuscitation (DNACPR) recommendation being in place. Limited understanding exists around why DNACPR recommendations are sometimes not followed in practice. This study aims to address this gap by examining what occurs when CPR is performed contrary to an established DNACPR recommendation.

Design: Qualitative study using inductive thematic analysis of secondary data derived from comprehensive reviews of deaths of people with a learning disability.

Setting: England, deaths occurring in hospital or out-of-hospital.

Participants: Twenty-one adults (aged ≥18 years) with a learning disability who died between 2020 and 2022 and whose deaths were reviewed through the Learning from Lives and Deaths (LeDeR) programme. Cases were included where a DNACPR recommendation was in place but not followed

Results: Three themes describe pressures to respond to an emergency, knowledge and awareness of DNACPR, and decision-making processes. Two cross-cutting factors shaped how DNACPR recommendations were enacted in practice: breakdowns in information sharing and unclear or inaccessible documentation. The challenges described in each theme, compounded by the cross-cutting factors, created uncertainty and contributed to a default to life-preserving action despite active DNACPR recommendations being in place.

Conclusions: DNACPR recommendations for people with a learning disability are at risk of not being followed as intended in emergency contexts due to challenges in communication and documentation. These issues create uncertainty and may contribute to a default to attempt resuscitation. Improvements are needed in how DNACPR decisions are documented, shared and accessed across care settings, alongside training to support staff in interpreting and

For peer review only

applying recommendations in complex situations, including within the legal framework of the Mental Capacity Act (2005).

Strengths and limitations of this study

- Three researchers collaboratively undertook the thematic analysis, enhancing the rigour of the findings.
- Reflective qualitative insights highlight key areas of improvement for practice related to DNACPR recommendations.
- The dataset may not represent certain communities, for example, people with a learning disability from an ethnic minority group.
- The use of secondary retrospective data meant that analysis was limited to second-hand accounts of events, potentially introducing recall and observer bias.
- The data source did not include sufficient detail to assess the appropriateness of DNACPR recommendations, and evaluation of the quality of the original DNACPR decision is beyond the scope of this paper.

Keywords

Resuscitation orders, DNR orders, do not resuscitate order, DNACPR, learning disability, hospital care, end-of-life care.

INTRODUCTION

Do Not Attempt Cardiopulmonary Resuscitation (DNACPR), also known as DNR (do not resuscitate) or DNAR (do not attempt resuscitation), instructs healthcare professionals whether to perform cardiopulmonary resuscitation (CPR) in the event of a cardiac or respiratory arrest [1]. DNACPR recommendations are intended to protect the individual from unnecessary suffering or loss of dignity in the dying process caused by receiving CPR, given that CPR has a low success rate of between 11 to 18% in adults [2] and can lead to adverse clinical outcomes such as brain damage and increased physical disability[3].

DNACPR decision-making is ethically complex and inconsistently implemented [5]. Case law has confirmed that patients must be included in discussions unless it would cause them harm. The decision must be clearly recorded in medical records through specific forms including Treatment Escalation Plan (TEPs), or a Recommended Summary Plan for Emergency Care and

For peer review only

Treatment (ReSPECT) [1]. TEPs are clinical communication tools which can be used to document a patient's preferences and medical recommendations for care and similarly ReSPECT is a personalised, non-legally binding document outlining a person's preferences for care, including CPR decisions, if they become critically ill or incapacitated. Clinicians must be able to justify DNACPR decisions that conflict with a patient's wishes [4].

DNACPR recommendations vary in how they are made, recorded and communicated across settings, with ongoing concerns about how patients are involved and whether staff understand the implications of an active recommendation [5, 6].

DNACPR decision-making for people with a learning disability

For people with a learning disability, DNACPR decision-making can be more complex due to communication barriers or lack of capacity [7]. In such cases, The Mental Capactiy Act (2005) requires that legal proxies or those close to the person be involved, and decisions must follow a best interest framework [8,9]. Many of the challenges identified in DNACPR decision-making for people with a learning disability reflect broader systemic issues documented in the general population [5], but these may be amplified due to structural inequalities and diagnostic overshadowing (where symptoms are attributed to a person's learning disability rather than recognised as a separate health condition).

Concerns have been documented relating to end-of-life decision-making for people with a learning disability, including making appropriate DNACPR recommendations. These involve low levels of knowledge about the specific needs of people with a learning disability [10], inappropriate assumptions about their quality of life, and recognising end-of-life care needs for people with a learning disability [11]. Previous research indicates barriers to providing good end-of-life care with appropriate DNACPR discussions include difficulties assessing pain or symptom severity in patients with limited communication, and a lack of understanding of the Mental Capacity Act [9].

During the COVID-19 pandemic, discriminatory DNACPR practices disproportionately impacted people with a learning disability, which may have contributed to individuals with learning disability having a COVID-19 death rate > 4 times higher than the general population [12]. National concerns were raised about the use of 'blanket' DNACPR decisions, where recommendations were applied broadly, without individual assessment or consultation with individuals or families [13-14].

In response to these concerns, the National Health Service (NHS) in England issued letters in 2020, 2021, and 2023, reminding health care providers that DNACPR orders must not be based solely on the presence of a learning disability or autism [15-17].

For peer review only

Despite these efforts, problematic clinical reasoning in DNACPR decisions persist in England. A 2024 report by the Parliamentary and Health Service Ombudsman, which examined DNACPR use in the general population, identified ongoing issues including ableism and ageism. The report recommended several improvements including staff training, accessible communication for conversations around end-of-life decisions, updated guidance, and improved record-keeping [18].

Following DNACPR recommendations

Concerns persist around the implementation of DNACPR recommendations for people with a learning disability. 'Learning from lives and deaths - people with a learning disability and autistic people' (LeDeR, an NHS England improvement initiative aimed to reduce health inequalities) annual reports indicate a rise in issues related to DNACPR processes during the pandemic. Almost two-thirds of deaths of people with a learning disability involve DNACPRs, yet the recommendations were assessed to have not been followed correctly in a significant proportion of cases [19-20].

There is limited research specifically on why DNACPRs are not followed for people with a learning disability, though studies conducted with general population samples suggest factors like clinician override, poor communication, and lack of clarity about the requirements of DNACPR may contribute[21-23]. Misunderstanding of DNACPR meaning among healthcare professionals, such as believing it extends to the withdrawal of other treatments or interventions, further complicates practice [24].

This paper aims to explore the circumstances in which DNACPR recommendations were not followed, resulting in CPR being administered to people with a learning disability who subsequently died, through thematic analysis of relevant cases in England.

METHODS

Data source

This work uses data from 'Learning from lives and deaths – people with a learning disability and autistic people', a national mortality review in England which aims to address health inequalities.

Deaths are reported to LeDeR via a website and reviewed to confirm the person had a learning disability and registered as a health services user in England. A trained reviewer completes a structured review of the death, including consultation with those involved in care and a review of records. Reviews include multiple-choice and free-text narratives. For this study, particular

For peer review only - http://bmjopen.bmj.com/site/about/guidelines.xhtml

attention was given to the free-text responses, especially where reviewers commented on issues related to DNACPR. All reviews are anonymised prior to data being transferred to the academic team for analysis. Although reviewers follow guidance, the free text information written in reviews is based on an individual reviewers' interpretation of the person's care, treatment, and the circumstances surrounding the death,

Sample selection

The sample was drawn from a database containing all LeDeR reviews of people with a learning disability who died between 2020 to 2022 and had responses to the question: "Was the DNACPR documentation correctly completed and followed" (n = 3600). Supplementary Table 1 presents the potential multiple-choice responses to this question.

Sample with DNACPR recommendations not followed correctly

Sixty-six reviews of the 3600 reviews were identified from response categories indicating that DNACPR recommendations were in place but some aspect of the DNACPR (either making the decision or carrying out the recommendation) not been followed correctly. These included cases where DNACPR documentation was recorded as 'correctly completed but not followed' (n = 42), and 'neither completed nor followed correctly' (n = 24). Starting from the most recent date of death, reviews were read in their entirety by one of the authors (CR) to ascertain the availability of information in the review about DNACPR.

Following full review, cases were excluded where:
(1) DNACPR recommendations themselves were followed despite issues in the decision-making process (n = 25);
(2) there was no further detail in the free text within the review of a DNACPR-related issue (n = 15); or
(3) there was insufficient detail in the review to analyse the circumstances surrounding DNACPR (n = 5).

Twenty-one reviews were included for qualitative analysis

Descriptive statistics

Descriptive statistics were gathered from LeDeR reviews. Sex, age at death, ethnicity and place of death are reported to give context to the qualitative findings.

Methodology

This study is underpinned by an interpretivist approach, recognising that meaning is constructed through subjective interpretation of experiences and accounts. Following the approach to inductive thematic analysis [25] six steps were undertaken to thematically analyse the reviews of deaths by three researchers (CR, AW and ND). Free-text fields (see Table 1) were

For peer review only - http://bmjopen.bmj.com/site/about/guidelines.xhtml

extracted from the master file containing all LeDeR review data, and entered into a separate Microsoft Excel spreadsheet. The authors followed the Standards for Reporting Qualitative Research (SRQR) guidelines [26]..

Table 1 shows the parts of the initial review which were read and coded by the researchers for information about issues with DNACPR recommendations being followed. All parts of the review considered by the researchers were free-text boxes.

Table 1. Parts of the LeDeR initial review used by researchers to search for information about DNACPR adherence for the qualitative thematic analysis

Review question/section	Information included here
**Summary of discussion with GP and/or review of GP notes**	Free text box where reviewers are asked to include key events of care and comment on aspects of care from the GP notes they feel are important.
**Conversation with another person**	This is a free text box summary of a discussion with another person and/or clinician involved in the care of the person who died.
**Pen Portrait**	The pen portrait is a free text box where reviewers summarize the person themselves, their preferences, important people to them and important life events.
**DNACPR comments "Please add any comments about the DNACPR recommendation"**	Free text box where reviewers can add comments about DNACPR.
**Issue, concern or potential problem (initial review)**	Free text box where reviewers extract learning from the review.
**Learning and actions: issues (focused review)**	As above.

Positionality

The research team had expertise in learning disability and end-of-life care. A reflexive approach was adopted throughout the study, with researchers engaging in ongoing critical reflection on

For peer review only

how their backgrounds, assumptions and experiences may have influenced the research process, including interpretation of the data.

Thematic analysis

(1) Data familiarisation

Both researchers independently read all reviews fully to immerse themselves in the data. This involved actively reading, reflecting, and re-reading the reviews several times.

(2) Generating codes

A flexible and iterative process was undertaken. Initial codes were generated from the data, primarily completed by one researcher (CR). Reflective discussions were then carried out with the second researcher (AW).

(3) Developing themes

Initial themes were reviewed and compared to identify relationships, overlaps and inconsistencies. Iterative coding using mind maps on paper refined codes into themes until saturation was reached.

(4) Refining mature themes

Mind maps were used to group, ungroup and regroup early themes into mature themes. The researchers agreed on names and descriptions that best captured each theme.

(5) Producing the analysis

The final analysis is presented in the results section below. Any names included in the quotes have been removed to prevent identification.

(6) Independent review of analysis

For further validation, an additional author (ND) independently reviewed the coded dataset and thematic structure. Where coding differed, the thematic structure was revised through iterative discussion.

For peer review only

Patient and public involvement

The LeDeR programme works with people with a learning disability through the Staying Alive and Well co-production group, which reviews findings, contributes lived-experience perspectives and helps disseminate the work, including producing accessible reports.

RESULTS

Demographics

The demographics of the 21 adults with a learning disability whose deaths were reviewed by LeDeR and judged by reviewers to have had DNACPR followed incorrectly are shown in Table 2. The mean age at death of the sample was 64.6 (SD = 9.5) years.

For peer review only

Table 2. Descriptive statistics of the demographics of the sample of 21 adults with a learning disability whose deaths were reviewed by LeDeR and judged by reviewers to have had DNACPR followed incorrectly. Numbers below five have been suppressed with an asterisk to preserve anonymity.

Demographic	N (%)
**Sex**	
Male	8 (38%)
Female	13 (62%)
**Ethnicity**	
White	20 (95%)
**Place of death**	
Hospital	8 (38%)
Usual residence	11 (52%)
Other	*
**Year of death**	
2021	8 (38%)
2022	13 (62%)

Overview of thematic analysis

Three main themes highlight key challenges in how DNACPR recommendations are followed in practice:

- **Responding to an emergency** – how people respond in urgent, high-pressure situations
- **Knowledge and awareness of DNACPR** – whether staff are aware of and/or understand the scope of DNACPR recommendations
- **Making and enacting DNACPR decisions** – how DNACPR recommendations are made and recorded, and the decisions to enact them

Across all three themes, two cross-cutting factors are identified: breakdowns in information sharing, and unclear or inaccessible documentation. Cross-cutting factors shaped how DNACPR recommendations were understood, communicated and enacted in practice. These factors contribute to conditions of uncertainty across the three themes and culminate in a default to life-preserving action: the initiation of CPR despite an active DNACPR recommendation being in place. These factors were identified across multiple cases and are embedded within the themes presented below.

A thematic map of the data is shown in Figure 1.

For peer review only - http://bmjopen.bmj.com/site/about/guidelines.xhtml

Themes

Responding to an emergency

This theme represents the challenges faced when responding to an emergency. Often emergencies were unexpected and took paid carers or health professionals by surprise. Several of the reviews commented on the sudden nature of the person's collapse, and how those around them responded instinctively and called for help from emergency services. It was emergency services involvement that sometimes triggered CPR, as those attending to the collapse would follow instructions from the call handlers.

> *"Staff were adrenaline fuelled in an emergency situation and followed 999 call handler's instructions" - Reviewer 1*

> *"It was a terrible shock to everyone when he was found unresponsive. CPR was carried out by staff until the paramedics came" (Reviewer 21)*

Here the cross-cutting factor of breakdowns in information sharing was particularly evident. DNACPR recommendations were not always communicated to emergency services, meaning that responders acted without knowledge of the recommendation.

> *"It is reported by [relative] that she was upset that resuscitation was being commenced as the DNACPR was not known to the ambulance service" - Reviewer 19*

In other cases, the cross-cutting factor of unclear or inaccessible documentation shaped how the emergency unfolded. Staff were aware of a DNACPR recommendation but were unable to locate the relevant documentation in time. The challenges of locating documentation were particularly evident in residential home settings, where records may not be as easily accessible as in clinical environments.

> *"Carers called emergency services and carried out CPR for approximately 10 minutes until they located the TEP and then ceased." - Reviewer 9*

CPR attempts were sometimes ceased once paramedics arrived and could access medical records and verify a person's DNACPR status. This information was not always readily accessible in residential or non-medical settings, leading to confusion and distress about how to proceed.

> *"After [the] paramedic obtained medical advice they found [the] records stated [the person] was not for resuscitation and attempts stopped" - Reviewer 5*

For peer review only - http://bmjopen.bmj.com/site/about/guidelines.xhtml

Knowledge and Awareness of DNACPR

The second theme is around the knowledge and awareness of DNACPR among those involved in caring for the person with a learning disability. Issues around knowledge and awareness of DNACPR were on two distinct levels.

First, there were concerns about people being unaware of a DNACPR recommendation was in place at all. Being unaware of an active, valid DNACPR meant those attending to the collapsed patient proceeded in the natural and appropriate way by commencing CPR.

> *"Staff denied any knowledge of a DNACPR" - Reviewer 17*

In other cases, this lack of awareness was compounded by the cross-cutting factor of unclear or inaccessible documentation. Systems intended to alert staff to DNACPR status were not effective. Documentation may have been present but was not clearly visible or accessible.

> *"CPR was commenced as records viewed by staff did not reflect an alert that she had a DNACPR in place, during the efforts to resuscitate her it was noted that the alert was now evident on her records so CPR stopped" - Reviewer 18*

Second, in some cases, individuals caring for the person with a learning disability struggled to understand what a DNACPR recommendation means in practice. When faced with a collapse, staff called emergency services and followed instructions, sometimes without confidence about how to apply a DNACPR recommendation. Reviewers commented on the distress this confusion caused for the people involved in the situation, and some examples highlighted the "panic" and "upset" felt, particularly by care home staff.

> *"CPR was commenced following instruction from 999. There was a ReSPECT form in place however there was a lot of confusion around this. It had not been clearly communicated with [relative] or care team." - Reviewer 20*

In this case, the cross-cutting factor of breakdowns in information sharing was evident – the DNACPR recommendation had not been communicated with members of the person's care team.

Making and enacting DNACPR decisions

The final theme relates to issues with making and enacting decisions about DNACPR, including concerns or disagreements about how the recommendation was made, and whether they should be followed in practice.

Several cases highlighted disagreements and uncertainty about the appropriateness of a DNACPR recommendation. One reviewer described a situation in which a person's family had agreed with a DNACPR recommendation, but the recommendation was changed following

For peer review only - http://bmjopen.bmj.com/site/about/guidelines.xhtml

objections from the care team. This breakdown in information sharing led to some confusion about the validity of the recommendation.

> *"The DNACPR completed at that time in discussion with her family advised no treatment due to her poor quality of life. This decision was changed following objections from her care team."- Reviewer 15*

In another example, the person collapsed due to an accident - not the progression of their condition - which prompted a reversal of an existing DNACPR recommendation. Uncertainty about how to proceed confused care home staff, who had to escalate the decision about enacting a DNACPR recommendation.

> *"It was recognised by the staff who found [Name] that she had a DNACPR, they phoned the on-call director who advised them to start CPR given the circumstances she had been found in." - Reviewer 15*

In other cases, issues centered around considering the wishes and feelings of the person with a learning disability, and whether these had been considered in deciding to make a recommendation.

> *"It was also noted that the Care Home paperwork (Covid-19 Hospital Summary) recorded that the patient said 'Doctors have previously put DNAR in place without asking me. I have made it clear that I want to be resuscitated'" - Reviewer 1.*

The cross-cutting factor of unclear or inaccessible documentation was particularly prominent in this theme. In several cases, DNACPR documentation was incomplete, unsigned, or lacked clarity about how the decision was reached or who was consulted.

> *"It does not appear that the GP signed off [Name]'s emergency health care plan or advance care plans, nor did they attend meetings where these were formulated." - Reviewer 12*

This review also exemplified the cross-cutting factor of breakdowns in information sharing, as the appropriate professionals were not involved or consulted in the decision-making process of making the recommendation.

In other cases, the validity of DNACPR recommendations was questioned due to documentation being perceived as outdated or no longer applicable.

> *"Ambulance log states DNACPR in place staff stated, " 'It is not valid as it is old'. Recorded that invalid DNACPR had been viewed." - Reviewer 13*

Where documentation was incomplete, unclear, or contested, professionals were required to make rapid decisions without confidence in the validity of the recommendation. Uncertainty

For peer review only - http://bmjopen.bmj.com/site/about/guidelines.xhtml

about whether the decision-making process had been followed appropriately (such as whether the right people had been consulted, or whether the recommendation was current) contributed directly to non-compliance, with CPR initiated.

DISCUSSION

This paper examined cases where DNACPR recommendations for people with a learning disability were not followed, resulting in CPR being administered. Through qualitative thematic analysis of reviews of deaths, the study identified three themes: responding to an emergency, knowledge and awareness of DNACPR, and decision-making processes. Across these themes, two cross-cutting factors were identified: breakdowns in information sharing and unclear or inaccessible documentation. These factors created uncertainty about the presence, validity or applicability of DNACPR recommendations in practice, contributing to a default to life-preserving action and the initiation of CPR despite an active DNACPR recommendation being in place.

Breakdowns in information sharing and documentation issues were central to these failures, shaping how DNACPR recommendations were interpreted and acted upon in practice. In emergencies where DNACPR documentation was unclear, inaccessible, or perceived as invalid, professionals often defaulted to life-saving interventions. This reflects the ethical dilemma those responding to a cardiac arrest face, balancing the need to adhere to documented end-of-life decisions and the professional duty to attempt life-saving treatment. Whilst challenges in locating and accessing DNACPR documentation in an emergency may affect any patient, people with a learning disability may be more vulnerable to these situations. People with a learning disability are more likely to live in residential or supported living settings and receive care from a range of professionals across multiple services [19], where medical documentation may not be as readily accessible, and those present may not have clinical training.

These findings are not unique to people with a learning disability. National reviews and empirical studies have shown that DNACPR recommendations are particularly vulnerable to non-compliance at the point of implementation, particularly during acute deterioration of health and out-of-hours care in emergency situations [5-27]. However, people with a learning disability are less likely to be directly involved in end-of-life discussions, more likely to rely on proxy decision-makers, and more vulnerable to assumptions about quality of life and prognosis [7, 28]. Our findings suggest that these existing inequities may amplify the risks associated with unclear communication and documentation. To improve practice around DNACPR recommendations for people with a learning disability, facilitating better communication between all those involved in care, alongside accessible and readily available documentation,

For peer review only

can reduce uncertainty in emergency situations and support DNACPR recommendations being followed as intended.

Previous research has indicated that misunderstanding of DNACPR recommendations is common amongst nursing professionals working in elderly adult care [24]. Our findings relating to the second theme were consistent with this but extend to social care staff such as those working in care homes. Understanding and knowledge issues are potentially key barriers to both following DNACPR recommendations and ensuring recommendations are appropriate. More work is needed to explore how health and social care professionals understand DNACPR recommendations and how this may affect the correct implementation of procedures.

Despite its relevance, the Mental Capacity Act [9] was rarely mentioned in reviewer comments, perhaps due to the limitations in the structure of the reviews themselves, which do not prompt formal reflection on the Mental Capacity Act. Failure to embed Mental Capacity Act principles may not only lead to inappropriate care, but also reinforce existing structural inequalities for people with a learning disability, particularly where assumptions are made about their ability to engage in these sensitive discussions at the end-of-life. This may further contribute to uncertainty in decision-making processes, particularly where roles and responsibilities of those involved in decision-making are unclear.

Strengths and limitations

A strength of this paper is that it provides rich qualitative findings which give insight into issues surrounding cases in which DNACPR recommendations are not followed for people with a learning disability, leading to CPR taking place. A rigorous qualitative approach was undertaken which identified key areas of improvement for practice around DNACPR in this population.

As a retrospective analysis of secondary data, findings are limited by the details that reviewers chose to share (or had available) about the events surrounding a death. Researchers did not have access to the DNACPR documentation and did not have firsthand accounts of the events surrounding situations in which CPR was considered. Although reviewers are experienced professionals who receive specialist training, accounts may be subject to bias. The data may not represent all groups, with ethnic minority communities potentially underrepresented. Findings should be interpreted as highlighting challenges in implementing DNACPR recommendations, rather than the appropriateness or quality of the decision-making around the original DNAPCR recommendation.

For peer review only - http://bmjopen.bmj.com/site/about/guidelines.xhtml

CONCLUSION

This study identifies factors surrounding cases where DNACPR recommendations are not followed. Unclear or inaccessible documentation and uncertainty about how decisions are made in making and enacting DNACPR recommendations, contributed to recommendations not being followed as intended. Addressing these issues is important for people with a learning disability, who often receive care across multiple services and may require additional support to ensure their wishes are heard and understood.

There is a need for targeted training for staff who may encounter situations in which a DNACPR recommendation will be actioned, including care home staff, support workers and emergency responders. Training should focus on understanding what a DNACPR recommendation means, how these recommendations are recorded and reviewed, and where to find the relevant documentation alerting professionals to a person's DNACPR status. Improving knowledge around DNACPR for among health and social care staff will support clearer communication across services involved in the care of people with a learning disability.

Health and social care professionals involved in DNACPR decisions should ensure that the principles of the Mental Capacity Act [9] are embedded in clinical practice, with meaningful and accessible involvement of the person. Best interest decisions should be well-recorded and involve all relevant parties, including family members, carers, and all professionals involved in care.

Future research should assess the extent to which DNACPR recommendations are in line with recent recommendations [18] and examine how the Mental Capacity Act [9] is being used in the DNACPR decision-making process for people with a learning disability.

For peer review only - http://bmjopen.bmj.com/site/about/guidelines.xhtml

Contributors

Christina Roberts is the guarantor and was responsible for the conceptualisation, methodology, data curation and formal analysis of the study. Nicola Ditzel contributed the methodology and formal analysis of the study. Adam White contributed to the methodology and formal analysis of the study. Rory Sheehan contributed to the conceptualisation, methodology, and supervision of the study. Andre Strydom contributed to the conceptualisation, methodology, and supervision of the study. Alex Ruck- Keene contributed to the methodology of the study. Umesh Chauhan contributed to the conceptualisation, methodology, and supervision of the study.

Funding

LeDeR (Learning from Lives and Deaths: People with a learning disability and autistic people), is funded by NHS England. For more details, see: https://leder.nhs.uk/about. The funders had no role in the analysis of data, preparation of this paper or decision to publish.

Competing interests

The authors report no conflicts of interest.

Ethical approval

LeDeR has approval from the UK Secretary of State for Health and Social Care to collect and analyse data compliant with Section 251 for the use of confidential patient information without consent for a specific purpose.

Data availability statement

No additional data available.

For peer review only

For peer review only - http://bmjopen.bmj.com/site/about/guidelines.xhtml

References

1. NHS England. Do not attempt cardiopulmonary resuscitation (DNACPR) and people with a learning disability and or autism [Internet]. [cited 2025 Nov 17]. Available from: https://www.england.nhs.uk/publication/dnacpr-and-people-with-a-learning-disability-and-or-autism/

2. van Gijn MS, Frijns D, van de Glind EMM, C van Munster B, Hamaker ME. The chance of survival and the functional outcome after in-hospital cardiopulmonary resuscitation in older people: a systematic review. Age Ageing [Internet]. 2014 July;43(4):456–63. Available from: http://dx.doi.org/10.1093/ageing/afu035

3. GMC. Cardiopulmonary resuscitation (CPR) [Internet]. 2024. Available from: https://www.gmc-uk.org/professional-standards/the-professional-standards/treatment-and-care-towards-the-end-of-life/cardiopulmonary-resuscitation-cpr

4. Tracey Vs. Cambridge University Hospitals NHS Foundfation Trust [Internet]. p. [2014] EWCA Ci33. Available from: https://www.judiciary.uk/wp-content/uploads/JCO/Documents/Judgments/r-v-tracey.pdf

5. Perkins GD, Griffiths F, Slowther A-M, George R, Fritz Z, Satherley P, et al. Do-not-attempt-cardiopulmonary-resuscitation decisions: an evidence synthesis. Health Serv Deliv Res [Internet]. 2016 Apr;4(11):1–154. Available from: http://dx.doi.org/10.3310/hsdr04110

6. Mockford C, Fritz Z, George R, Court R, Grove A, Clarke B, et al. Do not attempt cardiopulmonary resuscitation (DNACPR) orders: a systematic review of the barriers and facilitators of decision-making and implementation. Resuscitation [Internet]. 2015 Mar;88:99–113. Available from: http://dx.doi.org/10.1016/j.resuscitation.2014.11.016

7. Wagemans AMA, van Schrojenstein Lantman-de Valk HMJ, Proot IM, Bressers AM, Metsemakers J, Tuffrey-Wijne I, et al. Do-Not-Attempt-Resuscitation orders for people with intellectual disabilities: dilemmas and uncertainties for ID physicians and trainees. The importance of the deliberation process. J Intellect Disabil Res [Internet]. 2017 Mar;61(3):245–54. Available from: http://dx.doi.org/10.1111/jir.12333

8. Winspear v City Hospitals Sunderland NHS Foundation Trust [Internet]. p. [2015] EWHC 3250 (QB). Available from: https://www.bailii.org/ew/cases/EWHC/QB/2015/3250.html

9. Mental capacity Act 2005 [Internet]. Statute Law Database. [cited 2025 Nov 17]. Available from: https://www.legislation.gov.uk/ukpga/2005/9/contents

For peer review only - http://bmjopen.bmj.com/site/about/guidelines.xhtml

10. Stein GL. Providing palliative care to people with intellectual disabilities: services, staff knowledge, and challenges. J Palliat Med [Internet]. 2008 Nov;11(9):1241–8. Available from: http://dx.doi.org/10.1089/jpm.2008.0130

11. Tuffrey-Wijne I, Goulding L, Gordon V, Abraham E, Giatras N, Edwards C, et al. The challenges in monitoring and preventing patient safety incidents for people with intellectual disabilities in NHS acute hospitals: evidence from a mixed-methods study. BMC Health Serv Res [Internet]. 2014 Sept 24;14(1):432. Available from: http://dx.doi.org/10.1186/1472-6963-14-432

12. Public Health England. COVID-19: deaths of people with learning disabilities [Internet]. Gov.uk. 2020 [cited 2025 Nov 17]. Available from: https://www.gov.uk/government/publications/covid-19-deaths-of-people-with-learning-disabilities

13. Tapper J. Fury at 'do not resuscitate' notices given to Covid patients with learning disabilities. The guardian [Internet]. 2021 Feb 13 [cited 2025 Oct 15]; Available from: https://www.theguardian.com/world/2021/feb/13/new-do-not-resuscitate-orders-imposed-on-covid-19-patients-with-learning-difficulties

14. Fitton E, Kuylen M, Wyllie A, Michalowski S, Bhatt V, Martin W. Managing DNACPR recommendations in residential care: Towards improved training for social care and capacity professionals. Br J Soc Work [Internet]. 2023 May 16; Available from: http://dx.doi.org/10.1093/bjsw/bcad078

15. Murdoch C, Banks R, Kanani N. Coronavirus » Do Not Attempt Cardiopulmonary Resuscitation DNACPR letter 3 April 2020 [Internet]. [cited 2025 Nov 17]. Available from: https://www.england.nhs.uk/coronavirus/documents/do-not-attempt-cardiopulmonary-resuscitation-dnacpr-letter-3-april-2020/

16. Powis S, May R, Kanani N. Coronavirus » Do Not Attempt Cardiopulmonary Resuscitation (DNACPR) and people with a learning disability and or autism [Internet]. [cited 2025 Nov 17]. Available from: https://www.england.nhs.uk/coronavirus/documents/c1146-do-not-attempt-cardiopulmonary-resuscitation-dnacpr-and-people-with-a-learning-disability-and-or-autism/

17. Powis S, Doyle A, Wee B, Banks R, May R. Coronavirus » Do Not Attempt Cardiopulmonary Resuscitation DNACPR letter 3 April 2020 [Internet]. [cited 2025 Nov 17]. Available from: https://www.england.nhs.uk/coronavirus/documents/do-not-attempt-cardiopulmonary-resuscitation-dnacpr-letter-3-april-2020/

For peer review only - http://bmjopen.bmj.com/site/about/guidelines.xhtml

18. Parliamentary and Health Service Ombudsman. End of life care: improving DNACPR conversations for everyone [Internet]. 2024 [cited 2025]. Available from: https://www.ombudsman.org.uk/publications/end-life-care-improving-dnacpr-conversations-everyone

19. White A, Sheehan R, Ding J, Roberts C, Magill N, Keagan-Bull R, et al. Learning from Lives and Deaths - People with a learning disability and autistic people (LeDeR) report for 2021 [Internet]. UCLan - University of Central Lancashire. London, UK; 2022 [cited 2025 Nov 17]. Available from: https://clok.uclan.ac.uk/43444/?template=default_internal

20. White A, Sheehan R, Ding J, Roberts C, Magill N, Keagan-Bull R, et al. LeDeR Learning from lives and deaths -People with a learning disability and autistic people Annual Report [Internet]. 2021. Available from: https://www.kcl.ac.uk/ioppn/assets/fans-dept/leder-main-report-hyperlinked.pdf

21. Wong J, Duane PG, Ingraham NE. A case series of patients who were do not resuscitate but underwent cardiopulmonary resuscitation. Resuscitation [Internet]. 2020 Jan 1;146:145–6. Available from: http://dx.doi.org/10.1016/j.resuscitation.2019.11.020

22. Obrien H, Brady SS, Harkin K, Kenny RA, Moriarty J. Do-not-attempt-resuscitation (DNAR) orders: understanding interpretation use hospitalised patient Ireland. brief report. Journal Medical Ethics. 2017;44(3):201–3.

23. Robinson F, Cupples M, Corrigan M. Implementing a resuscitation policy for patients at the end of life in an acute hospital setting: qualitative study. Palliat Med [Internet]. 2007 June;21(4):305–12. Available from: http://dx.doi.org/10.1177/0269216307077817

24. Nolan J, Robertson T, Donaldson J. A Mixed-Methods Study of DNACPR in Nursing Practice In partial fulfilment of the degree of Clinical Doctorate in Nursing. 2022 [cited 2025 Nov 17]; Available from: https://dspace.stir.ac.uk/bitstream/1893/35023/1/A

25. Braun V, Clarke V. One size fits all? What counts as quality practice in (reflexive) thematic analysis? Qual Res Psychol [Internet]. 2021 July 3;18(3):328–52. Available from: http://dx.doi.org/10.1080/14780887.2020.1769238

26. O'Brien BC, Harris IB, Beckman TJ, Reed DA, Cook DA. Standards for reporting qualitative research: a synthesis of recommendations. Acad Med [Internet]. 2014 Sept;89(9):1245–51. Available from: http://dx.doi.org/10.1097/ACM.0000000000000388

27. Findlay GP, Shotton H, Kelly K, Mason M. Time to Intervene? A review of patients who underwent cardiopulmonary resuscitation as a result of an in-hospital cardiorespiratory arrest [Internet]. 2012. Available from: https://www.ncepod.org.uk/2012report1/downloads/CAP_fullreport.pdf

For peer review only - http://bmjopen.bmj.com/site/about/guidelines.xhtml

28. Noorlandt HW, Echteld MA, Tuffrey-Wijne I, Festen DAM, Vrijmoeth C, van der Heide A, et al. Shared decision-making with people with intellectual disabilities in the last phase of life: A scoping review. J Intellect Disabil Res [Internet]. 2020 Nov;64(11):881–94. Available from: http://dx.doi.org/10.1111/jir.12774

For peer review only

542 **FIGURE TITLE**

543 **Figure 1.** Thematic map

For peer review only

For peer review only - http://bmjopen.bmj.com/site/about/guidelines.xhtml

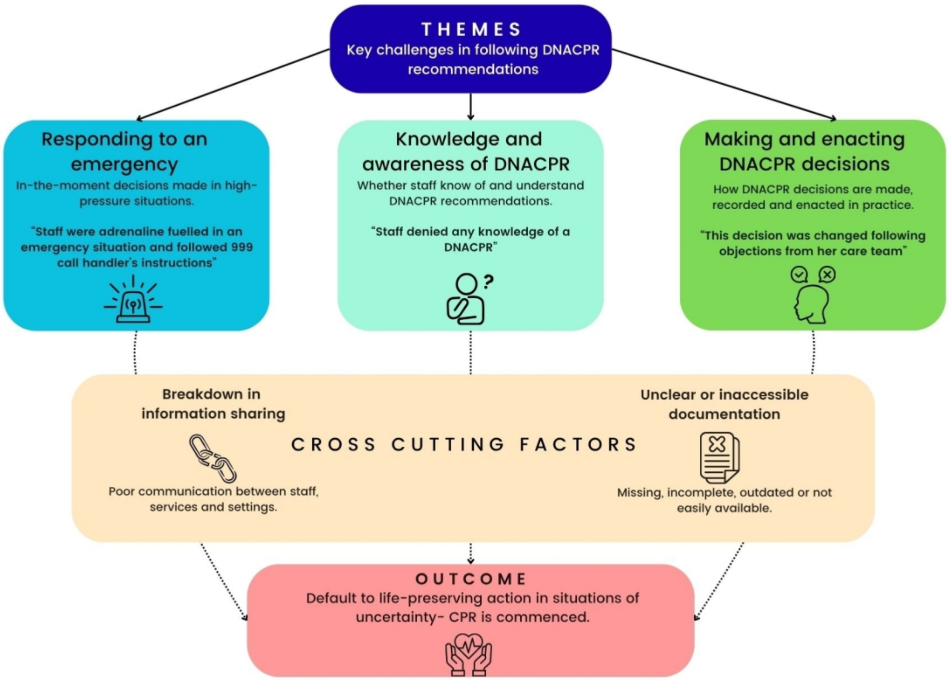

Figure 1: A thematic map of the three main themes and two cross-cutting factors.

151x104mm (300 x 300 DPI)

Supplementary Table 1: Reviewer judgment of whether DNACPR documentation was correctly completed or followed for reviews of deaths in 2020 to 2022 of people with a learning disability with a DNACPR recommendation in place.

Was the DNACPR documentation correctly completed and followed?	2020	2021	2022	Total
**Correctly Completed and Followed**	52.0% (n = 92)	60.9% (n = 1,144)	63.3% (n = 979)	61.5% (n = 2,215)
**Correctly Completed but Was Not Followed**	*	1.1% (n = 21)	1.2% (n = 19)	*
**Incorrectly Completed and Followed**	11.9% (n = 21)	6.2% (n = 116)	7.1% (n = 109)	6.8% (n = 246)
**Neither Completed nor Followed Correctly**	*	0.6% (n = 11)	0.8% (n = 13)	*
**Don't know**	35.0% (n = 62)	31.2% (n = 585)	27.6% (n = 426)	29.8% (n = 1,073)
**Total**	177	1,877	1,546	3,600

For peer review only
